# An illustrated key to the species of *Gasteruption* Latreille (Hymenoptera, Gasteruptiidae) from Palaearctic China, with description of four new species

**DOI:** 10.3897/zookeys.1038.64978

**Published:** 2021-05-19

**Authors:** Jiang-Li Tan, Cornelis van Achterberg, Jia-Xuan Wu, Hang Wang, Qi-Jing Zhang

**Affiliations:** 1 Shaanxi Key Laboratory for Animal Conservation / Key Laboratory of Resource Biology and Biotechnology in Western China, College of Life Sciences, Northwest University, 229 North Taibai Road, Xi’an, Shaanxi 710069, China Northwest University Xi'an China; 2 State Key Laboratory of Rice Biology and Ministry of Agriculture / Key Lab of Agricultural Entomology, Institute of Insect Sciences, Zhejiang University, Hangzhou 310058, China Zhejiang University Hangzhou China

**Keywords:** Inner Mongolia, new record, Ningxia, Shaanxi, Xinjiang

## Abstract

Four new species of the genus *Gasteruption* Latreille, 1797 (Hymenoptera: Evanioidea: Gasteruptiidae: Gasteruptiinae) are described from China. Three are from Shaanxi (NW China; *G. granulatum* Tan & van Achterberg, **sp. nov.**, *G. pedion* Tan & van Achterberg, **sp. nov.**, and *G. reductum* Tan & van Achterberg, **sp. nov.**) and one from S China and Ningxia (*G. kexinae* Tan & van Achterberg, **sp. nov.**). Eleven species are newly recorded for Shaanxi (*G. abeillei* Kieffer, 1912, *G. amoyense* Pasteels, 1958, *G. bimaculatum* Pasteels, 1958, *G. corniculigerum* Enderlein, 1913, *G. latitibia* Zhao, van Achterberg & Xu, 2012, *G. minutum* (Tournier, 1877), *G. nigritarse* (Thomson, 1883), *G. parvicollarium* Enderlein, 1913, *G. sinarum* Kieffer, 1911, *G. subtile* (Thomson, 1883) and *G. brevicuspis* Kieffer, 1911). The newly-recorded species and the new species are keyed and illustrated. Two new synonyms are proposed: *G. rufescenticorne* Enderlein, 1913, with *G. japonicum* Cameron, 1888, **syn. nov.** and *G. oriplanum* Kieffer, 1911, with *G. minutum* (Tournier, 1877), **syn. nov.**

## Introduction

Gasteruptiidae are very slender apocritan hymenopterans with elongated “neck” (propleuron), swollen hind tibiae and compressed petiolate metasoma attached very high on the propodeum. Up to now, 511 species are recognised as valid in two subfamilies, Gasteruptiinae (with four genera) (Macedo 2009, 2011; Zhao et al. 2012) and Hyptiogastrinae (with two genera) (Jennings and Austin 2002).

The adults frequently feed on flowers with easily accessible nectar (especially families Apiaceae, Asteraceae and Euphorbiaceae), but likely at least some *Gasteruption* species feed on both nectar and pollen (Jennings and Austin 2004) and extra-floral nectaries are also visited (fig. 20.204 in van Breugel 2014). Gasteruptiidae are also known by their hovering inspection flight in front of bee nests (van Achterberg 2013; Miko et al. 2019), balancing with their enlarged hind tibiae. The gasteruptiid larvae feed on the larval food of solitary bees, after consuming the egg or larva of the bee (Malyshev 1965) and, therefore, are best named predator-inquilines (inquilines because they use the bee nest for shelter and predate on the owner(s)). They select bees of the subfamilies Apidae, Colletidae and Megachilidae, nesting in stems, galls or in wood and also (but less often) vertical soil substrates (e.g. clay banks) may be used (Zhao et al. 2012; van Achterberg 2013; van Breugel 2014; Bogusch et al. 2018). As far as known, bees nesting in horizontal soil substrates are far less attacked; an exception is reported from Australia where members of the Hyptiogastrinae do attend bee nests on flat ground (Houston 1987). *Gasteruption* species use primarily bee nests (Malyshev 1965; van Breugel 2014; Bogusch et al. 2018; Parslow et al. 2020), based only on indirect evidence. Secondarily, they may use the contents of spheciform or vespid wasp cells present in the same habitat (Crosskey 1951; Hanson and Gauld 1995, 2006; Jennings and Austin 1997a, 1997b, 2004; Parslow et al. 2020). This will likely be the case if there is a limited supply of bee nests. Metamorphosis takes place inside the host’s nest where the gasteruptiid pupa hibernates until the next spring or summer (Malyshev 1965; He 2004; Jennings and Austin 2004; van Breugel 2014).

All known gasteruptiids from the Palaearctic Region belong to the subfamily Gasteruptiinae and to the genus *Gasteruption* Latreille, 1797. Up to 2018, 33 species were known from China, of which seven were found in the NW Chinese Province Shaanxi, which is 21% of the total for China. However, Tan et al. (2016) estimated that the real number will be around 14 spp. or about 60% of the total number of species known from China.

## Material and methods

The specimens were collected by sweep nets or in Malaise traps. The material was directly killed and stored in 70% ethanol and subsequently prepared according to the AXA method (van Achterberg 2009; van Achterberg et al. 2010) and glued on card points.

Observations and descriptions were made with an Opto-Edu A230903 stereomicroscope and a fluorescent lamp. Photographic images were made with the Keyence VHX-5000 digital microscope and processed with Adobe Photoshop CS5, mostly to adjust the size and background. For the identification, Zhao et al. (2012) and Tan et al. (2016) were used, together with information on the type series of previously-described species gathered by the second author.

The antesternal carina (van Achterberg in Zhao et al. 2012; van Achterberg 2013; van Achterberg and Talebi 2014) is the lamelliform upcurved anterior ridge of the mesopleuron (directly behind the base of the fore coxa; “asc”, in Fig. 308); in many species, the anterior ridge is not or only slightly lamelliform and straight. The term antennal segment is used as the synonym of antennomere. The middle of the vertex should be in the plane of the objective of the binocular microscope. For the other terminology, see Zhao et al. (2012). Measurements are performed as indicated in Figs 308, 309 and in van Achterberg (1988). Additional non-exclusive characters in the key are between square brackets.

The following abbreviations are used for the depositories:

**NWUX**College of Life Sciences, Northwest University, Xi’an;

**RMNH**Naturalis Biodiversity Center, Leiden;

**SCAU**South China Agricultural University, Guangzhou;

**BZL**Oberösterreichisches Landesmuseum, Biologiezentrum, Linz;

**MNHN**Muséum National d’Histoire Naturelle, Paris;

**MHNG**Muséum d’Histoire Naturelle, Genève;

**ZIL** Zoological Institute, Lund;

**ZJUH**Institut of Insect Sciences, Zhejiang University, Hangzhou.

## Taxonomy

### Gasteruption

Taxon classificationAnimaliaHymenopteraGasteruptiidae

Latreille, 1797

7A7AC7FA-4A97-556A-A823-4B3D5E660C27

Figs 1–2 , 3 , 4–12 , 13–17 , 18–26 , 27–28 , 29–37 , 38–40 , 41–49 , 50–56 , 57–62 , 63–71 , 72–79 , 80–82 , 83–92 , 93–97 , 98–106 , 107–108 , 109–117 , 118–123 , 124–127 , 128–136 , 137–138 , 139–143 , 144 , 145–154 , 155–156 , 157–165 , 166–167 , 168–173 , 174–175 , 176–187 , 188 , 189–194 , 195–197 , 198–207 , 208–212 , 213–214 , 215–223 , 224–225 , 226–231 , 232–234 , 235–245 , 246–252 , 253 , 254–263 , 264–271 , 272–275 , 276–284 , 285–291 , 292–298 , 299–307

#### Remarks.

*Gasteruption* Latreille, 1797: 113; Zhao et al. 2012: 6–7 (diagnosis, references, key); van Achterberg 2013: 59 (key to Dutch spp.); Jennings and Parslow 2014: 95 (Australia); van Achterberg and Talebi 2014: 10 (illustrated key Iran and Turkey); Žikić et al. 2014: 573 (distribution in former Yugoslavia); van Breugel 2014: 424–432 (*Gasteruption* in bee hotels); Mao et al. 2014: 1864; Jennings et al. 2015: 399 (New Caledonia); Tan et al. 2016: 53 (illustrated key Pal. China); Johansson and van Achterberg 2016: 74 (*G. assectator* aggregate); Lotfalizadeh et al. 2017: 144 (Iran); Saure et al. 2017: 191 (Arabian Peninsula); Bogusch et al. 2018: 4 (*Gasteruption* spp. in reed galls); Orlovskytė et al. 2018: 119 (checklist Lithuania). Type-species (designated by Latreille 1810): *Ichneumon assectator* Linnaeus, 1758.

##### Key to species of the genus *Gasteruption* Latreille from Palaearctic China

**Table:** 

1	Ovipositor present (a); antenna with 14 segments (b) (females)	**2**
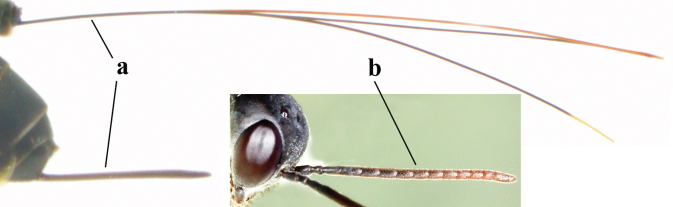		
–	Ovipositor absent (aa); antenna with 13 segments (bb) (males); [if males are unknown, the species is provisionally inserted]	**38**
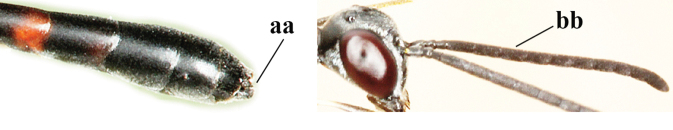		
2	Apex of ovipositor sheath blackish or dark brown; if narrowly pale apically, then white, ivory or brownish-yellow part at most 0.3× as long as hind basitarsus (a); (intermediate species are included in both alternatives)	**3**
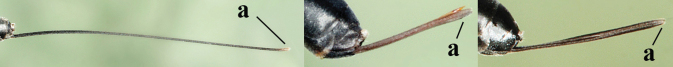		
–	Apex of ovipositor sheath distinctly white or ivory (but rarely pale brown) and pale part 0.3–8.0× as long as hind basitarsus (aa)	**23**
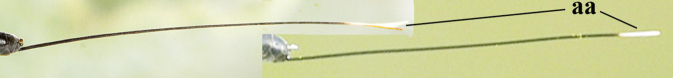		
3	Ovipositor sheath 0.6–2.0× as long as hind tibia and 0.3–1.2× as long as hind tibia and tarsus combined (a); incision of hypopygium shallow V-shaped and up to apical 0.2 (b) or absent; occipital carina obsolescent to narrowly lamelliform medio-dorsally (c)	**4**
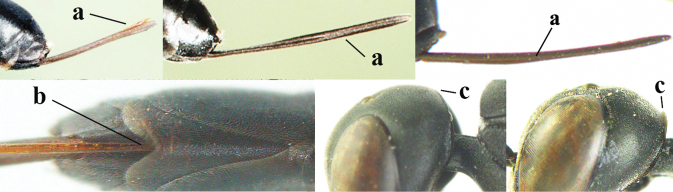		
–	Ovipositor sheath 3.0–7.0× as long as hind tibia and 1.9–4.0× as long as hind tibia and tarsus combined (aa); incision of hypopygium often deep and slit-like up to apical 0.3–0.5 (bb); occipital carina obsolescent (c) or distinctly lamelliform (cc) medio-dorsally	**16**
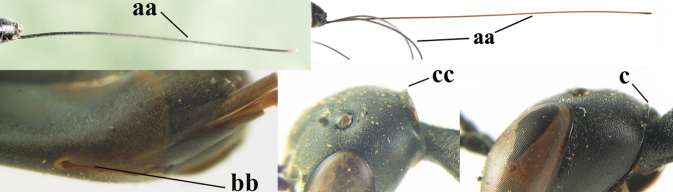		
4	Head in anterior view protruding below lower level of eyes, 0.5–0.6× length of second antennal segment and 0.4–0.6× basal width of mandible and mandibular condylus distinctly below lower level of eyes (a); in lateral view condylar incision of malar space remains far removed from eye (b); ovipositor sheath 0.4–0.9× as long as hind tibia (c)	***G. minutum* (Tournier, 1877)**
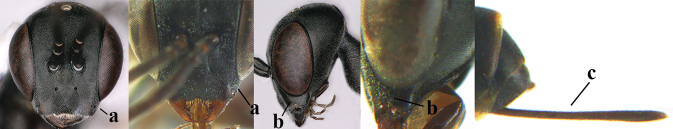		
–	Head in anterior view slightly protruding below lower level of eyes by less than half basal width of mandible and mandibular condylus near lower level of eyes (aa); in lateral view condylar incision of malar space close to eye (bb), rarely slightly wider; ovipositor sheath 0.7–2.7× as long as hind tibia (cc)	**5**
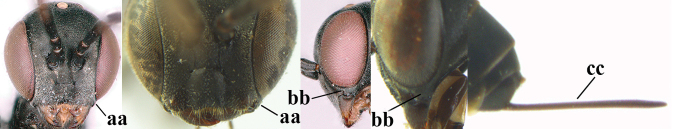		
5	Clypeus with rather large shallow depression (a); mesoscutum densely reticulate-rugulose or -rugose (b); hind basitarsus stout (c); apical antennal segment 1.4–1.6× third antennal segment (d); [ovipositor sheath 0.6–1.5× as long as hind tibia]	***G. hastator* (Fabricius, 1804)**
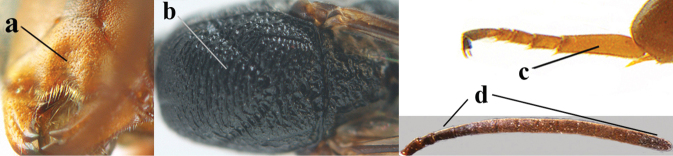		
–	Clypeus with small depression or depression obsolescent (aa); mesoscutum mainly densely coriaceous or rugulose (bb); hind basitarsus more slender (cc), rarely similarly stout; apical antennal segment at most 1.2× as long as third antennal segment (dd)	**6**
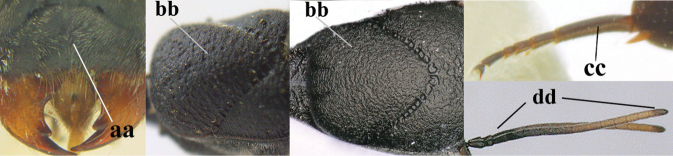		
6	Mesoscutum coarsely (often “crater”-like) punctate (a); head distinctly emarginate medio-posteriorly (b); head less protruding in lateral view (c) and narrower in anterior view (d)	**7**
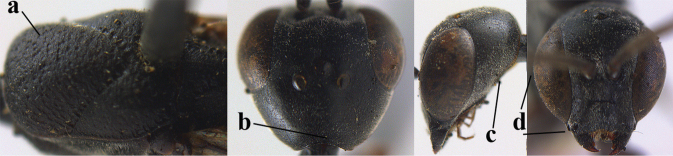		
–	Mesoscutum predominantly densely coriaceous, at most with some shallow punctures (aa); head truncate medio-posteriorly or nearly so (bb); head more protruding in lateral view (cc) and wider in anterior view (dd)	**8**
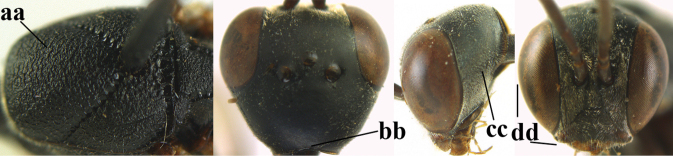		
7	Hind tibia about as long as hind femur and trochanter combined or slightly longer (a); head somewhat longer in dorsal (b) and lateral (c) view; head directly narrowed behind eyes in dorsal view (d)	***G. formosanum* Enderlein, 1913**
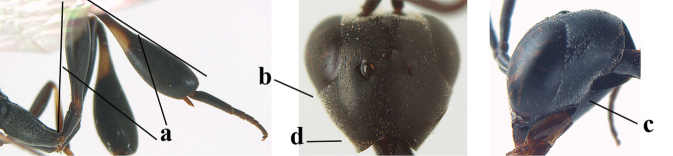		
–	Hind tibia 1.1–1.2× as long as hind femur and trochanter combined (aa); head somewhat shorter in dorsal (bb) and lateral (cc) view; head roundly narrowed behind eyes in dorsal view (dd)	***G. sinicola* (Kieffer, 1924)**
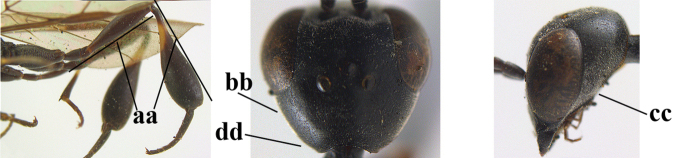		
8	Vertex strongly bulging above upper level of eyes (a); head comparatively long in dorsal view (b); ovipositor sheath 1.2–1.7× as long as hind tibia **and** apex of ovipositor wide and with distinct dorsal teeth (c); propleuron slightly less robust in lateral view (d); [hind tibia moderately slender; propleuron antero-dorsally and pronotum ventrally coriaceous; pronotum convex antero-ventrally]	***G. parvicollarium* Enderlein, 1913**
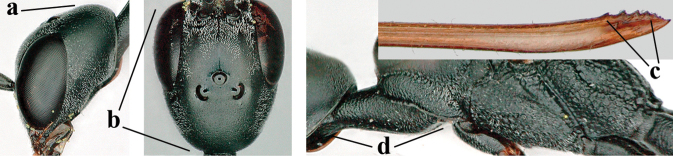		
–	Vertex at most moderately bulging above upper level of eyes (aa); head shorter in dorsal view (bb); ovipositor sheath usually shorter; **if** 1.2–1.9× longer than hind tibia, **then** apex of ovipositor narrow and with minute dorsal teeth (cc); propleuron robust in lateral view (dd)	**9**
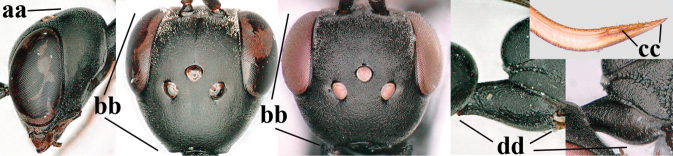		
9	Hind tibia slender (a); pronotal sides antero-dorsally granulate (b); side of pronotum slender and with narrow and weakly crenulated grooves (c); ovipositor sheath 1.3–1.9× as long as hind tibia; [hind basitarsus elongate; hind tibia dark brown to yellowish-brown ventro-basally]	***G. granulatum* sp. nov.**
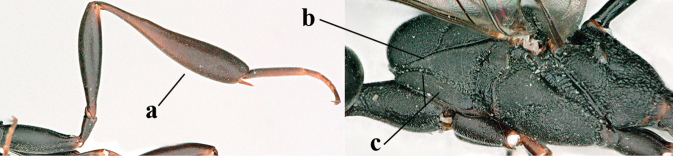		
–	Hind tibia distinctly inflated (aa); pronotal sides antero-dorsally coriaceous or rugulose (bb); side of pronotum robust and with wider and distinctly crenulated grooves (cc); ovipositor sheath 0.7–1.4× as long as hind tibia	**10**
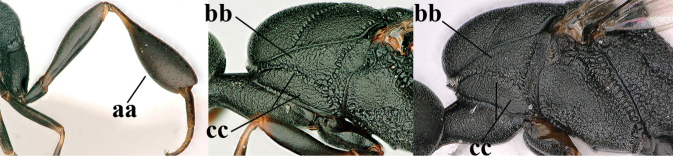		
10	Mandible black, dark brown or reddish-brown basally (a), rarely narrowly so and largely brownish-yellow; basal depression of mandible rather large and deep (b); tegula dark brown or brown (c); fifth (= pre-apical) sternite dark brown, blackish or narrowly pale medio-apically (d)	**11**
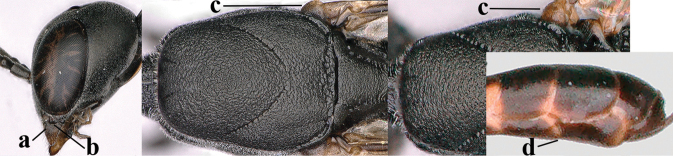		
–	Mandible pale yellow basally (aa); basal depression of mandible often smaller and shallower (bb); tegula yellow (cc), brownish-yellow or brown (ccc); fifth sternite more or less yellowish medio-apically (dd)	**14**
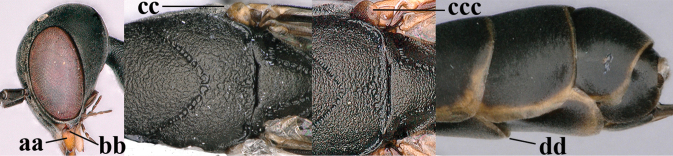		
11	Head in dorsal view directly narrowed posteriorly and longer (a); vertex moderately protruding above eye in lateral view (b); mesopleuron more elongated (c)	***G. latitibia* Zhao, van Achterberg & Xu, 2012**
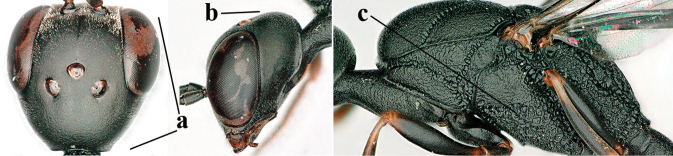		
–	Head in dorsal view rounded narrowed posteriorly and shorter (aa); vertex less protruding above eye in lateral view (bb); mesopleuron less elongated (cc)	**12**
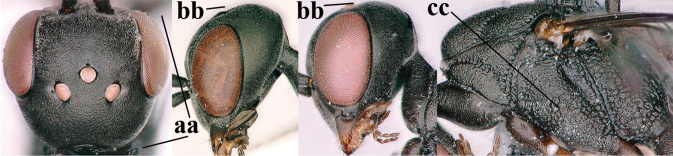		
12	Ovipositor sheath with curved bristles (“velcro”-type; a); lateral buccal area shallowly depressed (b); hypostomal bridge often longer (behind buccal area at underside of head; c); [pronotum antero-laterally evenly finely sculptured; POL 1.7–2.3× width of anterior ocellus]	***G. nigritarse* (Thomson, 1883)**
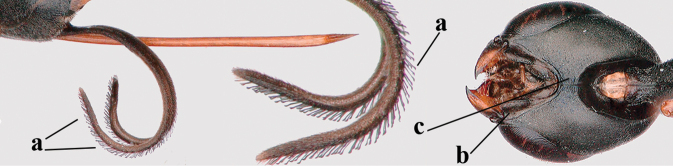		
–	Ovipositor sheath with normal straight setae, either largely bristly and erect (aa) or mostly adpressed and finely setose (aaa); lateral buccal area distinctly depressed (bb); hypostomal bridge usually shorter (bb); [sculpture of pronotum antero-laterally variable]	**13**
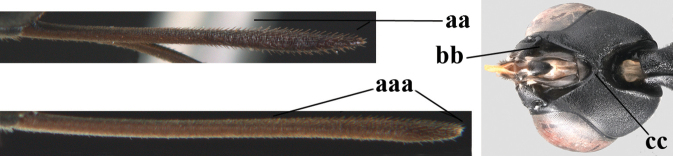		
13	Ovipositor sheath medially only with short adpressed pale setae at 50× (lacking upward directed dark brown and short bristly setae) and subapically slightly bristly setose (a); ovipositor sheath not or slightly widened subapically (b), 0.9–1.4× as long as hind tibia (only to measure if fully exserted); second and third antennal segments usually more robust (c); occipital carina wider latero-dorsally (d); [medially sculpture of mesoscutum at 60× variable, often dissimilar to very fine sculpture of vertex and more or less rugulose]	***G. assectator* (Linnaeus, 1758)**
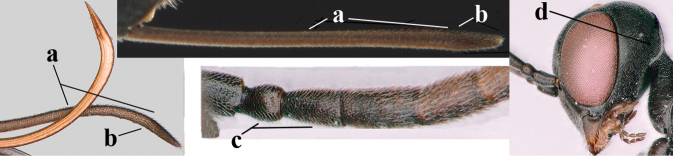		
–	Ovipositor sheath medially with erect setae or bristles at 50× and subapically distinctly bristly setose (aa); ovipositor sheath widened subapically (bb), 0.6–1.1× as long as hind tibia; second and third antennal segments usually more slender (cc); occipital carina narrower latero-dorsally (dd); [medially sculpture of mesoscutum at 60× variable, frequently similar to fine sculpture of vertex or somewhat coarser	***G. abeillei* Kieffer, 1912**
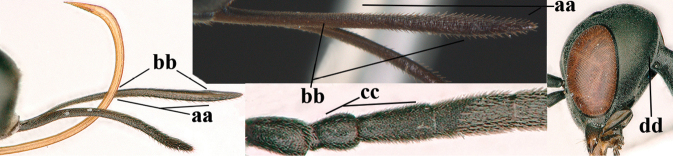		
14	Hind basitarsus rather stout and at least partly ivory dorsally (a); mesoscutum somewhat coarser sculptured (b); fifth metasomal sternite widely pale yellowish posteriorly (c); [hind tibia dark ventrally and hind femur black]	***G. flavimarginatum* van Achterberg, 2014**
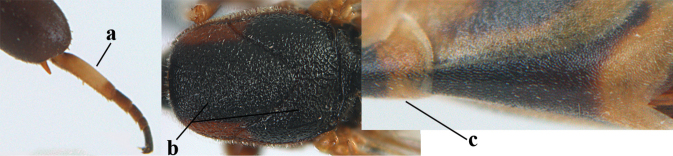		
–	Hind basitarsus slender and entirely dark brown or brown dorsally (aa); mesoscutum finely sculptured (bb); fifth sternite narrowly pale yellowish posteriorly or mainly dark brown (cc)	**15**
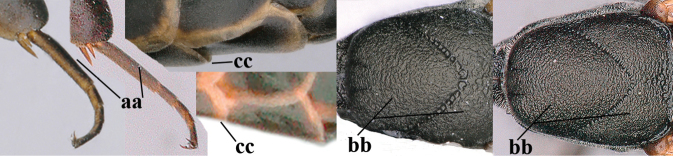		
15	Posterior ocellus situated near upper level of vertex (a); head in dorsal view directly narrowed posteriorly (b); ovipositor sheath 0.5–0.7× as long as hind tibia (c); [hind femur often partly dark reddish-brown; malar space 0.2–0.3× basal width of mandible]	***G. bicoloratum* Tan & van Achterberg, 2016**
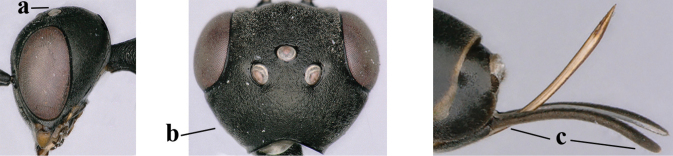		
–	Posterior ocellus situated distinctly below upper level of vertex (aa); head in dorsal view gradually narrowed posteriorly (bb); ovipositor sheath 0.8–1.1× as long as hind tibia (cc)	***G. brevicuspis* Kieffer, 1911**
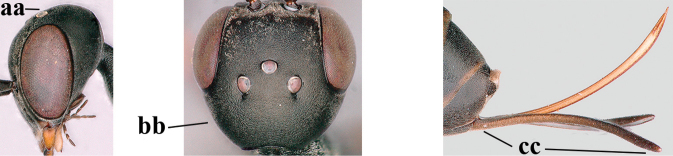		
16	Vertex of ♀ with reversed V-shaped emargination medio-posteriorly (a; emargination hardly developed in ♂), flat (b) **and** smooth, shiny and long dorsally (c); mesoscutum of ♀ finely transversely rugose to nearly smooth (d); [dorsal apical teeth of ovipositor distinct]	***G. bimaculatum* Pasteels, 1958**
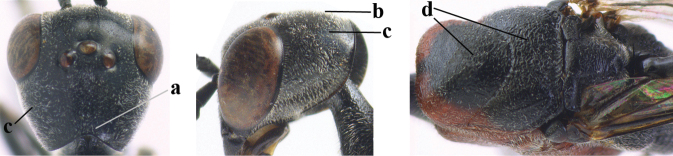		
–	Vertex of ♀ truncate medio-posteriorly (aa) or reversed U-shaped emarginate (aaa), shorter and moderately convex (bb); **if** vertex more or less emarginate and/or flat, then vertex finely sculptured, with satin sheen and shorter (cc); mesoscutum of ♀ punctate, punctate-rugose or transversely wrinkled (dd)	**17**
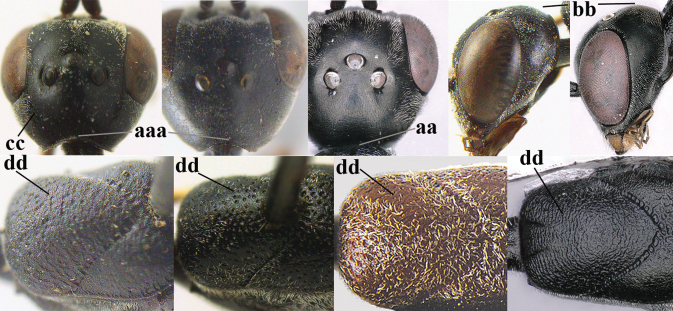		
17	Head rather elongate and below eyes slightly enlarged, minimum length of malar space 0.3–0.4× second antennal segment (a); head distinctly reversed U-shaped emarginate medio-posteriorly (b); mandible and malar space brown (c); hind tarsus brownish apically, paler than basally (d); [apex of ovipositor sheath ivory; first metasomal tergite orange or yellowish-brown]	***G. dimidiatum* Semenov, 1892**
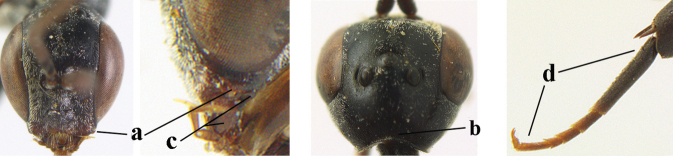		
–	Head less elongate and below eyes not enlarged, minimum length of malar space 0.1–0.2× second antennal segment (aa); head shallowly emarginate medio-posteriorly (bb); if intermediate (bbb), then mandible brownish-yellow and contrasting with colour of malar space (cc); apically hind tarsus as dark brown as basally (dd); [apex of ovipositor sheath dark brown or black]	**18**
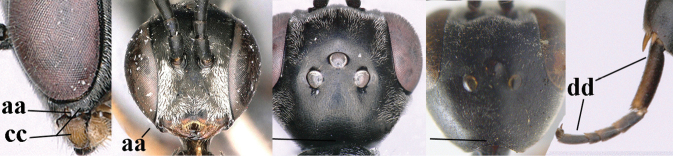		
18	Mesosoma sparsely setose laterally (a); pale apical part of ovipositor sheath 0.3–1.0× as long as hind basitarsus, apex ivory, brownish-yellow or brown (b); hind femur dark brown or black (c)	**19**
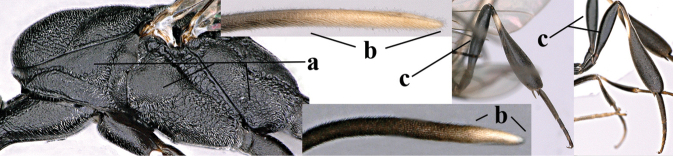		
–	Mesosoma densely setose laterally (aa); apex of ovipositor sheath blackish or mainly dark brown (bb); **if** apex pale, then at most 0.3× as long as hind basitarsus (bbb); hind femur orange or reddish-brown (cc), but black in *G. shengi*	**20**
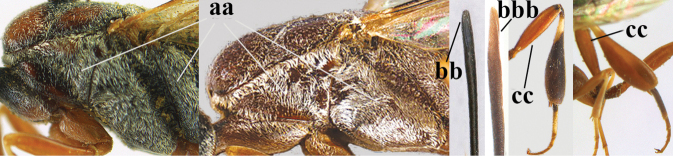		
19	Middle lobe of mesoscutum coarsely punctate (a); vertex strongly convex (b); mandible largely blackish or dark brown (c); mesosoma less elongated (d)	***G. sinarum* Kieffer, 1911**
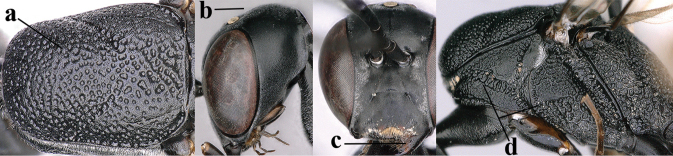		
–	Middle lobe of mesoscutum without punctures and finely transversely wrinkled (aa); vertex moderately convex (bb); mandible yellowish (cc); mesosoma distinctly elongated (dd)	***G. pannuceum* Tan & van Achterberg, 2016**
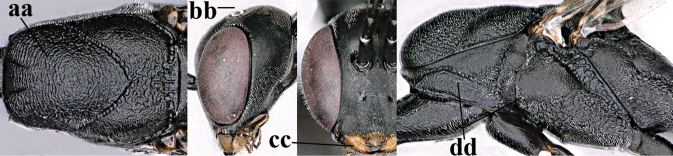		
20	Hind tibia more slender (a); first metasomal tergite dark brown (b); middle lobe of mesoscutum remotely punctate (c); hind femur black or blackish-brown (d); mesoscutum and head black (e)	***G. shengi* Tan & van Achterberg, 2016**
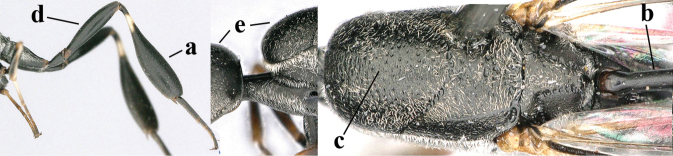		
–	Hind tibia more inflated (aa), but intermediate in *G. argentifrons* and *G. coloratum* (21bb); first tergite reddish or orange (bb); middle lobe of mesoscutum densely punctate or punctate-rugose (cc); hind femur orange brown to dark brown (dd), but partly or entirely black in *G. coloratum*; mesoscutum often paler than head (ee) or both reddish	**20**
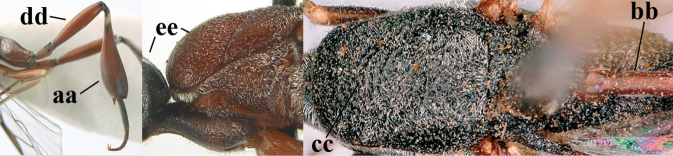		
21	Ovipositor sheath 3.1–4.4× as long as hind tibia (a); hind tibia more inflated (b); vertex longer setose (c); mesosoma largely or entirely black laterally (d); [hind femur and tibia (except basally) similarly coloured, orange brown or dark brown]	***G. dilutum* Semenov, 1892**
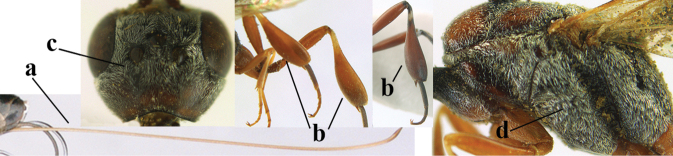		
–	Ovipositor sheath approx. 7.0× as long as hind tibia (aa); hind tibia less inflated (bb); vertex shorter setose (cc); mesosoma often dark reddish or orange brown laterally (dd)	**22**
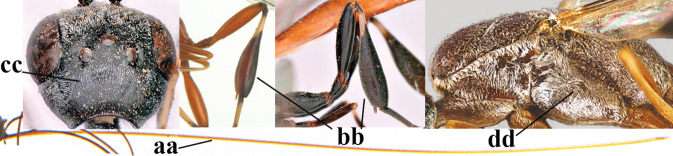		
22	Basal half of hind coxa mainly transversely rugose or punctate dorsally (a); apex of ovipositor sheath largely dark brown or brown (b); pronotum longer setose (c); [hind coxa orange brown or blackish]	***G. coloratum* Zhao, van Achterberg & Xu, 2012**
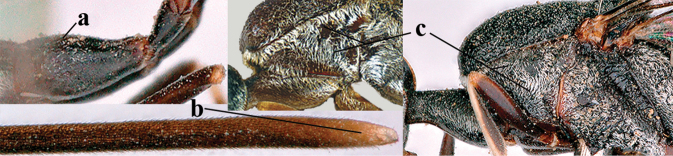		
–	Basal half of hind coxa superficially coriaceous dorsally (aa); apex of ovipositor sheath ivory or brownish-yellow (bb); pronotum shorter setose (cc)	***G. argentifrons* Semenov-T.-S. & Kostylev, 1928**
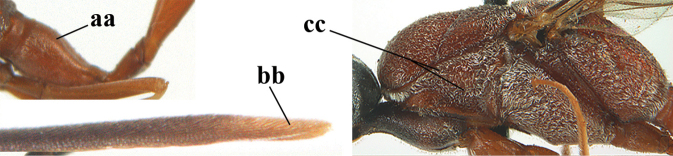		
23	Head with medial depression in front of occipital carina and with pair of lateral depressions (a); **if** shallow, then head in dorsal view nearly parallel-sided behind eyes (b); occipital carina wide lamelliform medio-dorsally (c)	**24**
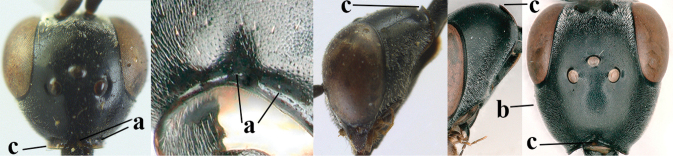		
–	Head flat or evenly convex in front of occipital carina (aa); **if** with a shallow depression in front of occipital carina (aaa), then head directly narrowed behind eyes (bb) and occipital carina at most moderately lamelliform medio-dorsally (cc)	**26**
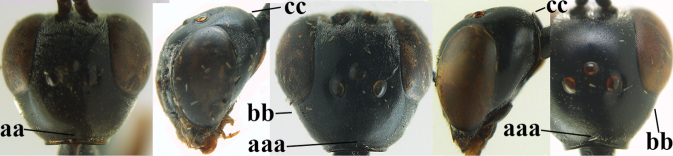		
24	Propleuron moderately robust and 0.8–1.0× as long as mesoscutum in front of tegula (a); vertex medially weakly convex in lateral view (b); head shorter in dorsal view (c); mandible often pale in anterior view medially (d); [white or ivory part of ovipositor sheath 1.7–3.3× longer than hind basitarsus]	***G. oshimense* Watanabe, 1934**
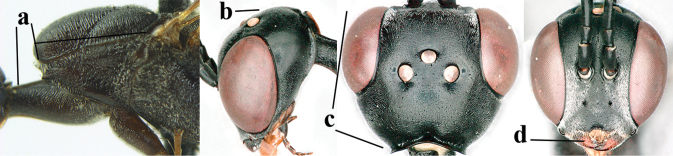		
–	Propleuron slender and 1.0–1.2× as long as mesoscutum in front of tegula (aa); vertex medially nearly flat in lateral view (bb); head comparatively long in dorsal view (cc); mandible often darker in anterior view (dd)	**25**
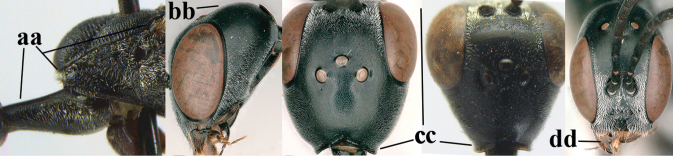		
25	Head in dorsal view distinctly narrowed behind eyes (a); middle lobe of mesoscutum with fine transverse elements anteriorly (b; sometimes coarsely sculptured); propleuron in ventral view less slender anteriorly (c); white or ivory apical part of ovipositor sheath 2.4–3.8× longer than hind basitarsus (d)	***G. corniculigerum* Enderlein, 1913**
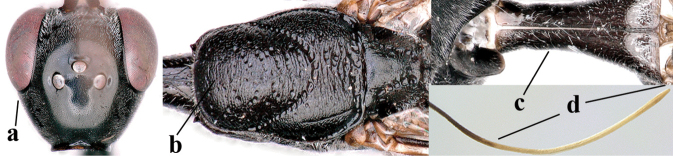		
–	Head in dorsal view nearly parallel-sided behind eyes (aa); middle lobe of mesoscutum only coriaceous between punctures (bb); propleuron in ventral view more slender anteriorly (cc); white or ivory apical part of ovipositor sheath 1.0–2.1× longer than hind basitarsus (dd)	***G. kexinae* sp. nov.**
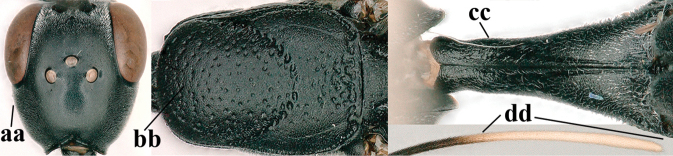		
26	Ovipositor sheath comparatively wide and about 0.9× as long as hind tibia, 0.3× as long as metasoma and 0.2× as long as body (a); middle lobe of mesoscutum rather protuberant in lateral view (b); pronotal tooth slender and acute	***G. assectoides* Zhao, van Achterberg & Xu, 2012**
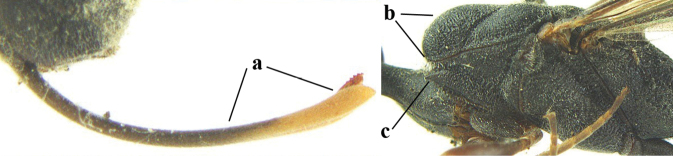		
–	Ovipositor sheath comparatively narrow and 1.1–9.0× as long as hind tibia, 0.6–2.8× as long as metasoma and 0.4–1.4× as long as body (aa); middle lobe of mesoscutum less protuberant in lateral view (bb); if convex (bbb), then pronotal tooth wider and rather blunt (cc)	**27**
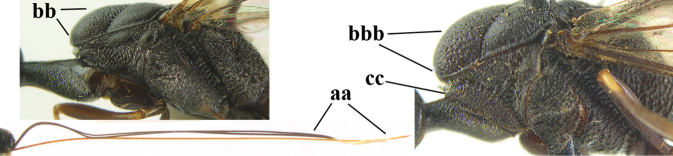		
27	Ovipositor about 0.4× as long as body and 0.6× as long as metasoma (a); hind coxa very slender (b); ovipositor widened apico-ventrally and more or less angularly up-curved apically in dead specimens (c)	***G. angulatum* Zhao, van Achterberg & Xu, 2012**
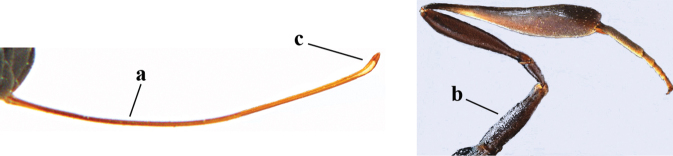		
–	Ovipositor 0.8–1.4× as long as body and 1.2–1.9× as long as metasoma (aa); hind coxa slightly less slender (bb); ovipositor narrow apico-ventrally and nearly straight or gradually up-curved apically (cc)	**28**
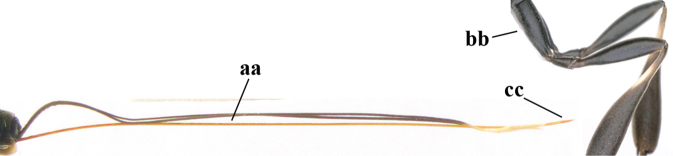		
28	Hind femur and tibia widened (a); hind basitarsus robust (b); head slightly narrowed in dorsal view (c); head slender in anterior view (d) and face narrower than clypeus (e); hind basitarsus entirely dark brown (f); [ovipositor sheath about 1.4× as long as body and 8.5× as long as hind tibia]	***G. huangshii* Tan & van Achterberg, 2016**
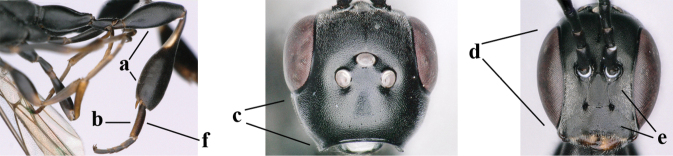		
–	Hind femur and tibia slender (aa); hind basitarsus more slender (bb); head in dorsal view distinctly narrowed (cc); head in anterior view subglobular (dd); **if** slender (ddd), then face as wide as clypeus (ee); hind basitarsus often partly ivory (ff)	**29**
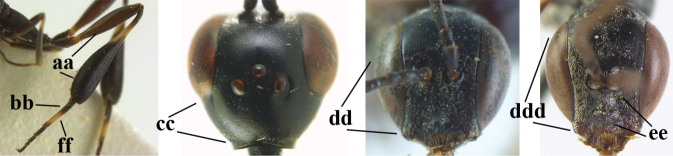		
29	Pale apical part of ovipositor sheath 0.2–1.0× as long as hind basitarsus (a); occipital carina non-lamelliform medio-dorsally (b) **and** mesoscutum distinctly punctate or rugose (c); **if** narrow lamelliform (bb), then hypopygium yellowish-brown or yellow; [ovipositor sheath 1.0–1.3× as long as body]	**30**
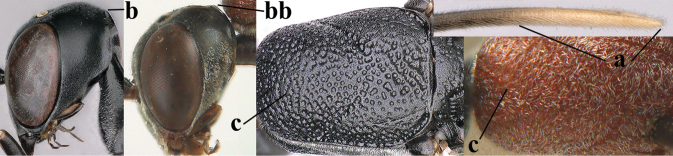		
–	Pale apical part of ovipositor sheath 1.1–3.5× as long as hind basitarsus (aa); occipital carina narrow lamelliform medio-dorsally (bb) and hypopygium dark brown or black; **if** non-lamelliform (bbb), then mesoscutum very finely coriaceous and, at most, punctulate (cc) or transversely rugulose	**32**
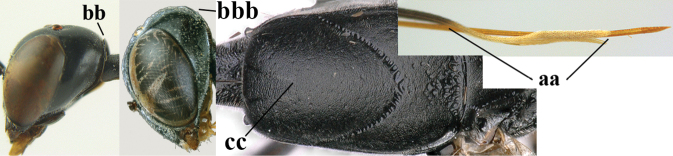		
30	Hypopygium dark brown or black (a); mesoscutum between coarse punctures with satin sheen and densely punctulate (b); head longer in dorsal view (c); face with short setosity laterally (d); [mandible dark brown or brown in anterior view]	***G. sinarum* Kieffer, 1911**
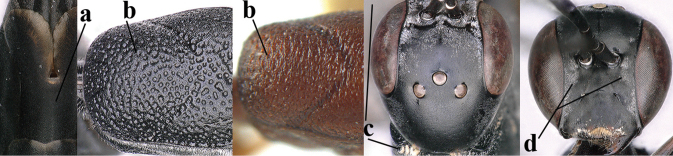		
–	Hypopygium yellowish-brown or yellow (aa); mesoscutum between punctures rather shiny and sparsely punctulate (bb); head shorter in dorsal view (cc); face with long setosity laterally (dd)	**31**
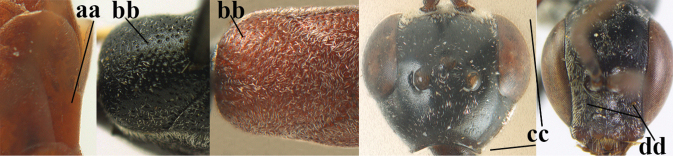		
31	Head slightly elongated ventrally (a); mandible largely dark brown in anterior view (b); mesosoma dorsally black (c), hind coxa (d), hind femur (e) and hind basitarsus (f) dark brown	***G. dimidiatum* Semenov, 1892**
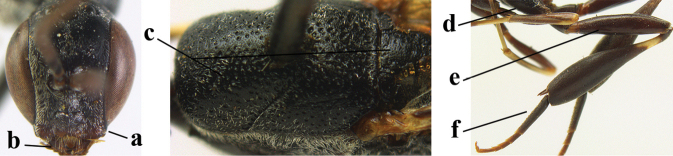		
–	Head short ventrally (aa); mandible yellow in anterior view (bb); mesosoma dorsally (cc), hind coxa (dd) and femur (ee) reddish or orange brown; hind basitarsus mainly ivory (ff)	***G. argentifrons* Semenov T.-S. & Kostylev, 1928**
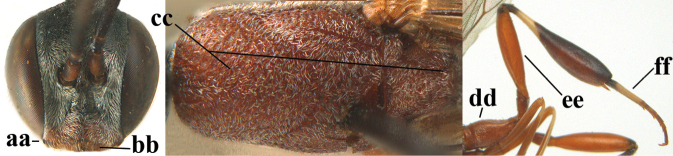		
32	Notauli with transverse rugae posteriorly and shallow (a) and anteriorly narrow, finely crenulate or nearly smooth (b); middle lobe of mesoscutum very finely transversely rugulose (c) **and** vertex (d) dull and finely coriaceous (as temple dorsally (e))	***G. reductum* sp. nov.**
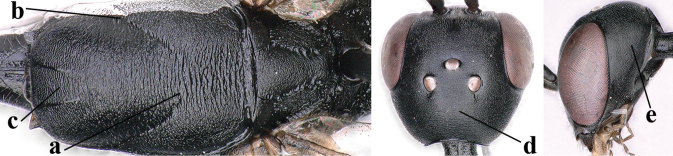		
–	Notauli only crenulate posteriorly and distinctly impressed (aa) and anteriorly wider and moderately crenulate (bb); middle lobe of mesoscutum very finely coriaceous (cc); **if** finely transversely rugulose, then vertex (dd) and temple dorsally (ee) shiny and largely smooth	**33**
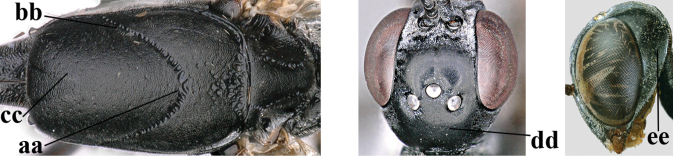		
33	Occipital carina non-lamelliform medio-dorsally and narrow laterally (a); head gradually narrowed in dorsal view (b) and short (c); vertex in lateral view more (d) or less (dd) above level of ocelli; head dorsally with satin sheen (e)	**34**
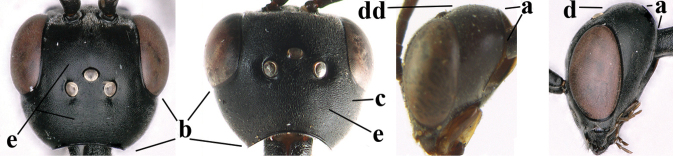		
–	Occipital carina narrowly lamelliform medio-dorsally and moderately wide laterally (aa); head usually directly narrowed posteriorly in dorsal view (bb); **if** gradually narrowed (bbb), then head longer (cc); vertex in lateral view near level of ocelli (dd); head dorsally more or less shiny (ee)	**35**
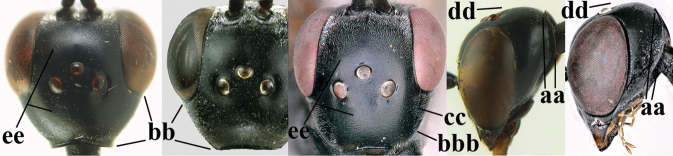		
34	Mesoscutum densely punctate (a) and punctulate-coriaceous between punctures (b); pronotal side more or less rugose or rugulose ventrally (c); white or ivory apical part of ovipositor sheath 1.1–1.9× as long as hind basitarsus (d)	***G. subtile* (Thomson, 1883)**
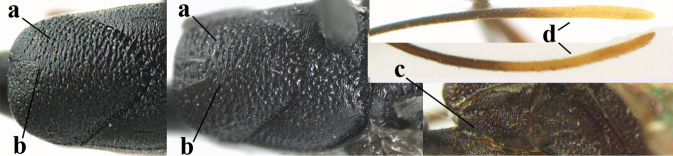		
–	Mesoscutum at most sparsely punctulate (aa) and very finely coriaceous between minute punctures (bb); pronotal side entirely finely coriaceous ventrally (cc); white or ivory apical part of ovipositor sheath 2.1–2.6× as long as hind basitarsus (dd)	***G. pedion* sp. nov.**
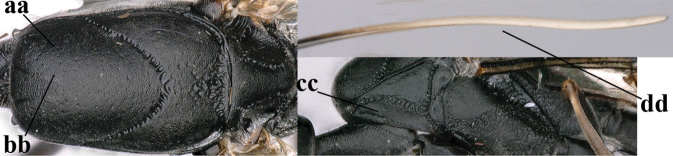		
35	Head elongate-elliptical in dorsal view (a); propleuron 1.1–1.2× as mesoscutum in front of tegula (b); mesosoma more slender in lateral view (c); mesoscutum less convex dorsally (d); hind tibia slender (e)	***G. amoyense* Pasteels, 1958**
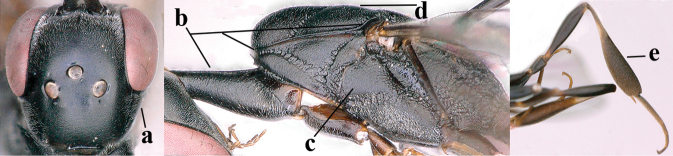		
–	Head trapezoid in dorsal view (aa); propleuron 0.9–1.0× mesoscutum in front of tegula (bb); mesosoma less slender in lateral view (cc); mesoscutal lobes distinctly convex dorsally (dd); hind tibia more robust (ee) or slender (e)	**36**
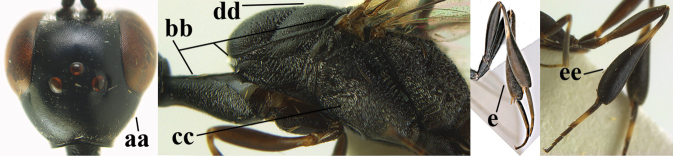		
36	Pale apical part of ovipositor sheath 3.0–3.5× as long as hind basitarsus (a); fourth antennal segment 1.7–2.3× as long as third antennal segment (b); vertex shiny and largely smooth or finely punctulate (c), without shallow depression medio-posteriorly (d) and in lateral view distinctly convex (e); [hind basitarsus black]	***G. tonkinense* Pasteels, 1958**
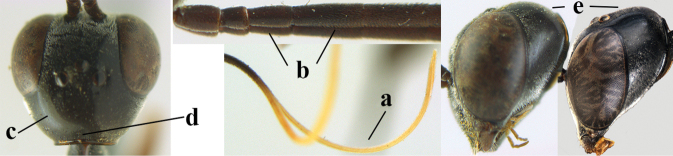		
–	Pale apical part of ovipositor sheath 1.1–2.4× as long as hind basitarsus (aa); fourth antennal segment 1.2–1.9× as long as third antennal segment (bb); head dorsally with satin sheen and finely sculptured (cc), rather often with shallow depression medio-posteriorly (dd; *G. japonicum*) and vertex less convex (ee)	**37**
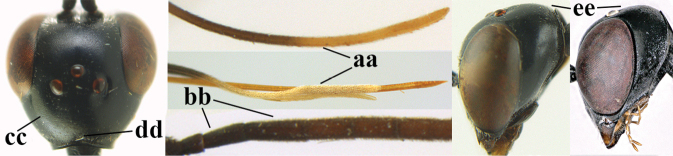		
37	Lobes of mesoscutum distinctly convex in lateral view (a); middle mesoscutal lobe more protuberant in dorsal (b) and lateral (c) view; fourth antennal segment 1.7–1.9× as long as third segment (d); hind tibia more slender (e); [occipital carina fine and non-lamelliform dorsally; vertex in front of occipital carina without depression]	***G. sinepunctatum* Zhao, van Achterberg & Xu, 2012**
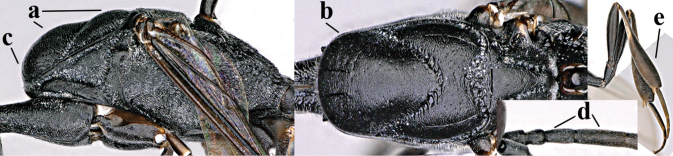		
–	Lobes of mesoscutum comparatively flat in lateral view (aa); middle mesoscutal lobe less protuberant in dorsal (bb) and lateral (cc) view; fourth antennal segment 1.2–1.5× as long as third segment (dd); hind tibia less slender (ee); [hind basitarsus often partly ivory]	***G. japonicum* Cameron, 1888**
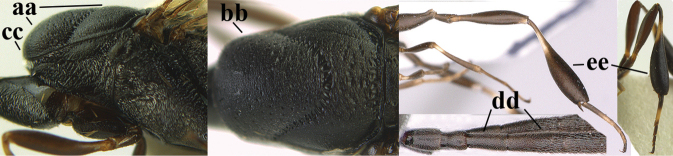		

**Males**

**Table:** 

38	Vertex with distinct medio-posterior depression or a groove with two minute tubercles in front of distinctly lamelliform occipital carina (a); mesoscutum medially transversely or obliquely rugulose, in large specimens transversely rugose (b); occipital carina wide lamelliform (c)	**39**
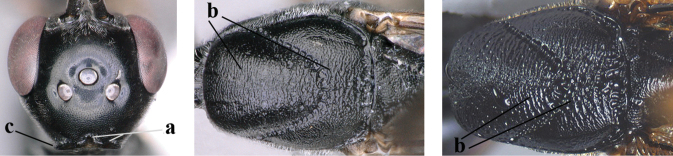		
–	Vertex flat or evenly convex in front of occipital carina (aa); **if** slightly depressed (aaa), then mesoscutum mainly punctate medially (bb) or occipital carina narrow lamelliform (cc)	**41**
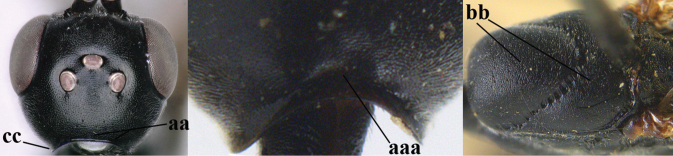		
39	Propleuron moderately robust and 0.8–1.0× as long as mesoscutum in front of tegula (a); fifth antennal segment 2.2–2.9× as long as third segment (b); head shorter in dorsal view (c)	***G. oshimense* Watanabe, 1934**
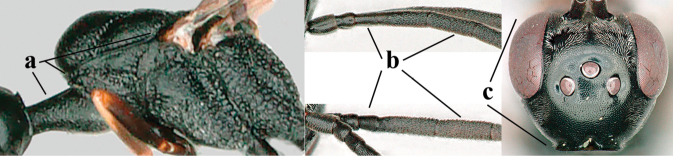		
–	Propleuron slender and 1.0–1.2× as long as mesoscutum in front of tegula (aa); fifth antennal segment 1.8–2.1× as long as third segment (bb); head comparatively long in dorsal view (cc)	**40**
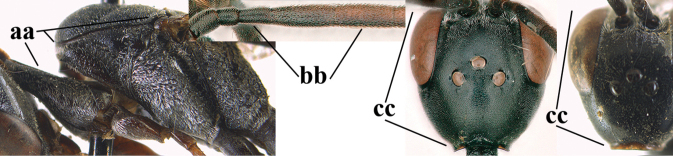		
40	Middle lobe of mesoscutum with transverse elements (a); mesoscutum distinctly rugose medio-posteriorly (b); hind femur slightly more slender (c)	***G. corniculigerum* Enderlein, 1913**
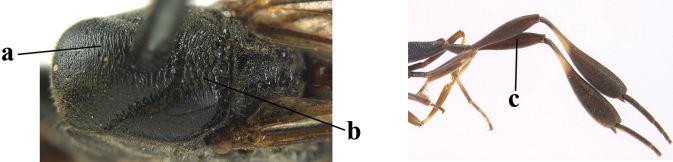		
–	Middle lobe of mesoscutum without transverse elements, only coriaceous between punctures (aa); mesoscutum only punctate medio-posteriorly (bb); hind femur slightly widened (cc)	***G. kexinae* sp. nov.**
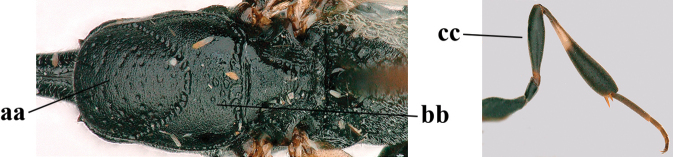		
41	Clypeus with rather large shallow depression (a); hind basitarsus rather stout (b); mesoscutum reticulate or rugose (c); [head and scapus more or less orange or reddish-brown, but sometimes entirely black]	***G. hastator* (Fabricius, 1804)**
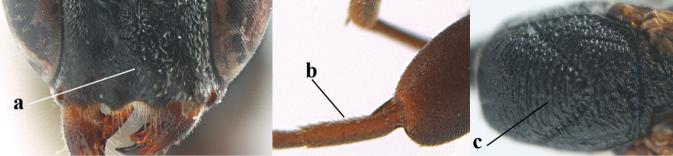		
–	Clypeus with small depression or depression obsolescent (aa); hind basitarsus often more slender (bb) or mesoscutum coriaceous or rugulose (cc)	**42**
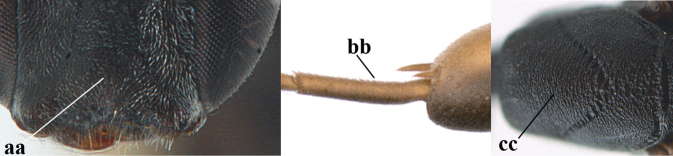		
42	Head in anterior view distinctly protruding below lower level of eyes (a), in lateral view condylar incision of malar space remains distinctly removed from eye, malar area behind indentation square and at least 0.8× as long as second antennal segment (= pedicellus) and 0.6–0.9× basal width of mandible (b); [mesoscutum densely coriaceous and matt, similar to vertex]	***G. minutum* (Tournier, 1877)**
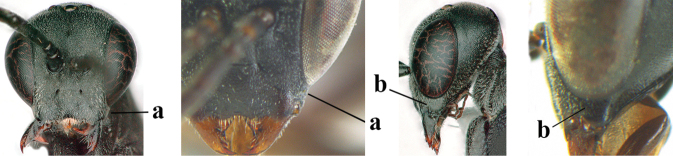		
–	Head in anterior view hardly protruding below lower level of eyes (aa), in lateral view condylar incision of malar space close to eye and malar area behind indentation transverse and 0.3–0.5× as long as second antennal segment and 0.2–0.3× basal width of mandible (bb), rarely slightly longer (aaa, bbb)	**43**
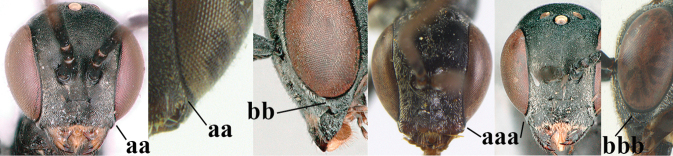		
43	Mesoscutum only coriaceous or finely rugulose medially (a), at most with a few shallow punctures; [♂ unknown of *G. assectoides*, *G. granulatum* and *G. pannuceum* and are provisionally included]	**44**
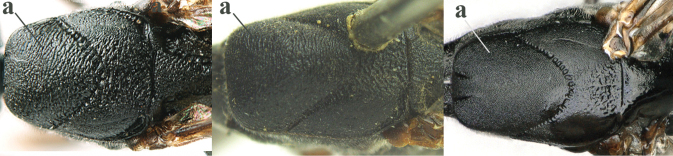		
–	Mesoscutum with several distinctly impressed punctures medially (aa; but often shallow in *G. japonicum*) or reticulate-rugose (aaa)	**60**
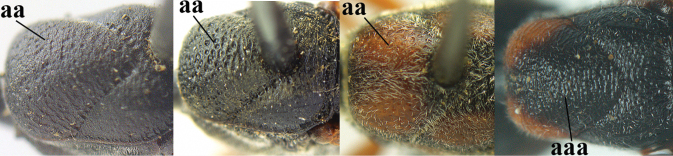		
44	Hind tibia slender, hardly to moderately inflated (a); mesoscutum often very finely and regularly sculptured (b)	**45**
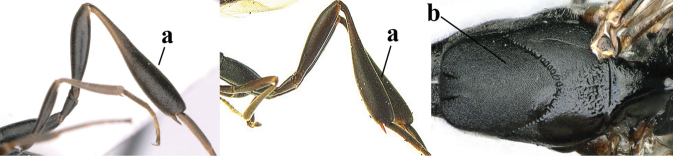		
–	Hind tibia distinctly inflated (aa); mesoscutum mainly densely coriaceous or irregularly rugulose (aa), especially near notauli (bb)	**54**
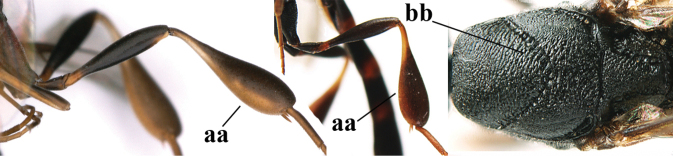		
45	Mesoscutum distinctly regularly transversely rugulose (a); hind tibial spurs yellowish and distinctly contrasting with dark hind basitarsus (b)	***G. assectoides* Zhao, van Achterberg & Xu, 2012**
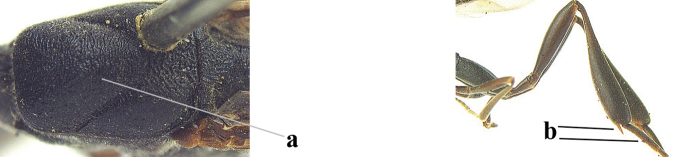		
–	Mesoscutum mainly finely coriaceous or superficially irregularly rugulose (aa); hind tibial spurs more or less brown and less contrasting with hind basitarsus (bb)	**46**
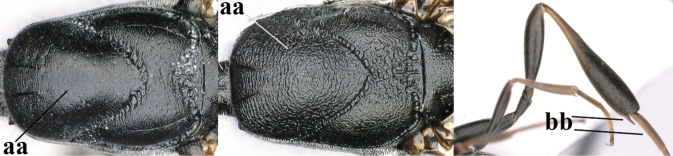		
46	Fourth antennal segment 1.8–3.5× as long as third segment (a); face rather narrow (b)	**47**
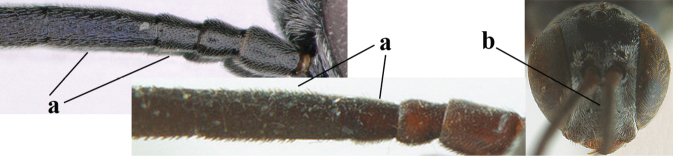		
–	Fourth antennal segment 1.4–1.7× as long as third segment (aa; unknown of *G. pannuceum* and *G. granulatum*); face wide (bb)	**50**
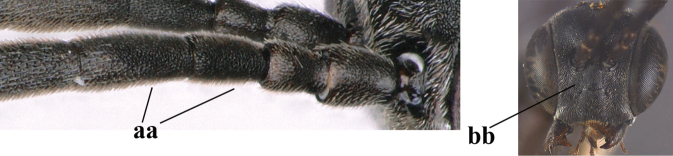		
47	Hind femur rather robust (a); second antennal segment (= pedicellus) more slender (b); middle lobe of mesoscutum less protuberant in lateral view (c); [fourth antennal segment 2.5–2.9 × as long as third segment]	***G. huangshii* Tan & van Achterberg, 2016**
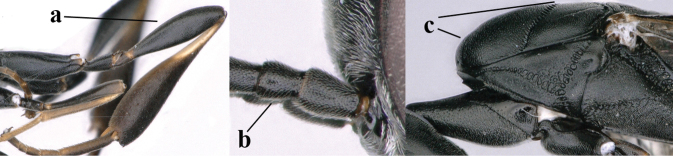		
–	Hind femur slender (aa); pedicellus robust (bb); middle lobe of mesoscutum more protruding in lateral view (cc)	**48**
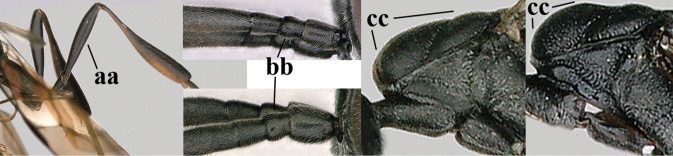		
48	Mandible medially yellow (a); fourth antennal segment 2.5–3.5× as long as third segment (b); vertex largely smooth or densely punctulate (c)	***G. sinepunctatum* Zhao, van Achterberg & Xu, 2012**
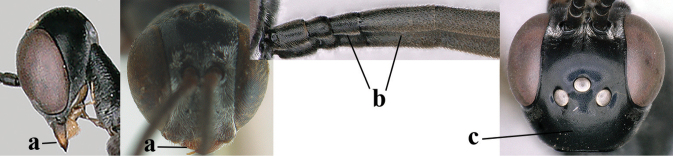		
–	Mandible medially black or dark brown (aa); fourth antennal segment 1.8–2.3× as long as third segment (bb); vertex coriaceous (cc)	**49**
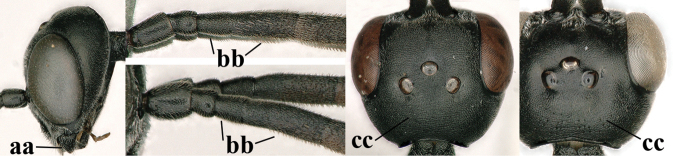		
49	Mesoscutum with fine transverse rugulae (a); propleuron less elongate (b); middle lobe of mesoscutum less protruding (c)	***G. reductum* sp. nov.**
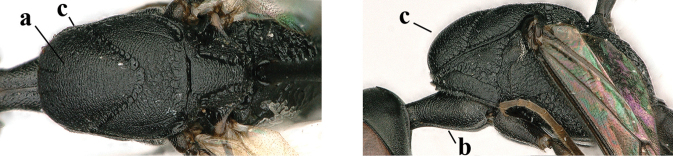		
–	Mesoscutum with transverse rugulae absent or with very fine rugulae (aa); propleuron more elongate (bb); middle lobe of mesoscutum more protruding (cc)	***G. pedion* sp. nov.**
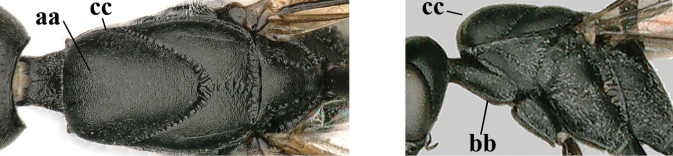		
50	Head strongly convex in lateral view (a); head longer in dorsal view (b); hind coxa and tibia rather robust (c); [mandible dark brown]	***G. parvicollarium* Enderlein, 1913 s. str.**
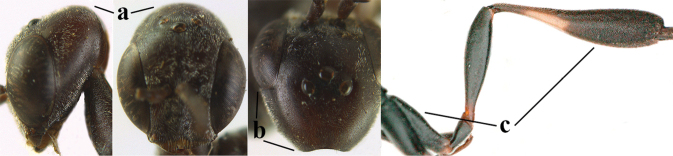		
–	Head moderately convex in lateral view (aa); head rather short in dorsal view (bb); hind coxa and tibia slender (cc)	**51**
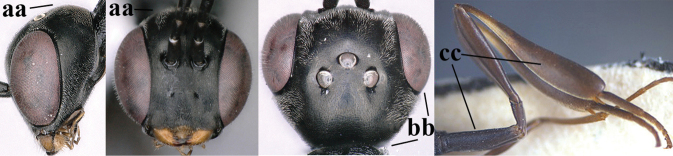		
51	Hind coxa distinctly narrower than hind tibia (a); hind femur narrower medially (b); mesosternal sulcus narrow and largely smooth (c); mandible dark brown basally (d)	***G. angulatum* Zhao, van Achterberg & Xu, 2012**
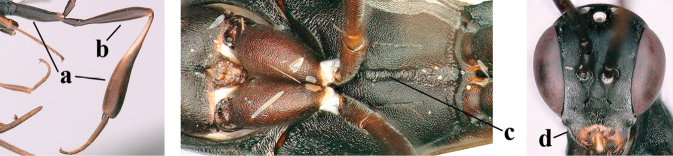		
–	Hind coxa about as wide as hind tibia (aa); hind femur wider medially (bb); mesosternal sulcus wide and coarsely crenulate (cc); mandible brownish-yellow basally (dd)	**52**
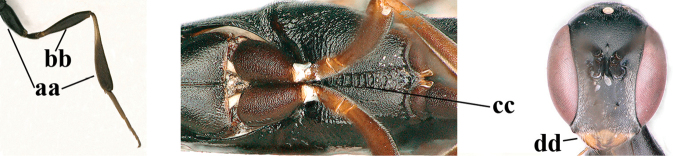		
52	Anterior half of propleuron parallel-sided and long in ventral view (a); propleuron 1.1–1.2× as long as mesoscutum up to tegula in lateral view (b); head comparatively long in dorsal view (c); [eye elongate in lateral view; ♂ unknown]	***G. amoyense* Pasteels, 1958**
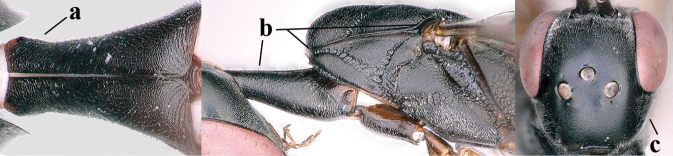		
–	Anterior half of propleuron narrowed anteriorly and rather short in ventral view (aa); propleuron 0.8–0.9× as long as mesoscutum up to tegula in lateral view (bb); head short in dorsal view (cc)	**53**
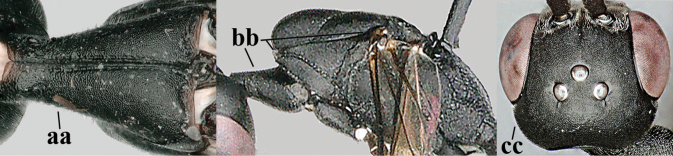		
53	Mesoscutum densely and finely granulate (a); scutellum granulate and rather flat (b); frons granulate and matt (c); [hind tibia slender and yellowish-brown ventro-basally]	***G. granulatum* sp. nov.**
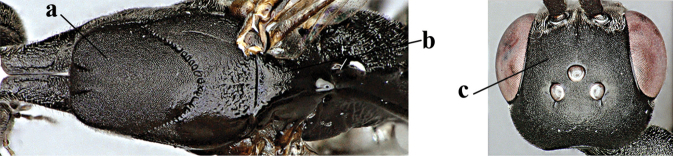		
–	Mesoscutum rugulose or punctate (aa); scutellum rugulose and slightly more convex (bb); frons largely smooth, and more or less shiny (cc); [♂ unknown]	***G. pannuceum* Tan & Achterberg, 2016**
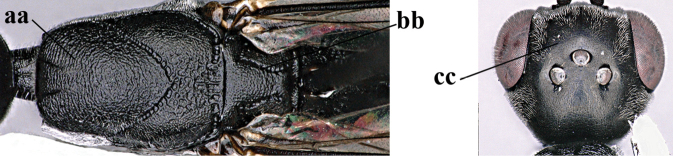		
54	Frons and vertex shiny and smooth (a); mesoscutum rugose medio-posteriorly (b) and near notauli (c); hind femur slightly wider (c); [mesoscutum with satin sheen]	***G. latitibia* Zhao, van Achterberg & Xu, 2012**
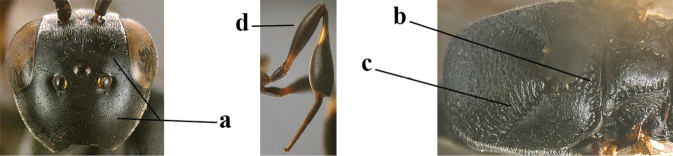		
–	Frons and vertex matt or with satin sheen, micro-sculptured (aa); mesoscutum medio-posteriorly rugulose (bb) or mainly punctate and near notauli coriaceous or rugulose (cc); hind femur usually more slender (dd)	**55**
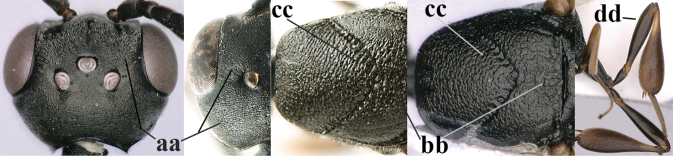		
55	Apex of paramere yellowish-brown (a); pre-apical metasomal sternite distinctly yellowish posteriorly (b); mandible yellowish basally (c)	***G. flavimarginatum* van Achterberg, 2014**
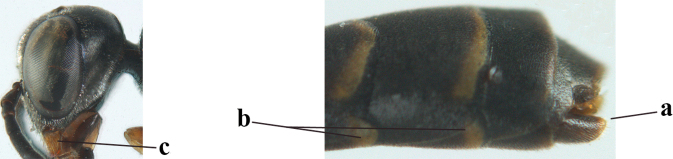		
–	Apex of paramere dark brown (aa); pre-apical metasomal sternite at most narrowly yellowish posteriorly (bb); colour of mandible variable, often darkened basally (cc)	**56**
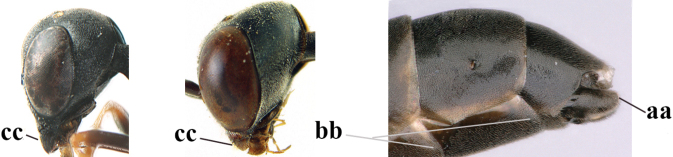		
56	Mandible largely pale yellowish (a)	**57**
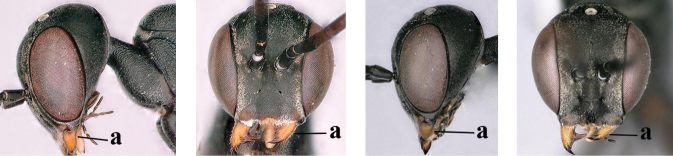		
–	Mandible largely brown, dark brown or black (aa); [additional females needed for reliable identification]	**58**
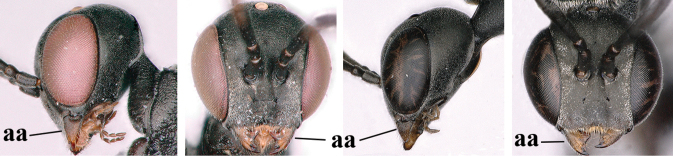		
57	Middle lobe of mesoscutum distinctly protruding (a); head longer and rather trapezoid in dorsal view (b); hind tibia dark brown or blackish ventrally (c); scutellum wider (d)	***G. brevicuspis* Kieffer, 1911**
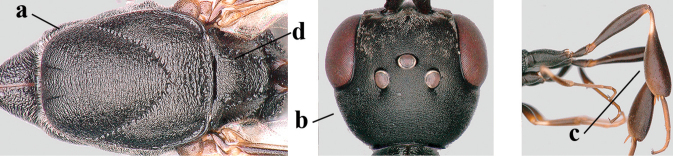		
–	Middle lobe of mesoscutum hardly protruding (aa); head shorter and transverse in dorsal view (bb); hind tibia more or less yellowish-brown ventrally (cc); scutellum narrower (dd)	***G. bicoloratum* Tan & van Achterberg, 2016**
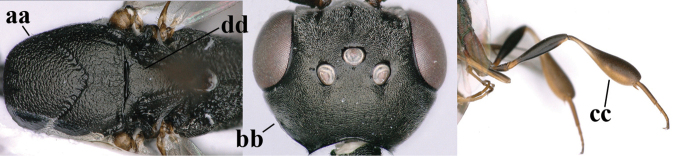		
58	Hind tibia with ill-defined pale subbasal patch (a); hypostomal bridge usually medium-sized (b)	***G. nigritarse* (Thomson, 1883)**
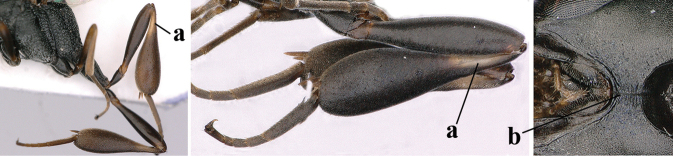		
–	Hind tibia usually with well-defined subbasal ivory patch (aa); hypostomal bridge narrow (bb)	**59**
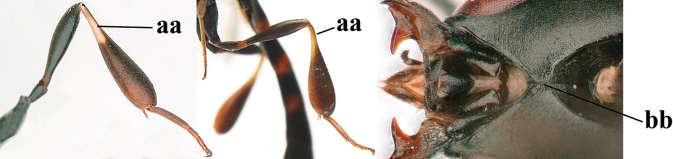		
59	Mesoscutum often coarser sculptured than vertex (a); head directly narrowed behind eyes (b); temple rather convex (c)	***G. assectator* (Linnaeus, 1758)**
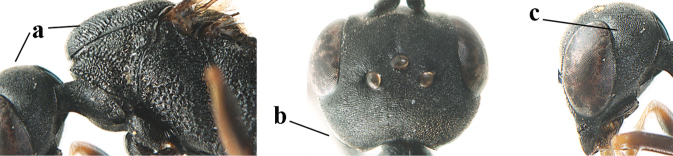		
–	Mesoscutum slightly coarser sculptured than vertex (aa); head less directly narrowed behind eyes (bb); temple less convex (cc)	***G. abeillei* Kieffer, 1912**
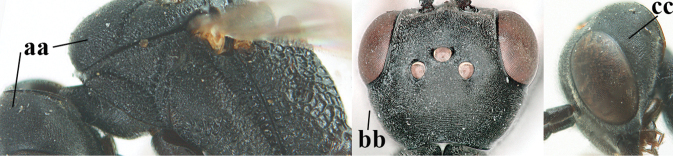		
60	Posteriorly vertex flat in lateral view and long (a); head smooth and shiny dorsally (b); head moderately narrowed posteriorly (c); mesosoma laterally often paler than dorsally (d)	***G. bimaculatum* Pasteels, 1958**
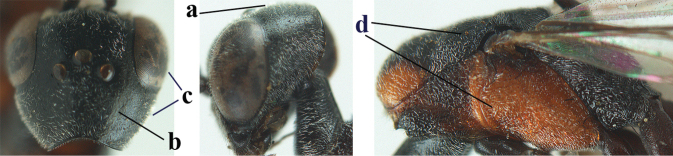		
–	Posteriorly vertex convex in lateral view and usually shorter (aa); sculpture of head dorsally variable (bb); if smooth (bbb), then more narrowed posteriorly (cc); mesosoma usually unicoloured (dd) or dorsally paler than laterally	**61**
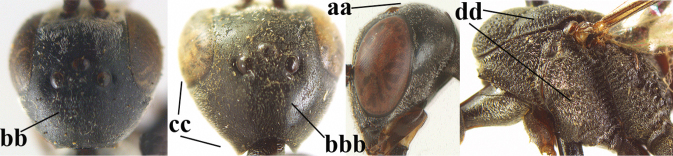		
61	Hind coxa orange brown or dark brown (a); mesoscutum conspicuously setose (b); metasoma largely reddish-brown (c); [mandible orange yellow or yellowish-brown; mesoscutum finely or coarsely punctate and often with narrow interspaces]	**62**
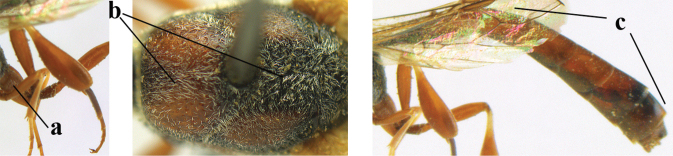		
–	Hind coxa black (aa); mesoscutum less conspicuously setose (bb); metasoma often largely dark brown or black (cc)	**64**
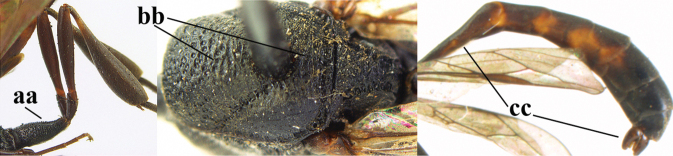		
62	Mesoscutum finely punctate, with rather wide interspaces (a); hind tibia yellowish-brown or indistinctly infuscate basally (b); apical half of hind basitarsus partly dark brown and only apically ivory (c)	***G. dilutum* Semenov, 1892**
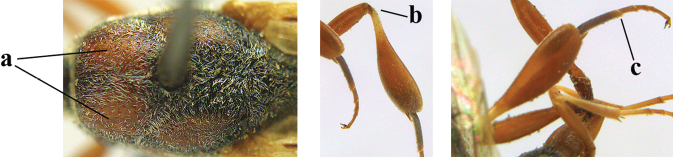		
–	Mesoscutum coarsely punctate and interspaces narrower (aa); hind tibia distinctly dark brown basally (bb); apical half of hind basitarsus mainly ivory (cc)	**63**
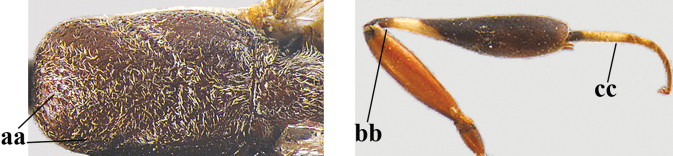		
63	Basal half of hind coxa rugose dorsally (a); outer side of hind tibia (except basally) dark brown or blackish (b); head subtruncate posteriorly in dorsal view (c)	***G. coloratum* Zhao, van Achterberg & Xu, 2012**
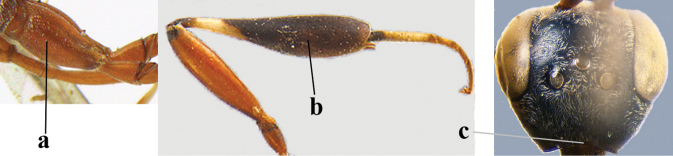		
–	Basal half of hind coxa superficially coriaceous dorsally (aa); ventral half of outer side of hind tibia orange-brown (bb); head emarginate posteriorly in dorsal view (cc)	***G. argentifrons* Semenov-T.-S. & Kostylev, 1928**
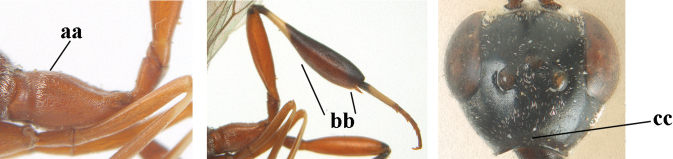		
64	Third antennal segment slightly longer than wide and similar to pedicellus (a); ventral half of pronotal side largely superficially coriaceous to nearly smooth, only grooves crenulate (b); pronotum partly densely setose (c)	**65**
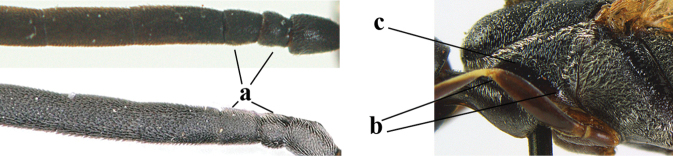		
–	Third antennal segment less robust compared to pedicellus and distinctly longer than wide (aa); ventral half of pronotal side largely moderately reticulate-rugose, at most ventrally coriaceous (bb); pronotum sparsely setose (cc)	**66**
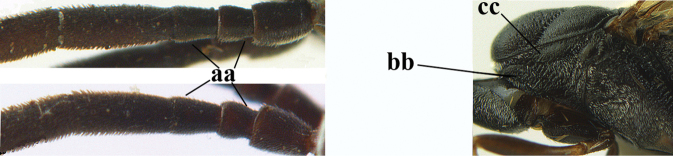		
65	Malar space narrow (a); head less emarginate medio-posteriorly (b); mesoscutum usually with less coarse sculpture (c); scutellum mainly micro-sculptured (d), at most with few large punctures	***G. shengi* Tan & van Achterberg, 2016**
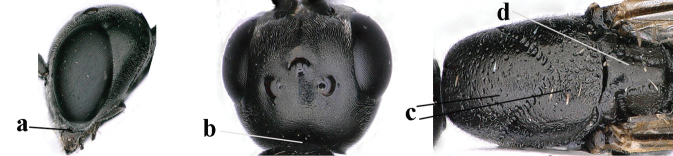		
–	Malar space slightly wider (aa); head distinctly emarginate medio-posteriorly (bb); mesoscutum with coarser sculpture (cc); scutellum coarsely punctate (dd)	***G. dimidiatum* Semenov, 1892**
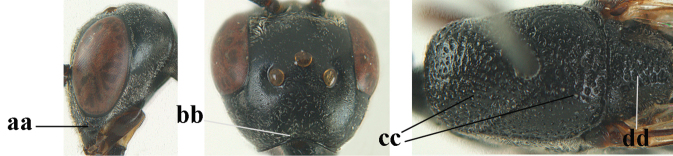		
66	Hind tibia distinctly inflated and bulging ventrally (a); head directly narrowed posteriorly in dorsal view (b); third antennal segment slender, 1.6–1.9× as long as second segment (c); [mesoscutum distinctly “crater-like” punctate; head concave medio-posteriorly]	**67**
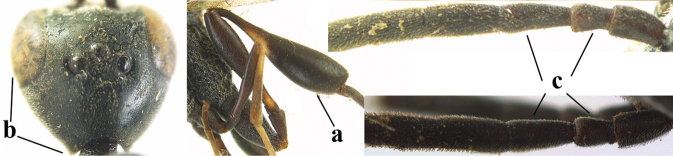		
–	Hind tibia slender and hardly bulging ventrally (aa); head usually gradually narrowed posteriorly in dorsal view (bb); third antennal segment robust, 1.2–1.7× as long as second segment (cc)	**68**
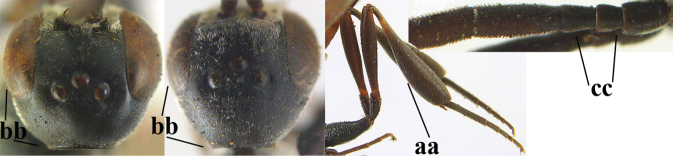		
67	Hind tibia strongly inflated (a); third antennal segment slightly more robust (b)	***G. sinicola* (Kieffer, 1924)**
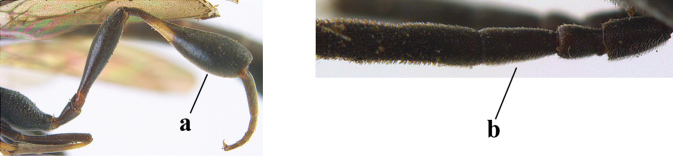		
–	Hind tibia less inflated (aa); third antennal segment slender (bb)	***G. formosanum* Enderlein, 1913**
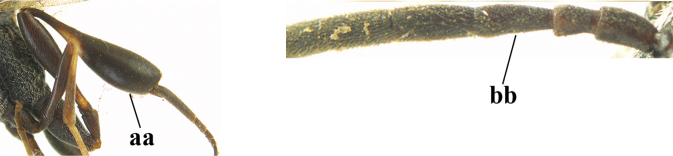		
68	Occipital carina moderately wide (a); vertex distinctly convex (b); [middle lobe of mesoscutum coarsely punctate laterally]	***G. tonkinense* Pasteels, 1958**
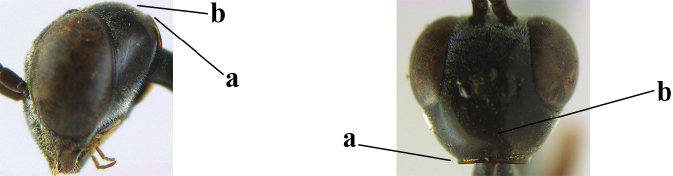		
–	Occipital carina narrow lamelliform or non-lamelliform (aa); vertex comparatively flat (bb)	**69**
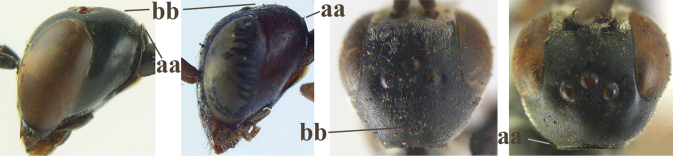		
69	Occipital carina narrow lamelliform (a) and vertex slightly depressed medio-dorsally (b); mesoscutum less coarsely punctate (c)	***G. japonicum* Cameron, 1888**
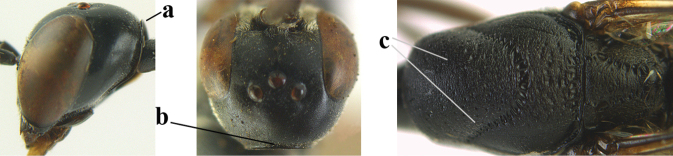		
–	Occipital carina less or non-lamelliform (aa) and vertex flat medio-dorsally (bb); mesoscutum more or less coarsely punctate (cc)	**70**
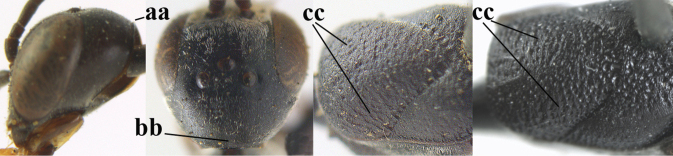		
70	Lateral lobe of mesoscutum rather matt and at most finely punctate (a); head slightly enlarged below eyes in anterior view (b)	***G. subtile* (Thomson, 1883)**
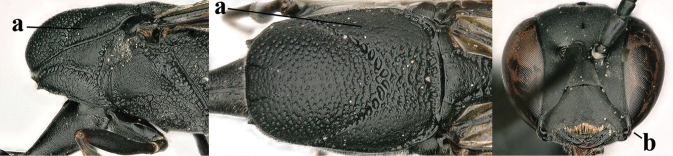		
–	Lateral lobe of mesoscutum with satin sheen and with coarse punctures (aa); head not enlarged below eyes in anterior view (bb)	***G. sinarum* Kieffer, 1911**
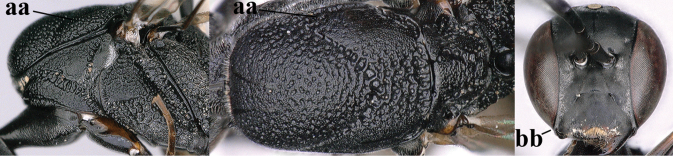		

##### Systematics

### Gasteruption abeillei

Taxon classificationAnimaliaHymenopteraGasteruptiidae

Kieffer, 1912

5A02ABD8-037F-5EA0-A9D4-61B71A07EA2F

Figs 1–2 , 3 , 4–12

Gasteruption abeillei Kieffer, 1912: 228, 231, 251; Hedicke 1939: 5; Ferrière 1946: 235, 240; Leclercq 1948: 75; Wall 1994: 148; van Achterberg et al. 2019: 3 (as valid species). Synonymised with G. assectator (Linnaeus) by Madl 1989a.
Trichofoenus breviterebrae Watanabe, 1934: 285; Hedicke 1939: 45. Synonymised with G. assectator (Linnaeus) by Pagliano and Scaramozzino (2000) and with G. boreale (Thomson) by Tan et al. (2016) and Johansson and van Achterberg (2016). Synonymised with G. abeillei by van Achterberg et al. 2019.

#### Additional material.

3 ♀ + 3 ♂ (NWUX, RMNH), “NW China: Shaanxi, Huaishuzhuang Rev. St., Ziwuling NNR, Fuxian, Yanan, sweep net, 35.86°N, 108.74°E, 4.viii.2019, 1271 m alt. Jiangli Tan, NWUX”.

**Figures 1, 2.**
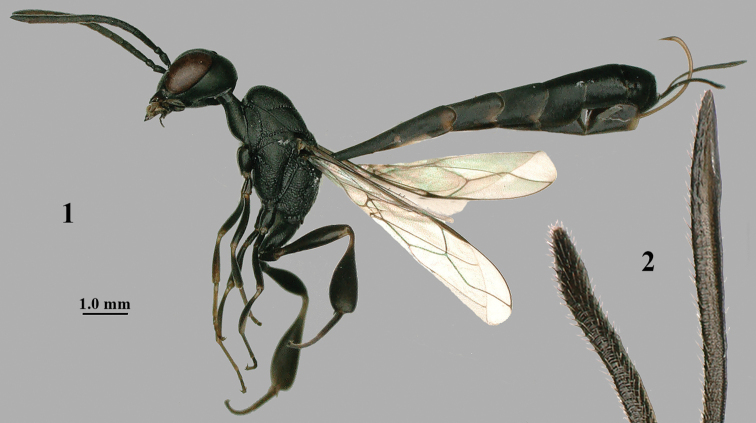
*Gasteruption abeillei* Kieffer, female, Shaanxi **1** habitus lateral **2** detailed apical half of ovipositor sheath lateral.

#### Distribution.

China (Shaanxi), Russia (Far East), Europe. New for Shaanxi.

**Figure 3.**
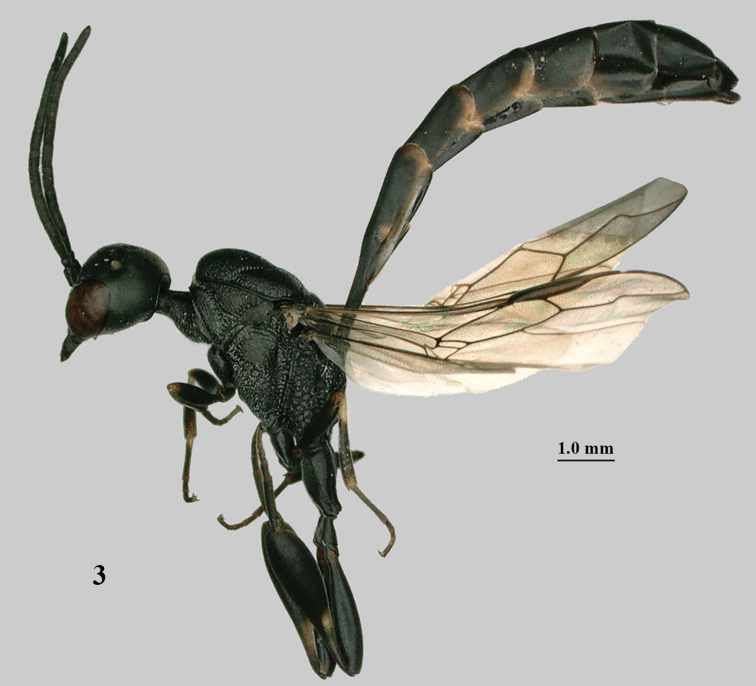
*Gasteruption abeillei* Kieffer, male, Shaanxi, habitus lateral.

**Figures 4–12.**
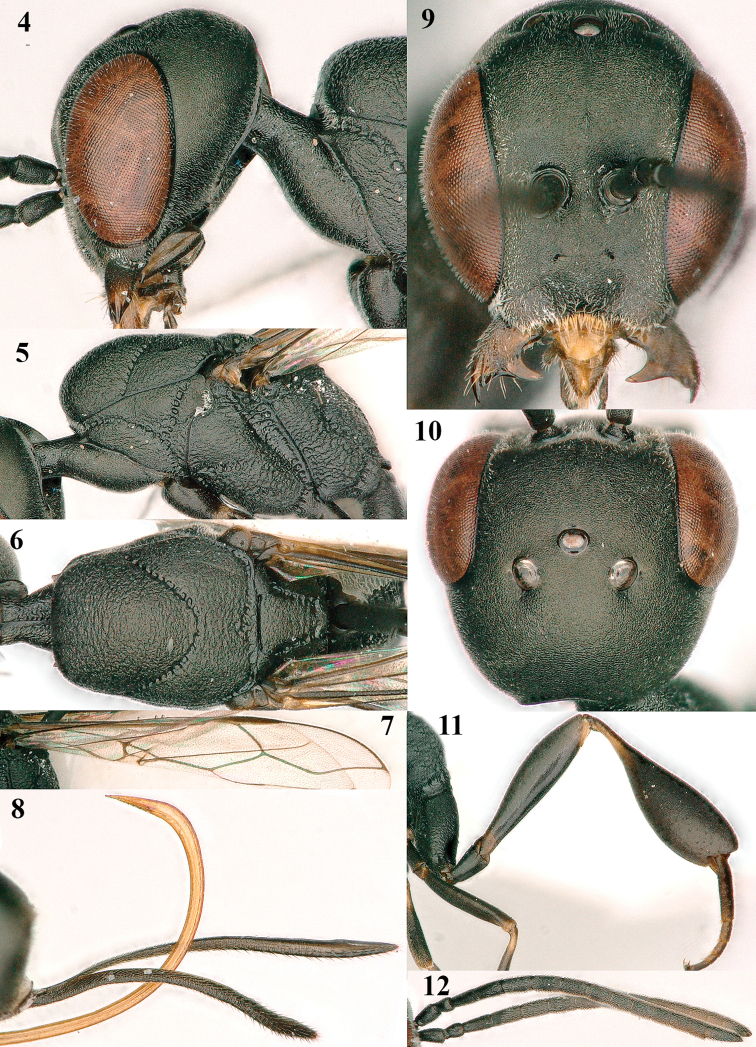
*Gasteruption abeillei* Kieffer, female, Shaanxi **4** head lateral **5** mesosoma lateral **6** mesosoma dorsal **7** wings **8** ovipositor and its sheath lateral **9** head anterior **10** head dorsal **11** hind leg **12** antenna.

### Gasteruption amoyense

Taxon classificationAnimaliaHymenopteraGasteruptiidae

Pasteels, 1958

DD50CA5A-31C0-569E-B8FE-2056E8A0457C

Figs 13–17 , 18–26

Gasteruption amoyense Pasteels, 1958: 178–179; Zhao et al. 2012: 17–19.
Gasteruption curiosum Pasteels, 1958: 177–178; Zhao et al. 2012: 17 (synonymised with G. amoyense Pasteels, 1958).

#### Additional material.

1 ♀ (NWUX), “NW China: Shaanxi, Taibai Mt., Haopingsi, Meixian [= part of Baoji], Shangbaiyun, 4.viii.2017, Jiangli Tan, NWUX”; 1 ♀ (RMNH), “NW China: Shaanxi, Xunyangba, Ningshaan, 33.55°N 108.55°E, 16.viii.2016, 1481 m alt., JL Tan & T Zhou, NWUX”; 1 ♀ (NWUX), “S China: Fujian, Huboliao NNR, Letu, 24.54°N 117.12°E, Mal. trap, viii.2015, alt. 300 m alt., Qingqing Tan, NWUX”.

**Figures 13–17.**
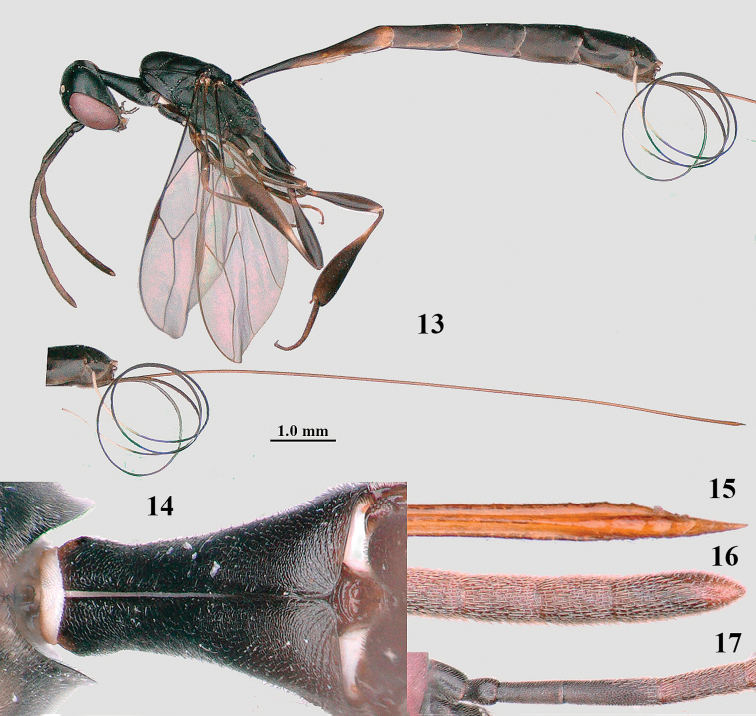
*Gasteruption amoyense* Pasteels, female, Shaanxi **13** habitus lateral **14** propleuron ventral **15** apex of ovipositor **16** apex of antenna **17** base of antenna.

#### Notes.

Similar to the West Palaearctic *G. syriacum* Szépligeti, 1903, because of the slender pronotum (Fig. 19), the elongate head (Fig. 25), the distinctly inflated hind tibia (Fig. 26) and the yellowish or orange mandibles, but differs mainly by the very fine granulate-coriaceous sculpture of the mesoscutum with only sparse (and often shallow) punctures, in addition to smaller ocelli. *Gasteruption syriacum* has the mesoscutum coarser punctate and the interspaces are only finely punctulate or somewhat coriaceous and the ocelli are large (van Achterberg and Talebi 2014).

**Figures 18–26.**
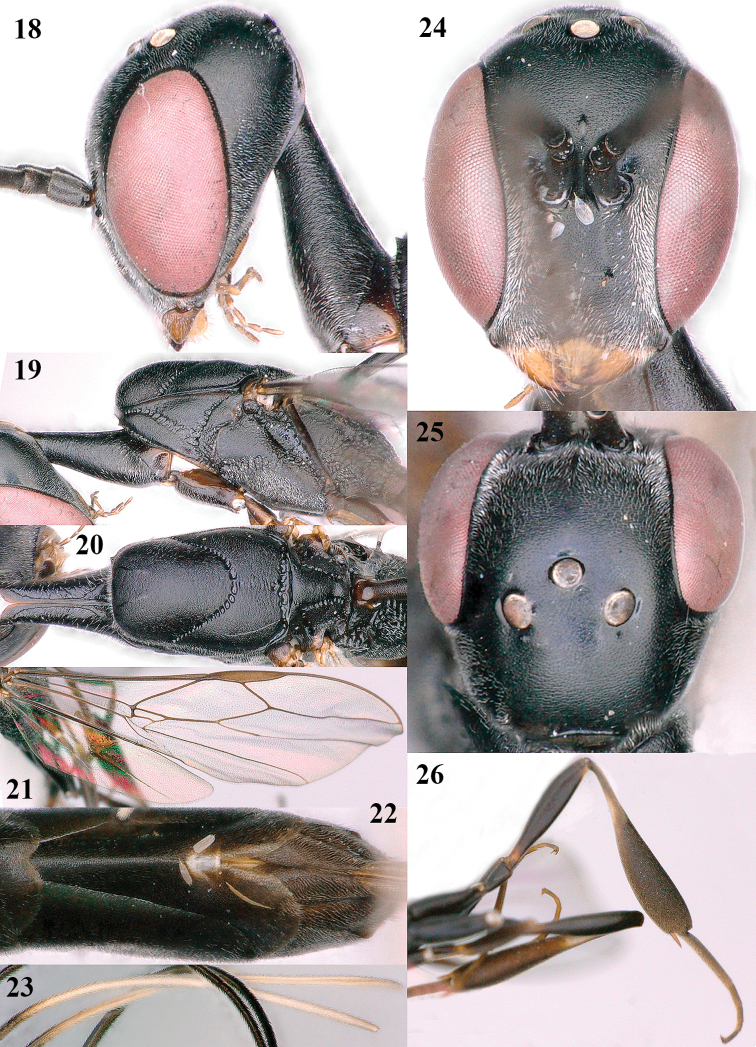
*Gasteruption amoyense* Pasteels, female, Shaanxi **18** head lateral **19** mesosoma lateral **20** mesosoma dorsal **21** wings **22** apex of metasoma ventral **23** apex of ovipositor sheath lateral **24** head anterior **25** head dorsal **26** hind leg lateral.

#### Distribution.

China (Fujian, Hong Kong, Hunan, Shaanxi, Zhejiang). New for Shaanxi and Fujian; Shaanxi is the first record from the Palaearctic Region.

### Gasteruption angulatum

Taxon classificationAnimaliaHymenopteraGasteruptiidae

Zhao, van Achterberg & Xu, 2012

74602893-BD9E-5C1D-B8A8-96442365CB58

Figs 27–28 , 29–37

Gasteruption angulatum Zhao et al., 2012: 19–22 (description).

#### Additional material.

1 ♂ (NWUX), “NW China: Shaanxi, Xunyangba, Ningshaan, 33.55°N, 108.55E°, 20.v-23.vi.2016, g[reen] Malaise trap, 1481 m alt., JL Tan & QQ Tan, NWUX”.

#### Distribution.

China (Henan, Hubei, Shaanxi, Zhejiang).

**Figures 27–28.**
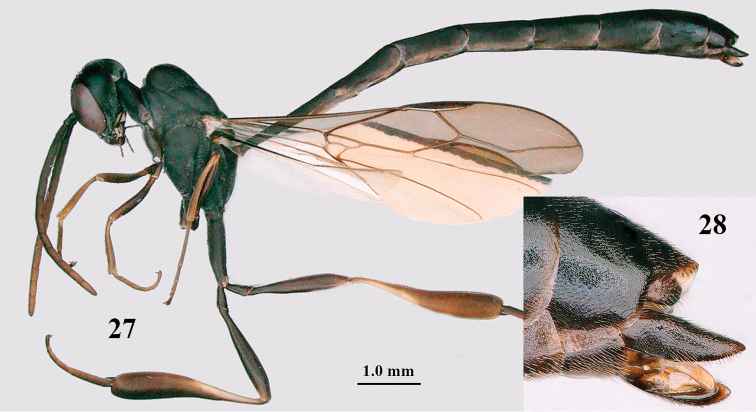
*Gasteruption angulatum* Zhao, van Achterberg & Xu, male, Shaanxi **27** habitus lateral **28** apex of metasoma lateral.

#### Notes.

The male from Baolongyu (Qinling Mts, Shaanxi) illustrated by Tan et al. (2016) is the male of *G. granulatum* sp. nov. It differs from typical *G. angulatum* males because the middle lobe of the mesoscutum is less protruding and very regularly granulate (Figs 118 and 119) and the hind coxa is somewhat less slender (Fig. 120) than normal for *G. angulatum*. The male, listed above, was collected later and agrees better with the type series. This male has the middle lobe of the mesoscutum distinctly protuberant (Fig. 30 as in fig. 20 in Zhao et al. 2012) and very slender hind coxa (Figs 27 and 36, as in fig. 23 l.c.).

**Figures 29–37.**
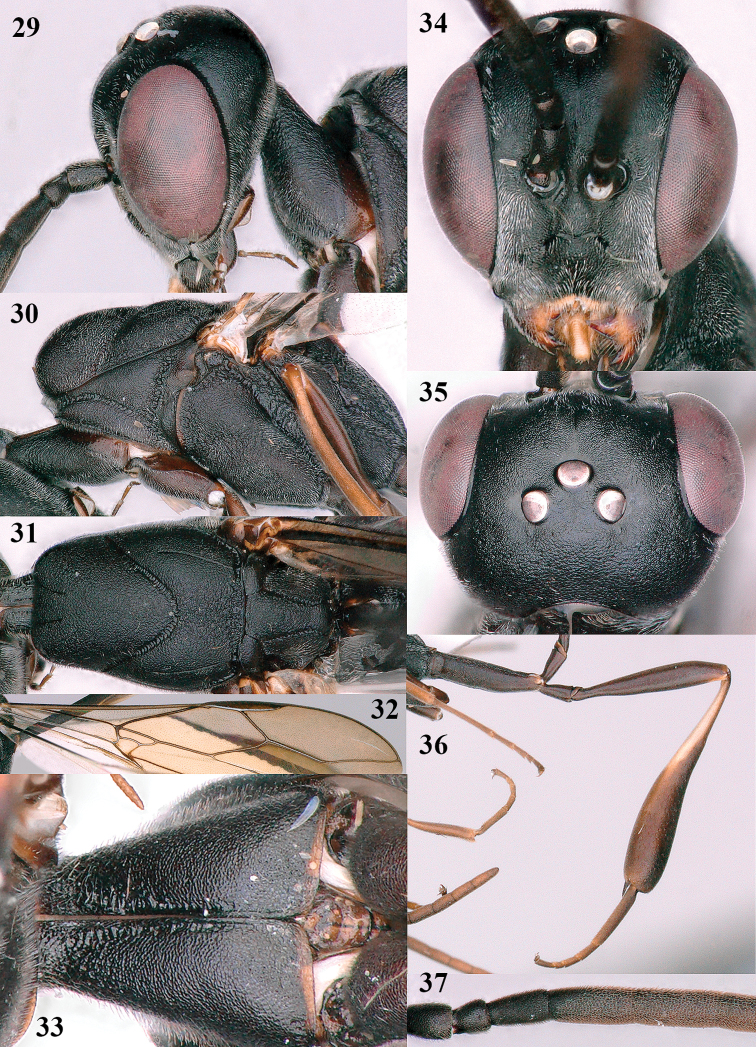
*Gasteruption angulatum* Zhao, van Achterberg & Xu, male, Shaanxi **29** head lateral **30** mesosoma lateral **31** mesosoma dorsal **32** wings **33** propleuron ventral **34** head anterior **35** head dorsal **36** hind leg **37** base of antenna.

### Gasteruption assectator

Taxon classificationAnimaliaHymenopteraGasteruptiidae

(Linnaeus, 1758)

B31E0B18-A6E8-59D0-B2BF-2EFB7DC2BAB1

Ichneumon assectator Linnaeus, 1758: 566.
Gasteruption assectator ; Zhao et al. 2012: 24.
Gasteruption margotae Madl, 1987: 225–227. Synonymised with G. assectator (Linnaeus) by Madl (1990b) and with G. boreale (Thomson) by Johansson and van Achterberg (2016).

#### Distribution.

China (Heilongjiang; Hubei (1800 m alt.), Jilin (740 m alt.), Shanxi (1148–1700 m alt.), Qinghai (3300–4288 m alt.), Tibet (2800 m alt.), Xinjiang (1148–2425 m alt.), Russia (Far East), Europe.

#### Notes.

The interpretation of the very similar *Gasteruption boreale* (Thomson, 1883) is problematic because the lectotype is a male and the most reliable differences are found in the setosity of the ovipositor sheath of the female. The somewhat elongated malar space indicates that it belongs to a species of the *G. assectator* aggregate with entirely adpressed setosity of the ovipositor sheath and the ovipositor sheath is usually somewhat longer than the hind tibia. So far, no females of typical *G. boreale* are known from China. *Gasteruption assectator* is easily confused with *G. latitibia* Zhao, van Achterberg & Xu, 2012; if the head is distinctly convex dorsally and minute pronotal teeth are present, the specimen most likely belongs to the latter species.

### Gasteruption bicoloratum

Taxon classificationAnimaliaHymenopteraGasteruptiidae

Tan & van Achterberg, 2016

DFE521C1-5363-53EC-8652-A021490AAA04

Gasteruption bicoloratum Tan & van Achterberg, 2016: 86–90; van Achterberg et al. 2019: 4.

#### Additional material.

1 ♀ (NWUX), “NW China: Shaanxi, Luonan, Yunmeng Mt., 34.08°N 110.02°E, 10.vii.2017, alt. 1084 m, Ruonan Zhang, NWUX”; 1 ♂ (NWUX), “NW China: Gansu, Lianjiabian, Taibai, Heshui, sweep net, 36.06°N, 108.54°E, 8.viii.2019, alt. 1193 m, Ruonan Zhang, NWUX”.

#### Distribution.

China (Gansu, Shaanxi), Russia (Far East). New for Gansu.

### Gasteruption bimaculatum

Taxon classificationAnimaliaHymenopteraGasteruptiidae

Pasteels, 1958

779972D1-C2B7-5EBC-A908-3E217BEC1727

Figs 38–40 , 41–49 , 50–56

Gasteruption bimaculatum Pasteels, 1958: 191–192 (only holotype ♂); Zhao et al. 2012: 30–35.
Gasteruption obscuripenne Pasteels, 1958: 189–190 (p.p.).

#### Additional material.

1 ♀ + 1 ♂ (NWUX), “NW China: Shaanxi, Bailuyuan, Baqiao, Xi’an, 34.20°N, 109.12°E, 14.vii.2018, 687 m alt., Ruonan Zhang, NWUX”; 1 ♂ (NWUX), NW China: Shaanxi, South campus of NWU, Xi’an, 34.14°N, 108.87°E, 30.vi.2020, 408 m alt., sweep net, Jiangli Tan, NWUX; 1 ♀ (NWUX), “SW China, Yunnan, Mango Forest Park, Yuanjiang, Yuxi, 23.56°N, 101.99°E, 26.vii.2018, 466 m alt., JL Tan & QQ Tan, NWU”; 1 ♀ (NWUX), “S China: Fujian, Huboljiao, Nanping, 24°54'24"N 117°12'52"E, 17.v.2018, Mal. trap, 300 m alt., Lingfei Peng, NWUX”; 1 ♀ (RMNH), id., 28.v.2018.

**Figures 38–40.**
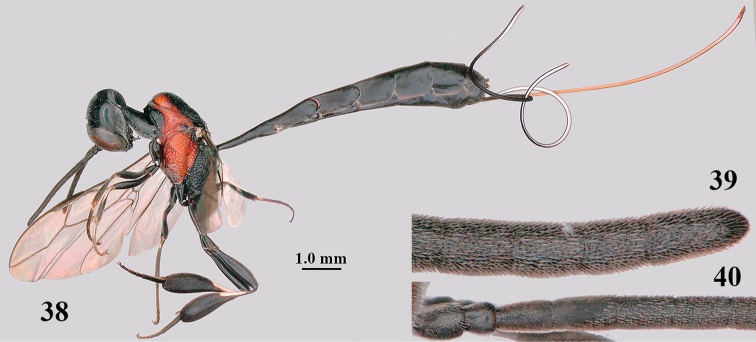
*Gasteruption bimaculatum* Pasteels, female, Shaanxi, habitus lateral.

#### Distribution.

China (Fujian, Guangxi, Hainan, Henan, Shaanxi, Tibet, Yunnan); Burma. New for Shaanxi and its most northern record.

**Figures 41–49.**
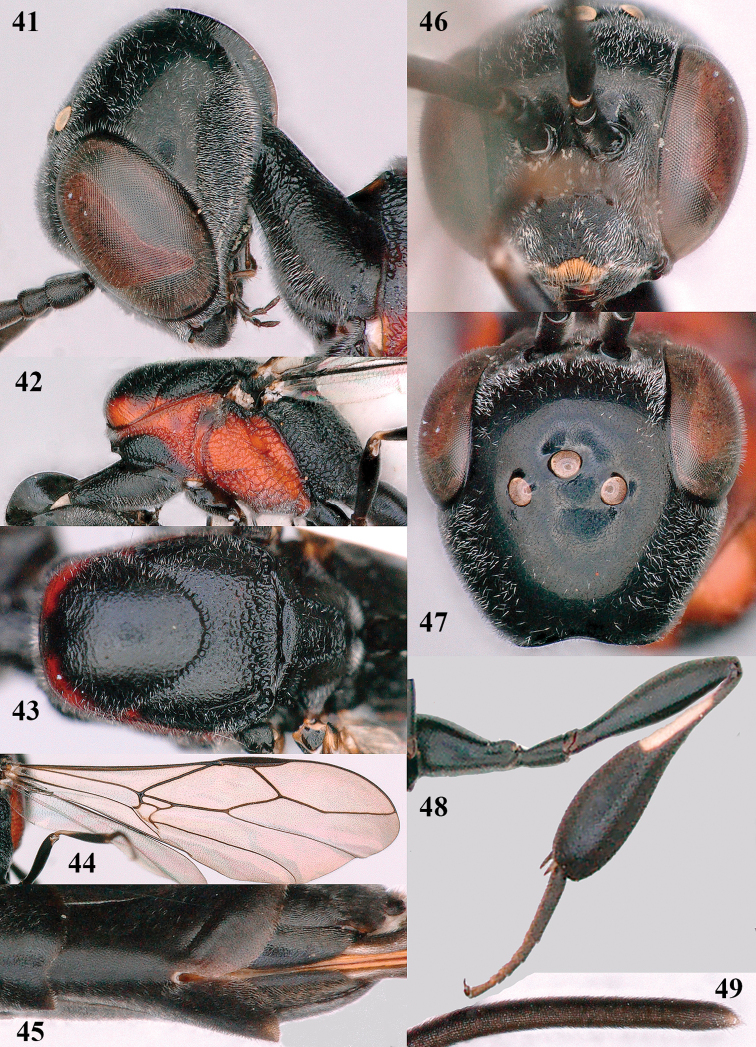
*Gasteruption bimaculatum* Pasteels, female, Shaanxi **41** head lateral **42** mesosoma lateral **43** mesosoma dorsal **44** wings **45** apex of metasoma ventral **46** head anterior **47** head dorsal **48** hind leg **49** apex of ovipositor sheath lateral.

#### Notes.

The specimens from Shaanxi have the sculpture of the mesoscutum reduced, especially in the female (Fig. 43).

**Figures 50–56.**
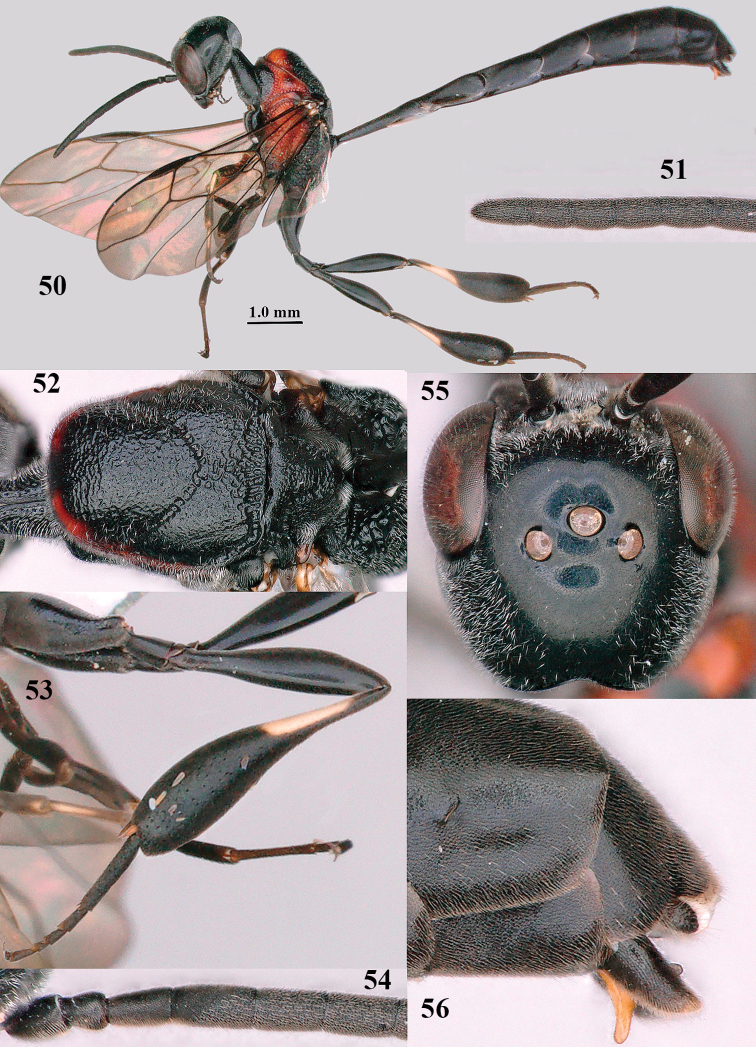
*Gasteruption bimaculatum* Pasteels, male, Shaanxi **50** habitus lateral **51** apex of antenna **52** mesosoma dorsal **53** hind leg **54** base of antenna **55** head dorsal **56** apex of metasoma lateral.

### Gasteruption brevicuspis

Taxon classificationAnimaliaHymenopteraGasteruptiidae

Kieffer, 1911

6DC847E7-23BC-5B81-A262-11781C8DDC43

Figs 57–62 , 63–71 , 72–79

Gasteruption brevicuspis Kieffer, 1911: 196, 1912: 293–294; van Achterberg et al. 2019: 4.
Gasteruption terebrelligerum Enderlein, 1913: 324; Hedicke 1939: 28; Pasteels 1958: 189; Zhao et al. 2014: 97–100; van Achterberg 2019a: 9 (as synonym of G. brevicuspis Kieffer), 2019b: 21 (id.).

#### Additional material.

3 ♀ + 5 ♂ (NWUX) Shaanxi, Lantian Ape Man Site, 34.10°N, 110.30°E, 8.vii.2017, 775 m alt., Qingqing Tan; 1 ♀ + 1 ♂ (RMNH), Shaanxi, Qinling Mts., nr Lantian Man Museum, 34°11'N, 109°29'E, 8–9.vii.2017, 735 m alt., C. v. Achterberg; 2 ♀ (RMNH), Shaanxi, Qinling Mts., Luonan, Maping, Yunmeng Mt., 9–10.vii.2017, ca. 1090 m alt., 34°8'N, 110°7'E, C. v. Achterberg; 6 ♀ (NWUX), id., but 34.08°N, 110.02°E, 1084 m alt., Ruonan Zhang/Qingqing Tan/Jiangli Tan; 1 ♀ (NWUX), NE China: Shaanxi, Xi’an, NWU Taibai campus, small garden, ca. 410 m alt., 3.ix.2018, JL Tan; 1 ♀ (RMNH), “NW China: Shaanxi, Bailuyuan, Baqiao, Xi’an, 34.20°N, 109.12°E, 14.vii.2018, 687 m alt., Ruonan Zhang, NWUX”; 1 ♀ (NWUX), “NW China: Shaanxi, Northwest University, 34.14°N, 108.55°E, 3.ix.2018, 365 m alt., Qing-qing Tan, NWUX”; 1 ♀ + 3 ♂ (NWUX), id., but 13/29.vi.2020, North Campus, Jiang-Li Tan; 15 ♀ + 6 ♂ (NWUX), id., but 30.vi.2020, North Campus, Jiang-Li Tan; 1 ♀ (NWUX), “NW China: Shaanxi, Jiufeng, Zhouzhi, 459 m alt., 34°08'N, 108°42'E, 16.vi.2019, Tan JL, NWUX”; 1 ♂ (NWUX), “NW China: Shaanxi, Huaishuzhuang Rev. St., Ziwuling NNR, Fuxian, Yanan, sweep net, 35.86°N, 108.74°E, 2.viii.2019, alt. 1127 m, Jiangli Tan, NWUX”; 2 ♀ + 1 ♂ (NWUX, RMNH), “NW China: Gansu, Lianjiabian, Taibai Heshui, sweep net, 36.06°N, 108.54°E, 8.viii.2019, alt. 1193 m, Ruonan Zhang, NWUX”.

**Figures 57–62.**
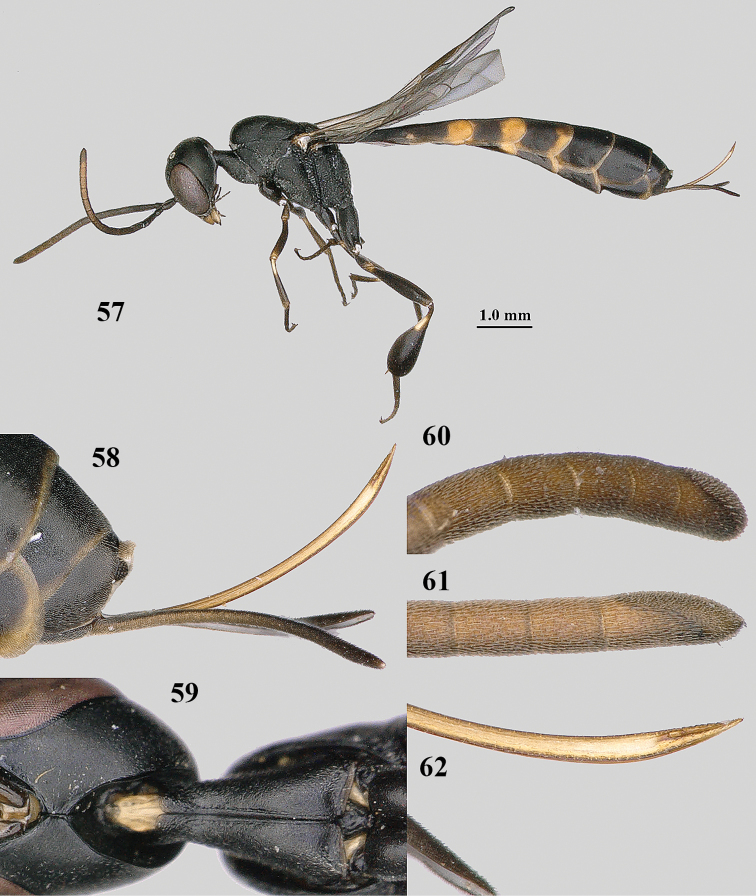
*Gasteruption brevicuspis* Kieffer, female, Shaanxi **57** habitus lateral **58** ovipositor and its sheath **59** propleuron ventral **60** apex of antenna lateral **61** apex of antenna latero-ventral **62** ovipositor apex lateral.

#### Notes.

This species is similar to *G. assectator* (Linnaeus), but differs by having a slightly longer head in dorsal view, the vertex more convex in lateral view, the hind tibia more slender, the pronotal teeth more or less developed and the mandibles brownish-yellow or yellow (dark brown in *G. assectator*). Closely related to *G. bicoloratum* Tan and van Achterberg and differs mainly by the shape of the head as illustrated in the key.

**Figures 63–71.**
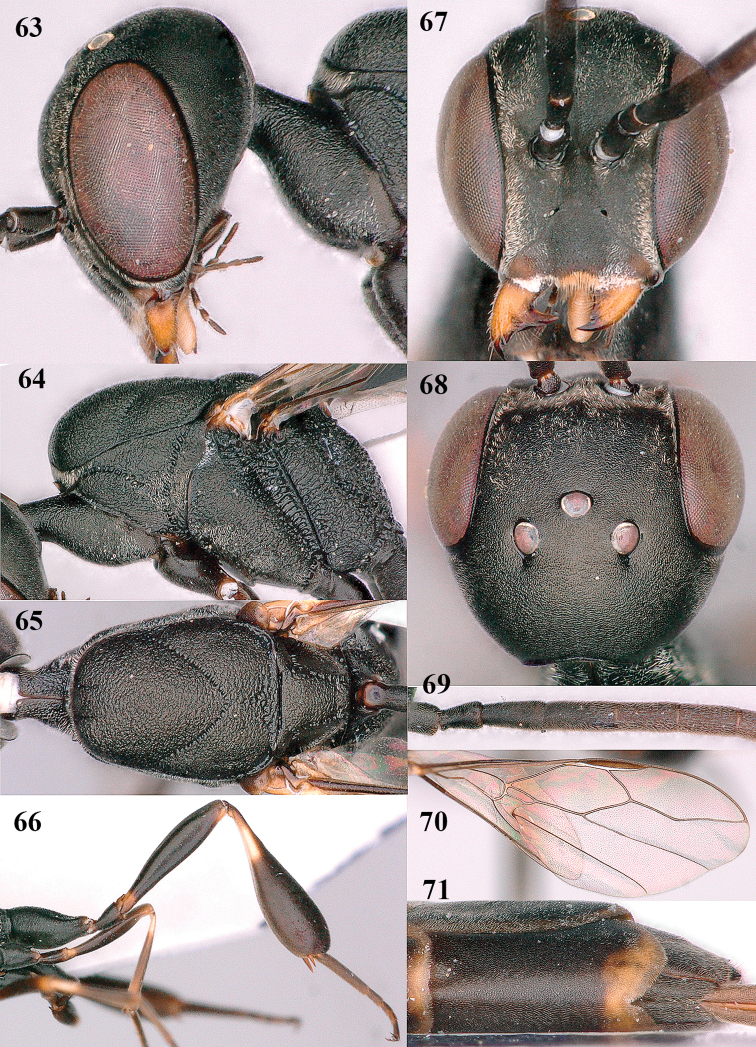
*Gasteruption brevicuspis* Kieffer, female, Shaanxi **63** head lateral **64** mesosoma lateral **65** mesosoma dorsal **66** hind leg **67** head anterior **68** head dorsal **69** base of antenna lateral **70** fore wing **71** apex of metasoma ventral.

#### Distribution.

China (Fujian, Gansu, Hubei, Hunan, Shaanxi, Shanxi, Taiwan, Yunnan), India, Myanmar, Russia (Far East). New for Gansu and Shaanxi.

**Figures 72–79.**
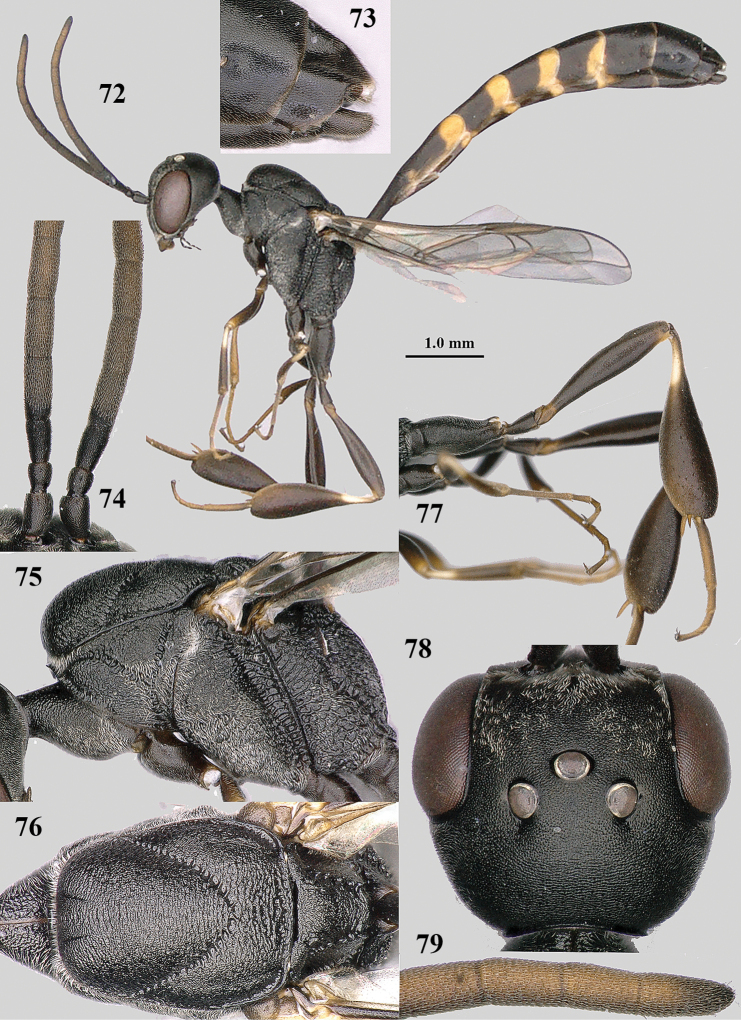
*Gasteruption brevicuspis* Kieffer, male, Shaanxi **72** habitus lateral **73** apex of metasoma lateral **74** base of antennae **75** mesosoma lateral **76** mesosoma dorsal **77** hind legs **78** head dorsal **79** apex of antenna.

### Gasteruption coloratum

Taxon classificationAnimaliaHymenopteraGasteruptiidae

Zhao, van Achterberg & Xu, 2012

78ECDBAB-E721-59C6-8E96-A0BB03B2F641

Figs 80–82 , 83–92

Gasteruption coloratum Zhao, van Achterberg & Xu, 2012: 38–40.

#### Additional material.

1 ♀ (NWUX), NW China: Xinjiang, Nanshan, 25.vii.1983; 1 ♀ (NWUX), Xinjiang, Qitai, 25.viii. [198?], Xin-wang Wu.

**Figures 80–82.**
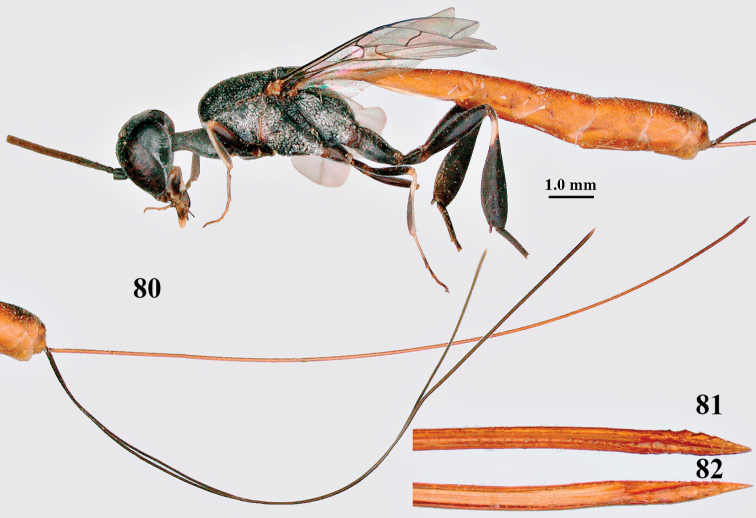
*Gasteruption coloratum* Zhao, van Achterberg & Xu, female, Xinjiang **80** habitus lateral **81** apex of ovipositor lateral **82** id., ventral.

#### Distribution.

China (Xinjiang).

#### Notes.

Both specimens reported here are darker than the holotype (and, so far, only known specimen) of *G. coloratum*, especially the hind leg and the scapus (Zhao et al. 2012). The hind coxa is rather superficially transversely rugose, ovipositor sheath 6–7× as long as hind tibia and its apex dark brown or blackish.

**Figures 83–92.**
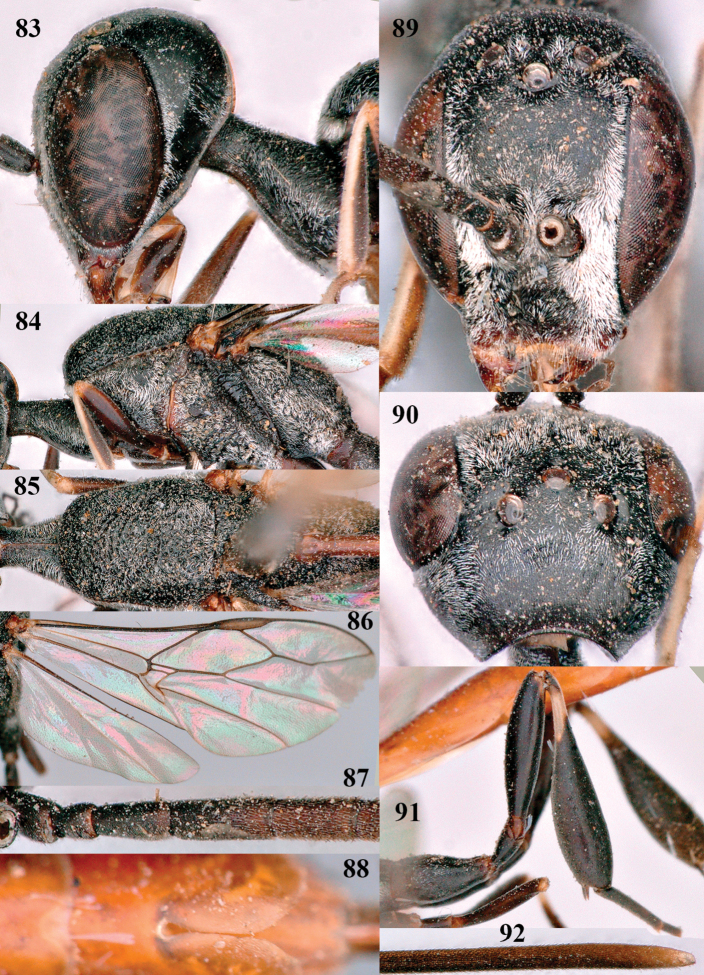
*Gasteruption coloratum* Zhao, van Achterberg & Xu, female, Xinjiang **83** head lateral **84** mesosoma lateral **85** mesosoma dorsal **86** wings **87** base of antenna lateral **88** apex of metasoma ventral **89** head anterior **90** head dorsal **91** hind leg **92** apex of ovipositor sheath.

### Gasteruption corniculigerum

Taxon classificationAnimaliaHymenopteraGasteruptiidae

Enderlein, 1913

D69AACEB-FE27-5E70-AFC8-8863246A18BB

Figs 93–97 , 98–106

Gasteruption corniculigerum Enderlein, 1913: 322–323; Hedicke 1939: 27; Pasteels 1958: 176–177; Zhao et al. 2012: 40–45 (lectotype designation).

#### Additional material.

1 ♀ (NWUX), “NW China: Shaanxi, Haopingsi, Meixian, Baoji, Taibai Mt., swept, 34.04°N, 107.46°E, 15.vii.2017, 1251 m alt., T. Zhou & YX Wu, NWUX”; 1 ♀ (NWUX), “NW China: Shaanxi, Huanghualing, Zhashui, 33.80°N, 108.88°E, 17.viii.2016, 1408 m alt., Jiangli Tan, NWUX”; 1 ♀ (RMNH), “NW China: Shaanxi, [Daba Mts], Hanzhong, Ningqiang, [along road] Huoshizi to Bashan, 1638 m alt., 32.45°N, 106.30°E, [on *Cayratia japonica*], 23.vii.2017, Qing-qing Tan”.

**Figures 93–97.**
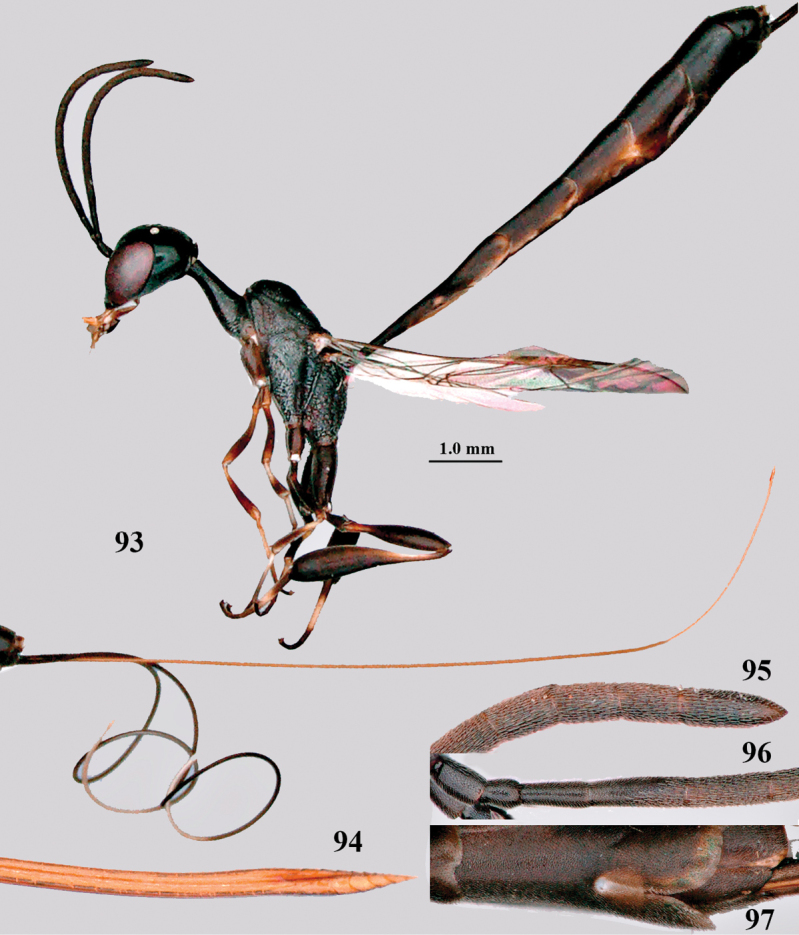
*Gasteruption corniculigerum* Enderlein, female, Shaanxi **93** habitus lateral **94** apex of ovipositor lateral **95** apex of antenna **96** base of antenna **97** apex of metasoma ventral.

#### Distribution.

China (Fujian, Guangdong, Guangxi, Guizhou, Hainan, Hunan, Shaanxi, Taiwan, Zhejiang). New for Shaanxi and for the Palaearctic Region.

**Figures 98–106.**
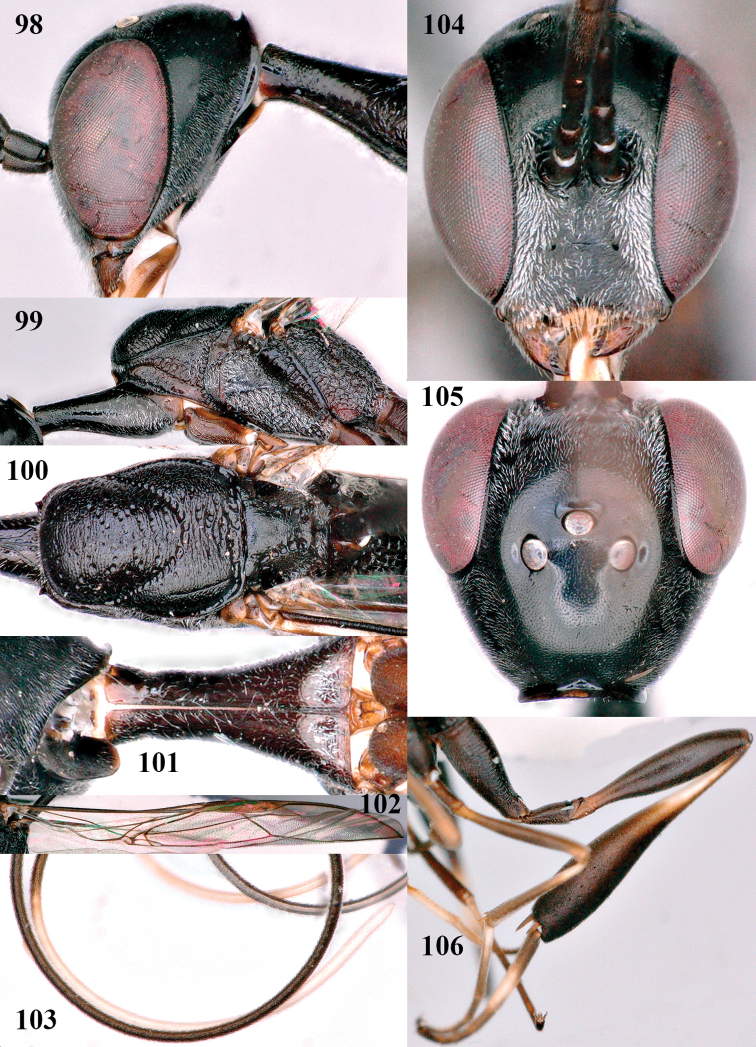
*Gasteruption corniculigerum* Enderlein, female, Shaanxi **98** head lateral **99** mesosoma lateral **100** mesosoma dorsal **101** propleuron ventral **102** fore wing **103** apex of ovipositor sheath **104** head anterior **105** head dorsal **106** hind leg.

### Gasteruption granulatum

Taxon classificationAnimaliaHymenopteraGasteruptiidae

Tan & van Achterberg sp. nov.

1F8099A0-4160-58C0-90E7-FF5FE54E68E1

http://zoobank.org/CF7781E9-377F-48E1-A7ED-C7F206D5D135

Figs 107–108 , 109–117 , 118–123

Gasteruption parvicollarium ; Zhao et al. 2012: 68–72 (p.p.: only ♀, not ♂).

#### Type material.

***Holotype*,** ♀ (NWUX), “NW China: Shaanxi, Luonan, Luoyuan, 16.iv.–28.v.2016, black [Malaise] trap, 1310 m alt., 34.21°N, 108.89°E, JL Tan & QQ Tan, NWUX”. ***Paratypes***: 1 ♀ (RMNH), topotypic, but xii.2017–17.vi.2018; 1 ♂ (NWUX), “NW China: Shaanxi, Baolongyu, Qin[ling] Mt[s], ca. 1000 m alt., 24.v.2015, 34°3'N, 108°9'E, Jiangli Tan, NWUX”.

**Figures 107–108.**
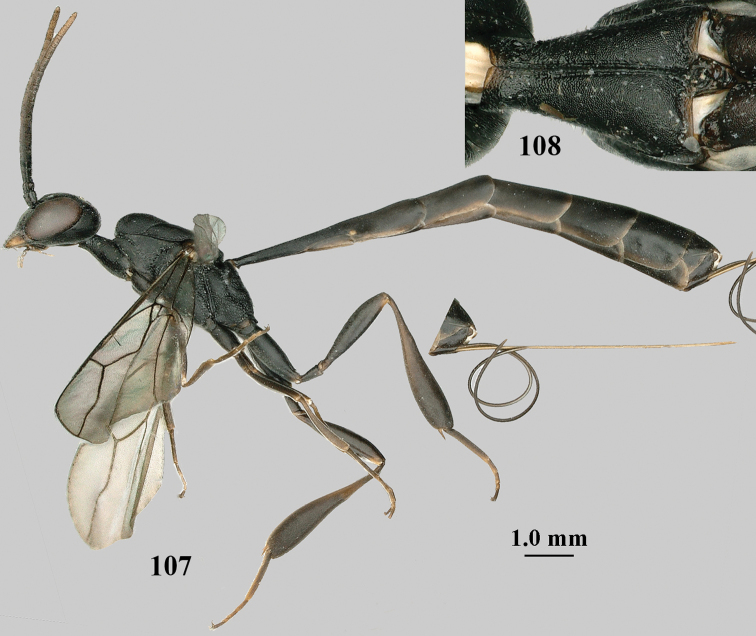
*Gasteruption granulatum* Tan & van Achterberg, sp. nov., female, holotype **107** habitus lateral **108** propleuron ventral.

#### Diagnosis.

Head of ♀ trapezoid in dorsal view, medio-posteriorly truncate or nearly so, in anterior view hardly protruding below lower level of eyes and mandibular condylus near lower level of eyes; occipital carina narrow and non-lamelliform medio-dorsally; vertex, at most, moderately bulging above upper level of eyes; clypeus with obsolescent depression; propleuron antero-dorsally granulate, rather robust in ventral and lateral; side of pronotum slender and with narrow and weakly crenulated grooves; mesoscutum matt and densely finely granulate; hind tibia slender and basitarsus elongate; hind tibia dark brown to yellowish-brown ventro-basally; apex of ovipositor narrow and with minute dorsal teeth; ovipositor sheath 1.2–1.8× as long as hind tibia and its apex mainly dark brown; apical sternite of ♂ and paramere entirely dark brown.

**Figures 109–117.**
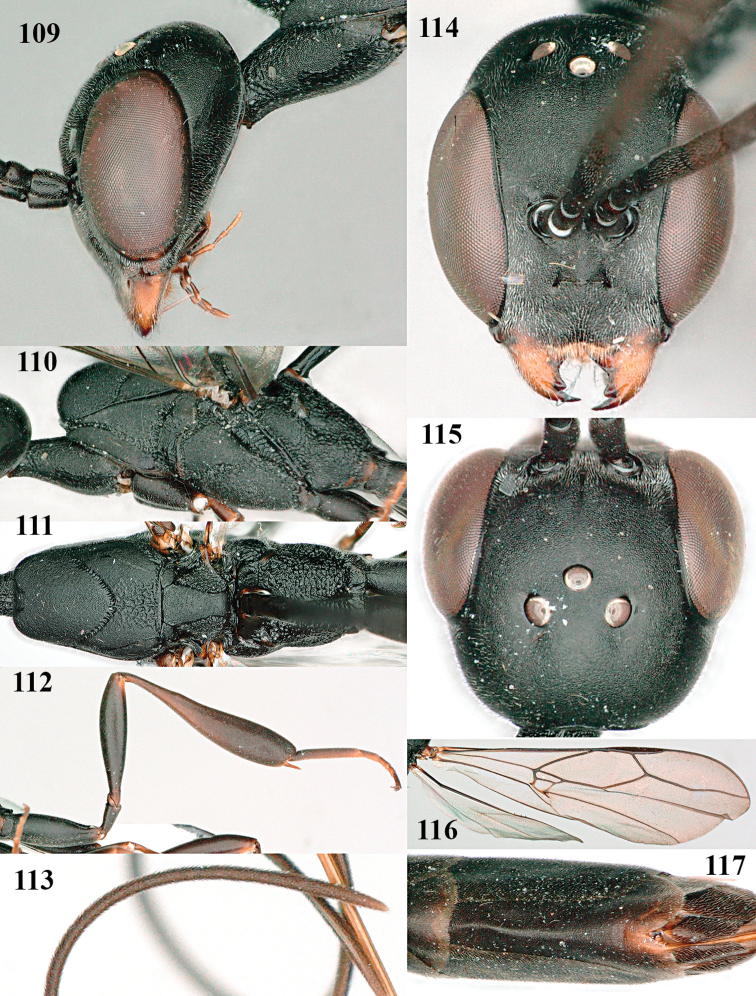
*Gasteruption granulatum* Tan & van Achterberg, sp. nov., female, holotype **109** head lateral **110** mesosoma lateral **111** mesosoma dorsal **112** hind leg **113** apex of ovipositor sheath **114** head anterior **115** head dorsal **116** wings **117** apex of metasoma ventral.

Easily confused with *G. parvicollarium* Enderlein, but *G. granulatum* has the vertex, at most, moderately bulging above upper level of eyes (strongly bulging in *G. parvicollarium*), head normal in dorsal view (distinctly elongated); apex of ovipositor narrow and with minute dorsal teeth (wider and with coarse dorsal teeth); propleuron less elongate in ventral view (more elongate) and robust in lateral view (somewhat more slender).

#### Description.

Holotype, female, length of body 14.0 mm, of fore wing 6.9 mm.

***Head*.** Vertex and frons with satin sheen, very finely granulate, moderately convex (Fig. 109) and without a depression medio-posteriorly; head gradually contracted behind eyes in dorsal view and temples curved (Fig. 115); temple 0.6× as long as eye in dorsal view; fourth antennal segment 1.2× as long as third segment and 0.7× as long as second and third segments combined, fifth antennal segment 1.1× as long as third segment, third antennal segment 1.7× as long as second segment; occipital carina narrow and non-lamelliform medio-dorsally (Figs 109 and 115); OOL twice as long as diameter of posterior ocellus; face wide, 2.1× as broad as high and combined height of eye and malar space 1.7× minimum width of face (Fig. 114); malar space not protruding below lower level of eyes (Fig. 114), its minimum width 0.2× basal width of mandible and area behind incision much wider than high (Fig. 109); clypeus only medio-ventrally shallowly depressed (Fig. 114); eye glabrous.

***Mesosoma*.** Length of mesosoma 2.1× its height; propleuron robust and 0.8× as long as mesoscutum in front of tegula (Fig. 110); pronotal side matt, sparsely setose and entirely finely granulate, except for narrow crenulated grooves, without acute tooth antero-ventrally, only corner rather rectangularly protruding (Fig. 110); propleuron densely and finely granulate, in ventral view, robust and nearly triangular (Fig. 108); antesternal carina narrow and hardly lamelliform; mesopleuron matt and mainly finely granulate, except for wide and coarsely crenulate depression and reticulate-rugose precoxal sulcus; mesosternal sulcus widened posteriorly and deep, coarsely transversely rugose; mesoscutum (but medio-posteriorly coarsely reticulate-punctate) and scutellum matt and densely finely granulate (Fig. 112), flat in lateral view (Fig. 107); scutellum perfectly flat; propodeum coarsely reticulate and with median smooth stripe. ***Wings*.** First discal cell slightly narrowed distally and with outer posterior corner rounded, with vein 3-CU1 near its apical quarter (Fig. 116). ***Legs*.** Hind coxa moderately slender (Fig. 112) and entirely finely granulate, matt; length of hind femur, tibia and basitarsus 4.3, 5.3 and 4.0× their width, respectively; hind tibia slender (Fig. 112); middle tarsus 1.3× as long as compressed middle tibia; middle femur subparallel-sided, compressed and slightly narrower than fore femur.

***Metasoma*.** Ovipositor sheath 5.3 mm, 0.4× as long as body, 0.5× as long as metasoma and 1.6× as long as hind tibia; ovipositor sheath with dense cover of fine brownish and adpressed setae, its apical half slender; ovipositor apex narrow and its dorsal teeth minute; hypopygium shallowly V-shaped emarginate medio-posteriorly (Fig. 117).

***Colour*.** Black; apical fifth of antenna largely brown; tegulum and mandible brownish-yellow (except narrow dark borders); clypeus latero-ventrally and humeral plate dark brown; third–sixth metasomal tergites very narrowly apically pale greyish, fourth–sixth sternites apically pale greyish; fore and middle legs (except coxae) largely, hind trochantellus, hind femur apico-ventrally, hind tibial spurs, hind basitarsus apically and more or less second-fourth hind tarsal segments, yellowish-brown; remainder of hind leg (including basal half of tibia and excluding coxa), veins and pterostigma dark brown; wing membrane slightly infuscate, except basally; apex of ovipositor sheath mainly dark brown (Fig. 113).

**Male.** Similar to female (including fine granulate sculpture of mesoscutum and scutellum, Fig. 119) and fore wing (except its basal quarter) distinctly brownish; length of fore wing 5.5 mm, of body 12.2 mm; third antennal segment 1.4× as long as second segment; fourth antennal segment 1.3× as long as third segment and 0.8× as long as second and third segments combined, fifth antennal segment 1.1× as long as third segment (Fig. 122); hind tibia slightly more yellowish ventro-basally (Fig. 120); apical sternite and paramere entirely dark brown (Fig. 123).

**Figures 118–123.**
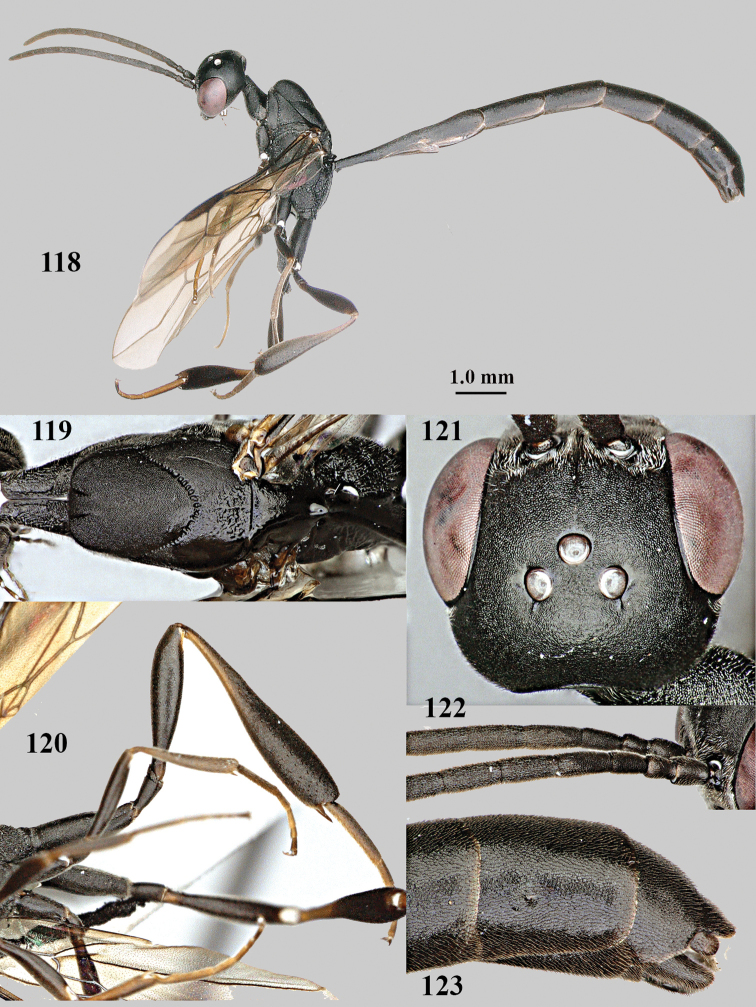
*Gasteruption granulatum* Tan & van Achterberg, sp. nov., male, paratype **118** habitus lateral **119** mesosoma dorsal **120** hind leg **121** head dorsal **122** basal antennal segments **123** apex of metasoma lateral.

#### Variations.

According to Zhao et al. (2012), ovipositor sheath sometimes 1.2× as long as hind tibia. The topotypic paratype has the ovipositor sheath 1.8× as long as hind tibia, length of body 12.2 mm and of ovipositor sheath 4.7 mm.

#### Distribution.

China (Shaanxi). According Zhao et al. (2012), Fujian, Guizhou, Hebei, Hubei, Hunan, Jiangsu, Shanxi, Taiwan and Zhejiang.

#### Etymology.

The name is derived from granulum (Latin for small grain or seed) because of the granulate mesoscutum and scutellum.

### Gasteruption japonicum

Taxon classificationAnimaliaHymenopteraGasteruptiidae

Cameron, 1888

7F64A023-3F0A-5F76-A404-281414B2619D

Gasteruption japonicum Cameron, 1888: 134; Zhao et al. 2012: 58–61; Tan et al. 2016: 97.
Gasteryption (!) *sibiricum* Semenov, 1892: 24; van Achterberg et al. 2019: 5 (synonymised with G. japonicum).
Gasteruption rufescenticorne Enderlein, 1913: 324–325 (only female lectotype; not Zhao et al. 2012); Hedicke 1939: 28; Pasteels 1958: 177. Syn. nov.
Gasteruption sinense var. minus Kieffer, 1924: 78; Zhao et al. 2012: 58 (synonymised with G. japonicum).

#### Additional material.

1 ♀ + 2 ♂ (NWUX) Shaanxi, NWU Taibai campus, small garden, ca. 410 m alt., on flowers of *Cayratia japonica* (Thunberg), 4, 6, 12.vii.2017, JL Tan/QQ Tan; 3 ♀, id., but 13.vi.2020, JL Tan; 1 ♀, NWU Chang’an campus, on flowers of *Daucus carota* L. 30.vi.2020, JL Tan; 3 ♀ (NWUX, RMNH), id., but 5, 15, 23.vi.2018, JL Tan/QQ Tan/RN Zhang; 1 ♀ (NWUX), Shaanxi, near Ningqiang, 23.vii.2017 QQ Tan; 2 ♀ (NWUX), Shaanxi, Bailuyuan, Baqiao, Xi’an, 34.20°N, 109.12°E, 14.vii.2018, 687 m, Ruonan Zhang; 1 ♀ + 2 ♂ (NWUX), Shaanxi, Luonan, Shangluo, 34.02°N, 110.10°E, 9.vii.–9.ix.2017, 1006 m alt., yellow Malaise trap, JL Tan & QQ Tan; 2 ♀ (NWUX), Shaanxi, Taibai Mt., Baoji, 34.04°N, 107.46°E, swept, 4.viii.2017, 1251 m alt., Jiangli Tan; 1 ♀ (NWUX) Shaanxi, Xunyangba, Ningshan, 32°91'N, 109°68'E, 9.vi.2019, 507, Tan JL & Zhang RN; 2 ♀ (RMNH), Shaanxi, Qinling Mts., Luonan, Maping, Yunmeng Mt., 9–10.vii.2017, ca. 1090 m alt., 34°8'N, 110°7'E, C. v. Achterberg; 6 ♀ (NWUX), id., but 34.08°N, 110.02°E, 1084 m alt., Ruonan Zhang/Qingqing Tan/Jiangli Tan; 1 ♀ (NWUX), NE China: Shaanxi, Xi’an, NWU Taibai campus, small garden, ca. 410 m alt., 3.ix.2018, JL Tan; 2 ♀ (NWUX, RMNH), NW China: Shaanxi, Yingpan, Zhashui, 859 m alt., 33°73'N, 109°88'E, 1.vii.2019, Jiangli Tan; 1 ♀ (NWUX), NW China: Shaanxi, Qiligou, Xunyangba, 507 m alt., 32°91'N, 109°68'E, 1.vii.2019, hand net, JL Tan; 1♀ (NWUX), Shaanxi, Miaojv, Liulin, Yaozhou, Tongchuan, sweep net, 35.60°N, 108.49°E, 27.vii.2019, 934 m alt., Jiangli Tan.

#### Notes.

The female lectotype of *Gasteruption rufescenticorne* Enderlein proved to be a synonym of *G. japonicum* (syn. nov.). Zhao et al. (2012) used the paralectotype of *G. rufescenticorne*, but this specimen belongs to a new species (*G. kexinae*) described below.

#### Distribution

. China (Fujian, Gansu, Heilongjiang, Hubei, Hunan, Inner Mongolia, Jilin, Ningxia, Shaanxi, Shanghai, Sichuan, Taiwan, Xinjiang, Yunnan, Zhejiang); Japan (Honshu, Hokkaido).

### Gasteruption kexinae

Taxon classificationAnimaliaHymenopteraGasteruptiidae

Tan & van Achterberg sp. nov.

B98125C2-7E9F-5761-A771-B3746160BAC6

http://zoobank.org/64A374A5-E70C-4C12-A2F5-D1ABD49A729F

Figs 124–127 , 128–136 , 137–138 , 139–143

Gasteruption rufescenticorne ; Zhao, van Achterberg & Xu, 2012: 75–80 (paralectotype; not lectotype).

#### Type material.

***Holotype*,** ♀ (NWUX), “S. China: Fujian, Tianbaoyan, Yong-an, 25°53'42"N, 117°28'05"E, 26.vi.2018, Mal. trap, alt. 530 m, Lingfei Peng, NWUX”. ***Paratypes***: 2 ♂ (NWUX, RMNH), topotypic, but x–xii.2018; 1 ♀ (RMNH), “S. China: Fujian, Mt. Longqi, Sanming, 26°31'27"N, 117°17'27"E, 13.vii.2018, Mal. trap, alt. 740 m, Lingfei Peng, NWUX”; 1 ♀ + 1 ♂ (NWUX), “S. China: Fujian, Tianbaoyan, Yong-an, 25°53'42"N, 117°28'05"E, 22.v.–12.vi.2018, Mal. trap, 530 m alt., Lingfei Peng, NWUX”; 1 ♀ (NWUX), “S China: Fujian, Huboljiao, Nanping, 24°54'24"N, 117°12'52"E, 1.viii.2018, Mal. trap, 300 m alt., Lingfei Peng, NWUX”; 1 ♂ (RMNH), “S. China: Fujian, Mt. Longqi, Sanming, 26°31'27"N, 117°17'27"E, 13.vii.2018, Mal. trap, 740 m alt., Lingfei Peng, NWUX”; 1 ♀ + 1 ♂ (ZJUH), “[China:] Zhejiang, Hangzhou, 4.vii.1980”; 1 ♂ (ZJUH), id., but 21.vii.1980; ♀ (ZJUH), “[China:] Zhejiang, Huizhou, Shishi, 25.ix.1984, Cai-e Zhou; 1 ♂ (ZJUH), “[China:] Fujian, Fuzhou, Jinshan, 20.vi.1990, Xiu-fu Zhao”; 1 ♂ (ZJUH), id., but 14–18.x.1990; 2 ♂ (ZJUH), id., but 17.viii.1990, Chang-ming Liu; 2 ♂ (ZJUH, RMNH), id., but 15.vi.1988, Saping Yi; 1 ♀ + 1 ♂ (ZJUH), “[China:] Hunan, Liuyang, 30.V.1984, Xin-wang Tong”; 1 ♀ (ZJUH), “[China:] Hunan, Liuyang, 25.V.1986, Xin-wang Tong”; 1 ♂ (ZJUH), “[China:] Hunan, Liuyang, 13.V.1985, Xin-wang Tong”.

**Figures 124–127.**
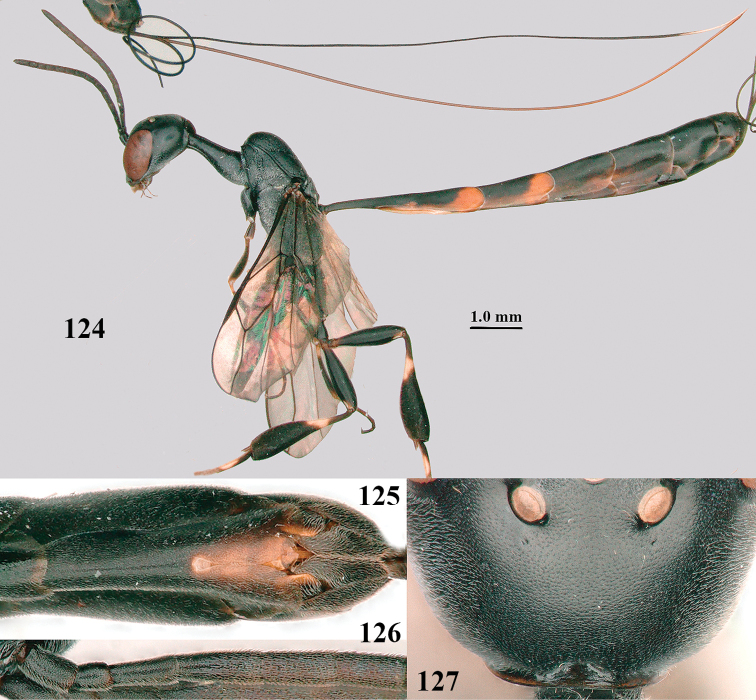
*Gasteruption kexinae* Tan & van Achterberg, sp. nov., female, holotype **124** habitus lateral **125** apex of metasoma ventral **126** base of antenna **127** details of vertex dorsal.

#### Diagnosis.

**Figures 128–136.**
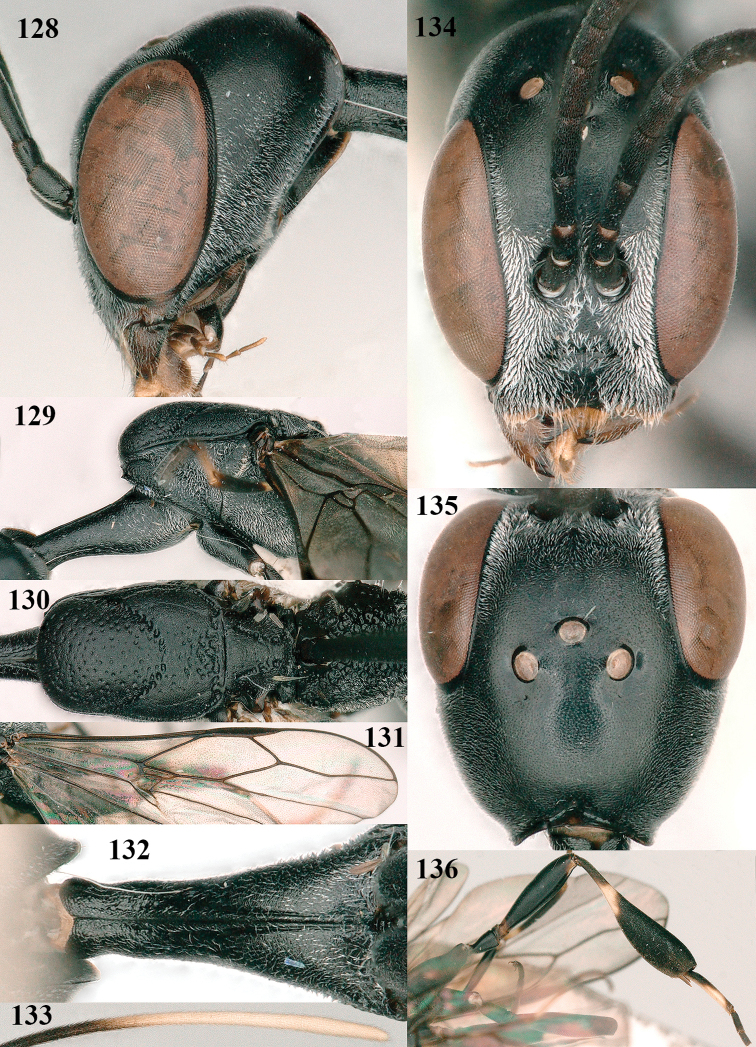
*Gasteruption kexinae* Tan & van Achterberg, sp. nov., female, holotype **128** head lateral **129** mesosoma lateral **130** mesosoma dorsal **131** wings **132** propleuron ventral **133** apex of ovipositor sheath **134** head anterior **135** head dorsal **136** hind leg.

Head of ♀ comparatively long and nearly parallel-sided behind eyes in dorsal view, with deep medial depression in front of occipital carina and with pair of shallow lateral depressions (Fig. 127); vertex medially distinctly above level of ocelli in lateral view, densely punctulate and long (Fig. 128); occipital carina wide lamelliform (Figs 128 and 135); propleuron in lateral view slender and 1.0–1.2× as long as mesoscutum in front of tegula (Fig. 129) and in ventral view narrow anteriorly (Fig. 132); middle lobe of mesoscutum without fine transverse elements anteriorly, only very finely coriaceous between distinct punctures and posteriorly vermiculate (Fig. 130); ovipositor sheath about 0.9× as long as body, its white or ivory apical part about 1.5× as long as hind basitarsus.

Easily confused with *G. corniculigerum* Enderlein, 1913; differs mainly by the shape of the head in dorsal view (nearly parallel-sided behind eyes in female and distinctly contracted in *G. corniculigerum*), the sculpture of the middle lobe of the mesoscutum (without fine transverse elements anteriorly; present in *G. corniculigerum*), the anteriorly more slender propleuron and the shorter pale part of the ovipositor sheath (1.0–2.1× versus 2.8–3.5× as long as hind basitarsus in *G. corniculigerum*).

#### Description.

Holotype, female, length of body 16.1 mm, of fore wing 6.5 mm.

***Head*.** Vertex and frons with satin sheen and densely punctulate; vertex weakly convex medio-posteriorly in front of deep medio-posterior depression and a pair of shallow elliptical lateral depressions (Figs 127 and 135); in lateral view vertex rounded in front of depression and distinctly above level of ocelli (Fig. 128); occipital carina wide lamelliform medio-dorsally, about half as wide as diameter of posterior ocellus, equally blackish basally and gradually becoming paler distally (Fig. 128); head rather gradually narrowed behind eyes in dorsal view and temples rounded (Fig. 135); temple 0.7× as long as eye in dorsal view; fourth antennal segment 1.3× as long as third segment and 0.9× as long as second and third segments combined, fifth antennal segment 1.1× as long as third segment, third antennal segment twice as long as second segment; OOL twice as long as diameter of posterior ocellus; diameter of anterior ocellus equal to distance between anterior and posterior ocelli; face 2.3× as broad as high; combined height of eye and malar space twice minimum width of face; malar space short, its minimum width 0.2× basal width of mandible and area behind incision nearly triangular (Fig. 128); clypeus only medio-ventrally shallowly depressed and ventrally with long golden bristles (Fig. 134); eye glabrous, except for numerous spaced and very short setae.

***Mesosoma*.** Length of mesosoma 2.2× its height; propleuron rather slender and 1.2× as long as mesoscutum in front of tegula, in ventral view anteriorly distinctly narrowed; pronotal side rugose ventrally, setosity not obscuring sculpture and crenulated grooves wide, antero-ventral tooth small and triangular (Fig. 129); antesternal carina narrow and narrowly lamelliform; mesosternal sulcus wide and coarsely crenulate, parallel-sided and deep; middle lobe of mesoscutum medially rather dull, very finely coriaceous between spaced and coarse punctures (Fig. 130), in lateral view hardly protruding (Fig. 129); mesoscutum medio-posteriorly coarsely vermiculate and dorso-laterally largely coriaceous with some shallow punctures; scutellum finely coriaceous (Fig. 130); propodeum coarsely reticulate and with median carina anteriorly and median stripe posteriorly. ***Wings*.** First discal cell parallel-sided and with outer posterior corner rounded, with vein 3-CU1 near its apical third (Fig. 131). ***Legs*.** Hind coxa robust, coarsely reticulate, but dorsally coarsely transversely rugose; length of hind femur, tibia and basitarsus 3.7, 4.3 and 6.2× their width, respectively; hind tibia distinctly inflated (Fig. 136); middle tarsus 1.2× as long as middle tibia; middle femur subparallel-sided and distinctly more slender than fore femur.

***Metasoma*.** Ovipositor sheath 14.6 mm, 0.9× as long as body, 1.4× as long as metasoma and 5.6× as long as hind tibia; ovipositor sheath with dense cover of very fine adpressed setae, its apical white part 1.5× as long as hind basitarsus; emargination of hypopygium 0.4× length of hypopygium (Fig. 125).

***Colour*.** Black; antenna (except blackish four basal segments and apical segment) largely dark brown; mandible largely brown (Fig. 128); clypeus latero-ventrally black; labial palpi brown, tegulum and humeral plate dark brown; fore and middle tibiae basally, largely fore and middle basitarsi, large elliptical baso-ventral patch (including narrow dorsal part) of hind tibia, hind basitarsus (but basal third dark brown and dorso-apically infuscate) and apex of ovipositor sheath ivory or white (Fig. 133); trochantelli brown; second and third metasomal tergites apically conspicuously yellowish-brown; veins and pterostigma dark brown; wing membrane slightly brownish.

**Male.** Very similar to female (including fine coriaceous sculpture of mesoscutum, Fig. 139), but hind basitarsus entirely dark brown (Fig. 140) and wings brownish; antenna (except 4 basal segments) ventrally or entirely brown or dark brown; third antennal segment 1.5–1.8× as long as second segment; fourth antennal segment 1.9–2.3× as long as third segment and 1.1–1.7× as long as second and third segments combined, fifth antennal segment 1.9–2.1× as long as third segment (Fig. 141); apical and pre-apical sternite entirely dark brown; paramere densely whitish setose and its apex ivory or pale yellowish (Fig. 138).

**Figures 137–138.**
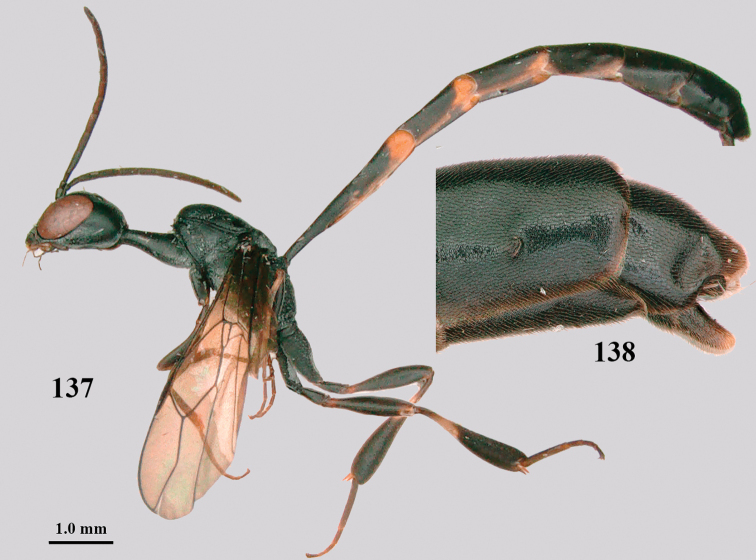
*Gasteruption kexinae* Tan & van Achterberg, sp. nov., male, paratype **137** habitus lateral **138** apex of metasoma lateral.

#### Variations.

Body length of ♀ 14.6–16.1 mm, of ♂ 12.5–16.5 mm; propleuron 1.0–1.2× as long as mesoscutum in front of tegula; ovipositor sheath 0.9–1.0× as long as body; white or ivory apical part of ovipositor sheath 1.0–2.1× longer than hind basitarsus; antenna (except basally) dark brown or brown.

#### Distribution.

China (Fujian, Guangxi, Hainan, Hubei, Hunan, Jiangsu, Ningxia, Shanghai, Taiwan, Zhejiang).

#### Etymology.

Named after the first author of the revision of the Gasteruptiidae from China, Ms Ke-xin Zhao, for her excellent cooperation and taxonomical insight.

#### Notes.

The female lectotype of *G. rufescenticorne* Enderlein became available after the revision by Zhao et al. (2012) was completed. The interpretation of this species was based on the male paralectotype, which proved to belong to another species rather than the lectotype. The species, based on the male paralectotype, is here described as *G. kexinae* sp. nov. and the real *G. rufescenticorne*, based on the lectotype, is synonymised with *G. japonicum* (syn. nov.).

**Figures 139–143.**
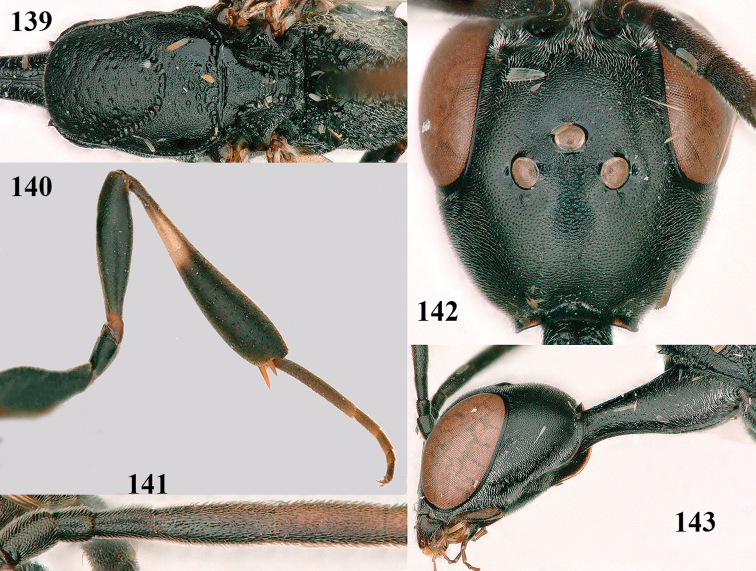
*Gasteruption kexinae* Tan & van Achterberg, sp. nov., male, paratype **139** mesosoma dorsal **140** hind leg **141** base of antenna **142** head dorsal **143** head lateral.

### Gasteruption latitibia

Taxon classificationAnimaliaHymenopteraGasteruptiidae

Zhao, van Achterberg & Xu, 2012

E0FECDA5-F457-5ACB-AC63-B0618607502D

Figs 144 , 145–154

Gasteruption latitibia Zhao, van Achterberg & Xu, 2012: 62–65.

#### Additional material.

18 ♀ + 5 ♂ (NWUX, RMNH), “NW China: Shaanxi, Huaishuzhuang Rev. St., Ziwuling NNR, Fuxian, Yanan, sweep net, 35.86°N, 108.74°E, 1–8.viii.2019, alt. 1127 m, Jiangli Tan/Ruonan Zhang, NWUX”; 1 ♀ (NWUX), “NW China: Shaanxi, Miaojv, Liulin, Yaozhou, Tongchuan, sweep net, 35.60°N, 108.49°E, 27.vii.2019, alt. 934 m, Jiangli Tan, NWUX”; 1 ♂ (NWUX), “NW China: Shaanxi, Qiligou, Xinyangba, Ningshan, sweep net, 32.91°N, 109.68°E, 19.viii.2019, alt. 1508 m, Ruonan Zhang, NWUX”; 2 ♀ (RMNH), Shaanxi, Qinling Mts., Luonan, Maping, Yunmeng Mt., 9–10.vii.2017, ca. 1090 m alt., 34°8'N, 110°7'E, C. v. Achterberg; 6 ♀ (NWUX), id., but 34.08°N, 110.02°E, 1084 m alt., Ruonan Zhang/Qingqing Tan/Jiangli Tan; 1 ♀ (NWUX), NE China: Shaanxi, Xi’an, NWU Taibai campus, small garden, ca. 410 m alt., 3.ix.2018, JL Tan; 1 ♀ (NWUX), NW China: Shaanxi, Qiligou, Xunyangba, 507 m alt., 32°91'N, 109°68'E, 1.vii.2019, hand net, JL Tan; 1 ♀ (RMNH), NW China: Shaanxi, 40 km N of Foping, ca. 1250 m alt., from wood stack, 25.vi.2017, near road to Xi’an, C. v. Achterberg; 2 ♀ (NWUX, RMNH), NW China: Shaanxi, Yingpan, Zhashui, 859 m alt., 33°73'N, 109°88'E, 1.vii.2019, Jiangli Tan; 1 ♀ (RMNH), E China: Zhejiang, Mt. Tianmu, 20.vii.2015, C. van Achterberg.

**Figure 144.**
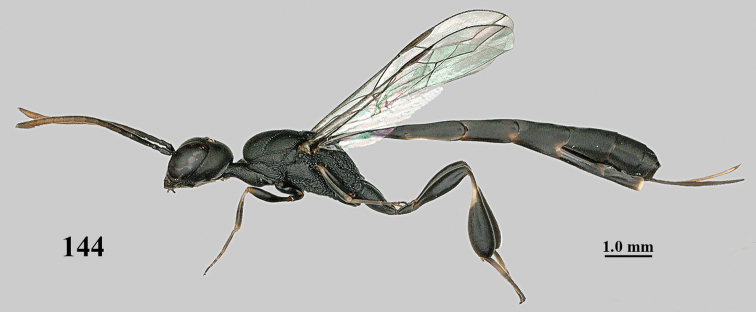
*Gasteruption latitibia* Zhao, van Achterberg & Xu, female, Shaanxi, habitus lateral.

#### Distribution.

China (Fujian, Guizhou, Hubei, Hunan, Shaanxi (410–1508 m alt.), Zhejiang). New for Shaanxi and Zhejiang.

**Figures 145–154.**
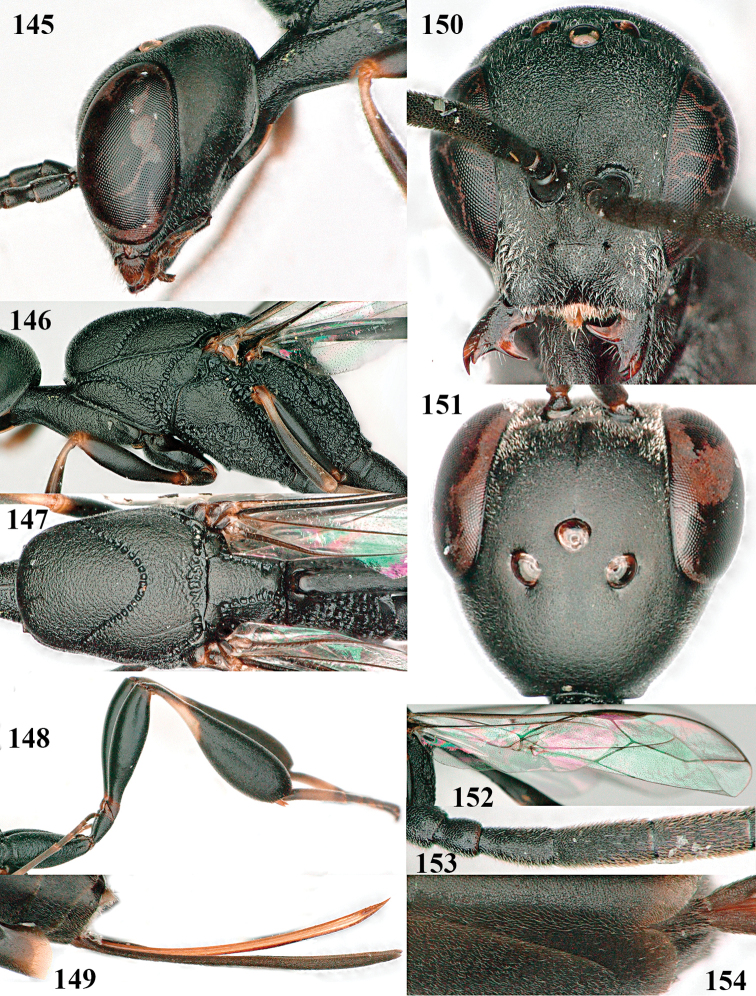
*Gasteruption latitibia* Zhao, van Achterberg & Xu, female, Shaanxi **145** head lateral **146** mesosoma lateral **147** mesosoma dorsal **148** hind leg **149** ovipositor and its sheath lateral **150** head anterior **151** head dorsal **152** wings **153** base of antenna **154** hypopygium ventral.

### Gasteruption minutum

Taxon classificationAnimaliaHymenopteraGasteruptiidae

(Tournier, 1877)

4072F831-7A8D-5C45-A8F5-FAF4F0864C6B

Figs 155–156 , 157–165 , 166–167 , 168–173

Foenus minutus Tournier, 1877: ix; Capron 1880: 89; Schletterer 1889: 398 (as synonym of G. assectator (Linnaeus)).
Faenus minutus ; Abeille de Perrin 1879: 265, 267, 277.
Gasteruption minutum ; Kieffer 1912: 257; Hedicke 1939: 16; Ferrière 1946: 235, 238, 240; Hellén 1950: 4; Crosskey 1951: 295; Šedivý 1958: 36, 37, 40; Schmidt 1969: 295; Hedqvist 1973: 185; Dolfuss 1982: 24; Alexander 1983: 150; Allen 1983: 82; Madl 1987a: 403, 1987b: 23, 1988c: 38, 1989a: 160, 1989b: 44, 1990a: 128, 1990b: 480, 482; Kozlov 1988: 245, 247; Neumayer et al. 1999: 220; Kofler and Madl 1990: 322; Wall 1994: 159; Scaramozzino 1995: 3; Pagliano and Scaramozzino 2000: 11, 19, 30; Saure 2001: 29; Wisniowski 2004: 118; van der Smissen 2010: 373; van Achterberg 2013: fig. 172; Lotfalizadeh et al. 2017: 146.
Foenus longigena Thomson, 1883: 849; Ferrière 1946: 240; Hedqvist 1973: 185; Madl 1988c: 39. Synonymised with G. minutum (Tournier) by Ferrière 1946, Schmidt 1969 and Hedqvist 1973.
Gasteruption longigena ; Schletterer 1889: 399; Dalla Torre 1902: 1068; Kieffer 1912: 270; Schmiedeknecht 1930: 380, 381; Hedicke 1939: 16; Hellén 1950: 4 (as G. ”*longiserra*” and as synonym of G. minutum (Tournier)); Hedqvist 1973: 185 (lectotype designation); Wall 1994: 149.
Gasteruption oriplanum Kieffer, 1911: 210; Hedicke 1939: 27; Zhao et al. 2012: 65–68. Syn. nov.

#### Type material.

***Lectotype*** of *G. minutum* here designated, ♀ (MHNG) “[Switzerland], Peney, [near Genève], vii.[18]75”, “Cn Tournier”, “Type”, *Foenus minutus* Tourn., ♀”, “Lectotypus, des. Madl, 1987”. ***Paralectotypes*** (4 ♀, MHNG) and all from Peney, 2 ♀ collected vii.1876, 1 ♀vii.1875 and 1 ♀ 10.vi.1875; the paralectotypes from France and Italy were not found. ***Lectotype*** of *G. longigena* ♀, (ZIL) “Rõn” [= Rönnemölla, Skane-Norrland], “Lectotypus *Foenus longigena* Thoms., ♀, K.-J. Hedqvist, det. 1972”. Holotype of *G. oriplanum*, ♂ (BMNH), “Type”, “B. M. Type Hym. 3.a.173”, “*Gasteruption oreiplanus* [sic!] Kieff.”, “[China:], Tibet, Gyangtse, 13,000 ft. [3960 m alt.], June 1904, Tibet Exped., H.J. Walton, 1905-172/ 29.vi.1904”.

**Figures 155–156.**
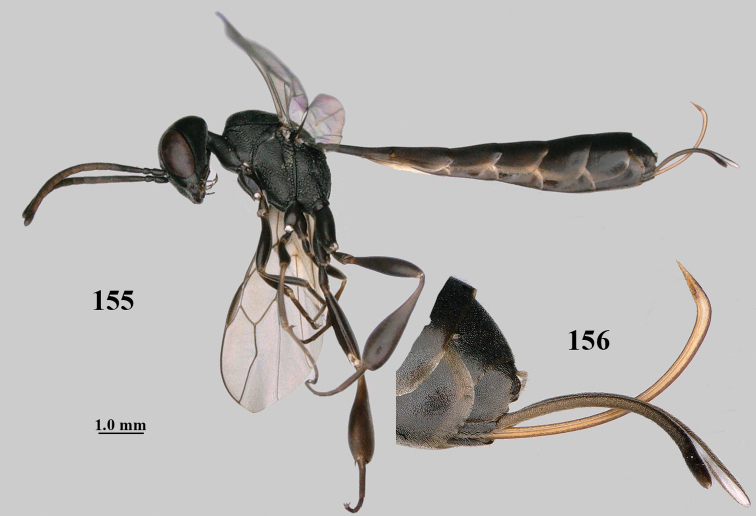
*Gasteruption minutum* (Tournier), female, Shaanxi **155** habitus lateral **156** ovipositor and its sheath lateral.

#### Additional material.

1 ♂ (NWUX), NW China: Shaanxi, Xunyangba, 33.55°N, 108.55°E, 1.vii.2017, 1481 m alt., Jiangli Tan; 5 ♀ (NWUX, RMNH), Shaanxi, Xunyangba, Ningshaan, 1.vii.2018, ca. 1480 m alt., 33°54'N, 108°55'E, Jiangli Tan.

**Figures 157–165.**
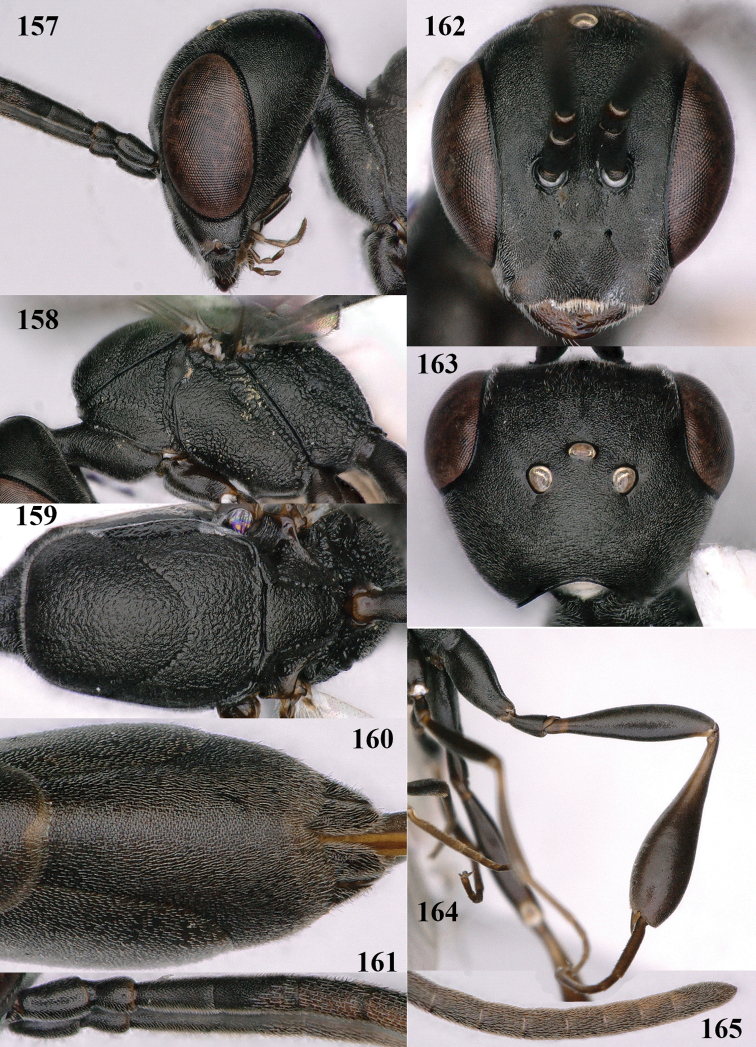
*Gasteruption minutum* (Tournier), female, Shaanxi **157** head lateral **158** mesosoma lateral **159** mesosoma dorsal **160** apex of metasoma ventral **161** base of antenna **162** head anterior **163** head dorsal **164** hind leg **165** apex of antenna.

#### Distribution.

China (Shaanxi [1480 m], Tibet [2800–4300 m]), South Palaearctic, Central Europe. New for Shaanxi.

**Figures 166–167.**
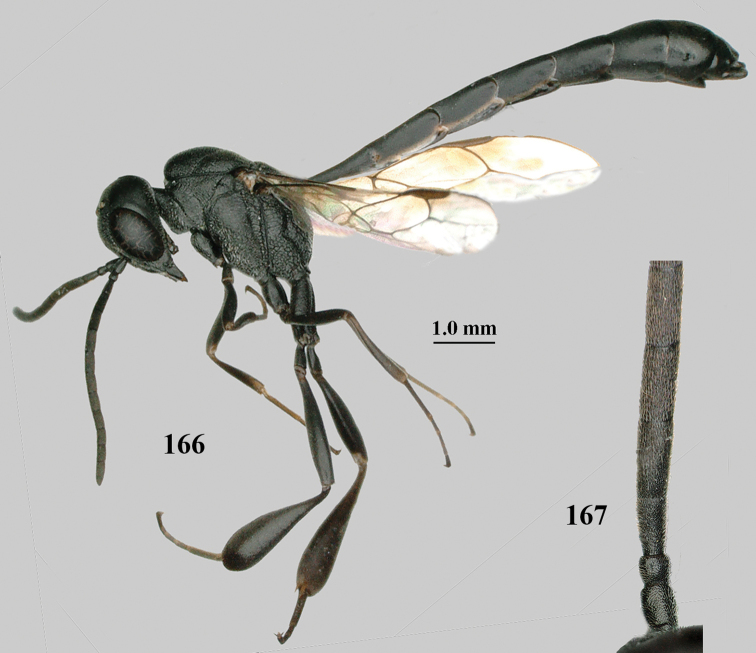
*Gasteruption minutum* (Tournier), male, Shaanxi **166** habitus lateral **167** basal antennal segments.

#### Notes.

Unfortunately, the holotype of *G. oriplanum* is a male and has the head severely damaged (figs 166–173 in Zhao et al. 2012). An additional female from Tibet is known (Zhao et al. 2012) and, judging from this female, *G. oriplanum* is a synonym of the South Palaearctic species (with intrusions in Central Europe) *G. minutum* (Tournier, 1877). The new finds in Shaanxi, together with additionally examined specimens from Central Asia, make this new synonymy more plausible, despite the variation in the shape of the hind tibia. The holotype of *G. oriplanum* has the hind tibia strongly inflated, much more than the male from Shaanxi (fig. 169 in Zhao et al. (2012) versus Fig. 166), but the Central European males have the hind tibia intermediately widened. The variation is probably clinal, could not be linked to other variations and, therefore, we consider *G. oriplanum* and *G. minutum* conspecific.

**Figures 168–173.**
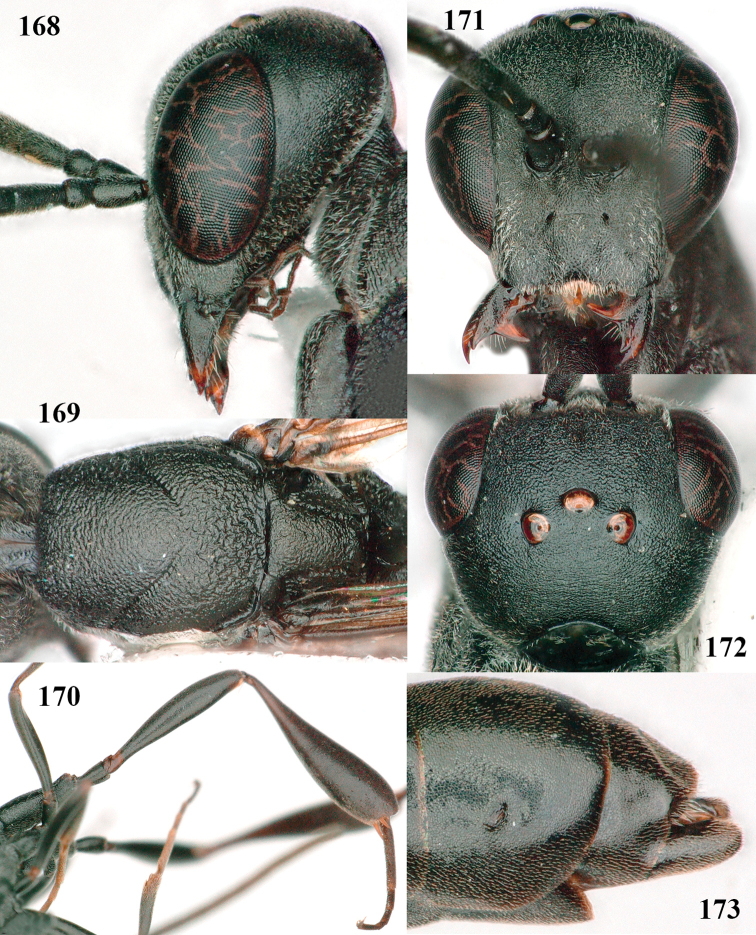
*Gasteruption minutum* (Tournier), male, Shaanxi **168** head lateral **169** mesosoma dorsal **170** hind leg lateral **171** head anterior **172** head dorsal **173** apex of metasoma lateral.

### Gasteruption nigritarse

Taxon classificationAnimaliaHymenopteraGasteruptiidae

(Thomson, 1883)

DEA3761C-ACF5-58AF-8FEB-08EFF4CC3B48

Figs 174–175 , 176–187 , 188 , 189–194

Foenus nigritarsis Thomson, 1883: 849; Schletterer 1889: 398; Hedicke 1939: 7; Hedqvist 1973: 181, 182 (lectotype designation); Wall 1994: 149. Synonymised with G. assectator (Linnaeus) by Schletterer (1889).
Gasteruption nigritarse ; Schletterer 1885: 310; Johansson and van Achterberg 2016: 84–86; Orlovskytė et al. 2018: 123.

#### Type material.

The female ***lectotype*** from Lund (Scania, S Sweden) was selected by Hedqvist (1973). All 12 specimens (both males and females) of the series under *Foenus nigritarsis* in ZIL (including the lectotype by Hedqvist) belong to the same distinct species (Johansson and van Achterberg 2016).

**Figures 174–175.**
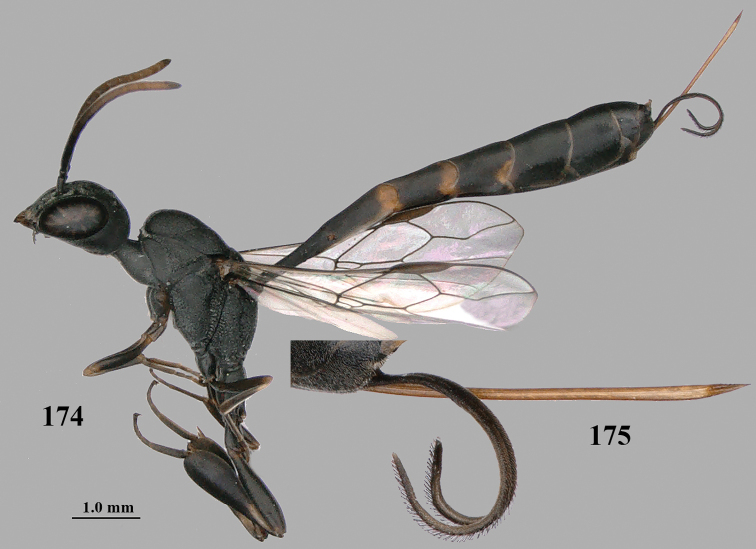
*Gasteruption nigritarse* (Thomson), female, Shaanxi **174** habitus lateral **175** ovipositor and its sheath lateral.

#### Additional material.

3 ♀ (NWUX), NW China: Shaanxi, Qiligou, Xunyangba, 507 m alt, 1.vii.2019, 32°91'N, 109°68'E, hand net, Tan JL; 1 ♀ + 1 ♂ (RMNH), NW China: Shaanxi, NWU Taibai campus, ca. 410 m alt., 34°15'N, 108°55'E, small garden, on flowers of *Cayratia japonica* (Thunberg), 4.viii.2017, C. v. Achterberg; 2 ♀, NWU Chang’an campus, on flowers of *Daucus carota* L. 30.vi.2020, JL Tan; 1 ♀ (NWUX), “NW China: Gansu, Lianjiabian, Taibai Heshui, sweep net, 36.06°N, 108.54°E, 8.viii.2019, alt. 1193 m, Ruonan Zhang, NWUX”; 4 ♀ (NWUX, RMNH), “NW China: Shaanxi, Xialiangzhen, Zhashui, Shangluo, sweep net, alt. 1059 m, Ruonan Zhang, NWUX”.

**Figures 176–187.**
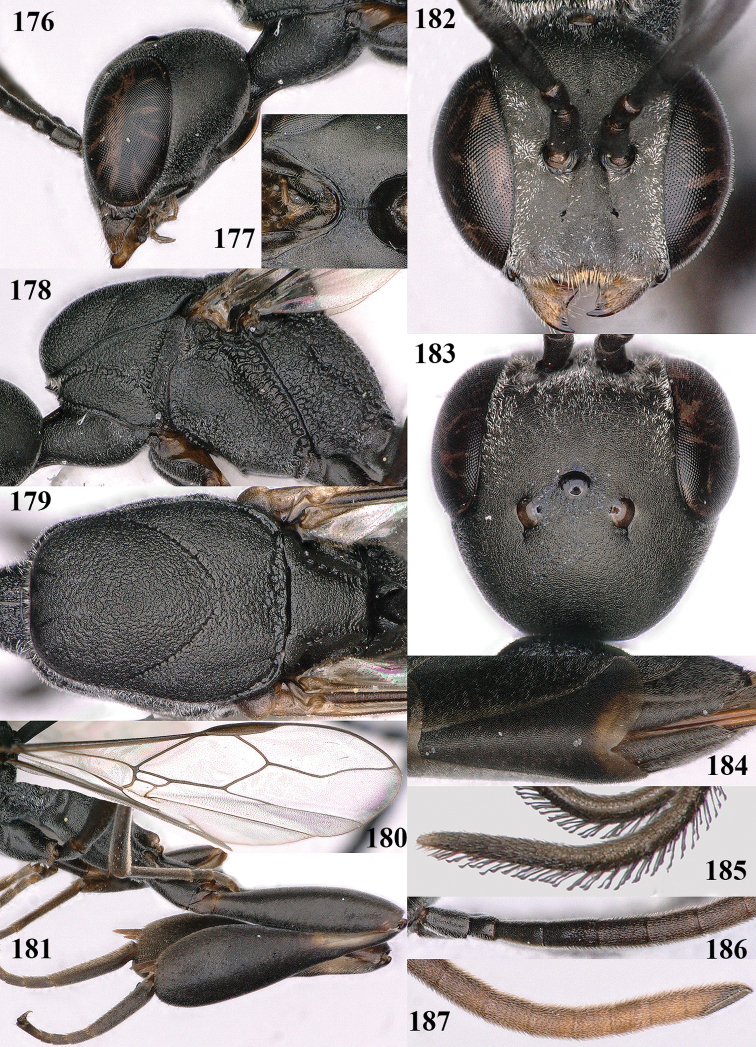
*Gasteruption nigritarse* (Thomson), female, Shaanxi **176** head lateral **177** details of hypostomal bridge **178** mesosoma lateral **179** mesosoma dorsal **180** fore wing **181** hind leg **182** head anterior **183** head dorsal **184** apex of metasoma ventral **185** details of apex of ovipositor sheath **186** base of antenna **187** apex of antenna.

#### Distribution.

Europe; China (Gansu, Shaanxi). Collecting a couple of this species in the small NWU garden (at the Taibai campus in the very centre of Xi’an) on bush-killer, *Cayratia japonica* (Thunb.) Gagnep. was a real surprise. New for China, Gansu, Shaanxi and even for the East Palaearctic Region.

**Figure 188.**
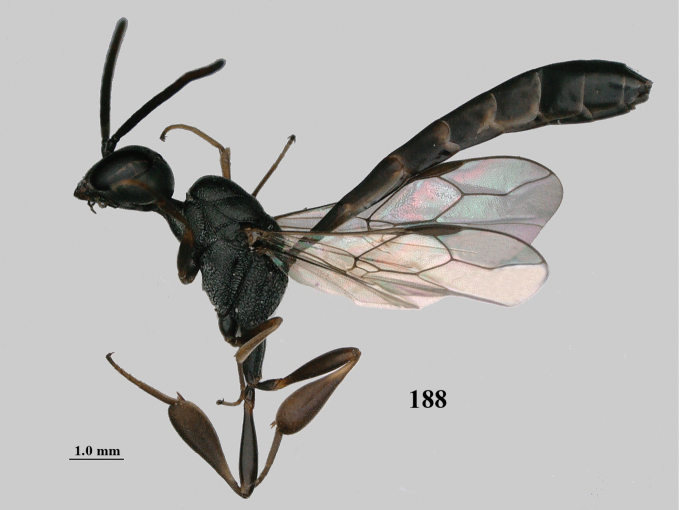
*Gasteruption nigritarse* (Thomson), male, Shaanxi, habitus lateral.

#### Notes.

The Chinese specimens have the temple slightly longer than in European specimens (the eye/temple ratio is the same in the examined specimens) and the hind tibia is slightly more inflated. Both are likely part of clinal variation and, therefore, the Chinese specimens are considered to be conspecific.

**Figures 189–194.**
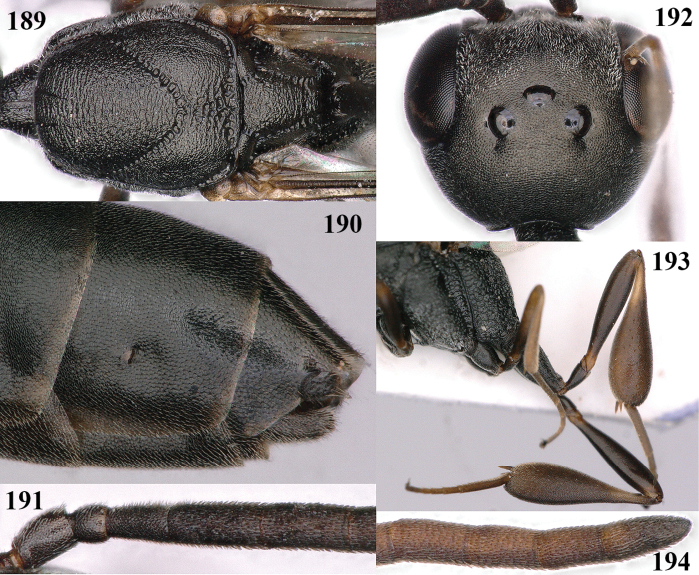
*Gasteruption nigritarse* (Thomson), male, Shaanxi **189** mesosoma dorsal **190** apex of metasoma lateral **191** base of antenna **192** head dorsal **193** hind legs **194** apex of antenna.

### Gasteruption oshimense

Taxon classificationAnimaliaHymenopteraGasteruptiidae

Watanabe, 1934

724ABBD0-0A33-5285-AB1E-A540875819DE

Figs 195–197 , 198–207 , 208–212

Gasteruption oshimensis Watanabe, 1934: 283–284.
Gasteruption oshimense ; Tan et al. 2016: 99–100, p.p.; van Achterberg et al. 2019: 6.

#### Additional material.

2 ♂ (NWUX), NW China: Shaanxi, Hanzhong, Liuba, Zibai Mt. Nat. Res., 33.66°N, 106.78°E, 5.ix.2015, ca. 1627 m alt., Jiangli Tan; 1 ♂ (NWUX), Shaanxi, Foping, behind Biological Station, Malaise trap, 33°39'29"N, 107°48'25"E, 29.v.–19.vi.2016, ca. 1710 m alt., JL. Tan & C. v. Achterberg; 7 ♀ + 5 ♂ (NWUX, RMNH), Shaanxi, Lantian Ape Man Site, 34.10N, 110.30E, 8.vii.2017, 775 m alt., Qingqing Tan/Ruonan Zhang/Jiangli Tan; 1 ♀ + 1 ♂ (RMNH), id., 8–9.vii.2017, ca. 735 m alt., 34°11'N, 109°29'E, C. v. Achterberg; 1 ♀ (NWUX), id., but 21.vi.2018, Ruonan Zhang; 1 ♀ (RMNH), Shaanxi, Daba Mts, Hanzhong, Tiankeng, Chanjiayan, E. of Ningqiang, ca. 1750 m alt., 32.45°N, 106.32°E, from wooden shed, 22.vii.2017, C. v. Achterberg; 1 ♀ (NWUX), Shaanxi, Xunyangba, Ninshan, 33.54°N, 108.35°E, 1439 m alt., 10.vi.2018, JL Tan; 2 ♀ (NWUX, RMNH), Shaanxi, Bailuyuan, Baqiao, Xi’an, 34.20°N, 109.12°E, 14.vii.2018, 687 m alt., Ruonan Zhang; 3 ♀ + 2 ♂ (NWUX, RMNH), Shaanxi, Luonan, Shangluo, 34.02°N, 110.10°E, 9.vii.–9.ix.2017, 1006 m alt., yellow Malaise trap, JL Tan & QQ Tan; 1 ♀ (NWUX), id., but black Malaise trap, xii.2017–17.vi.2018; 1 ♂ (NWUX), Shaanxi, Huanghualing, Zhasui, 33.80°N, 108.88°E, 17.viii.2016, 1408 m alt., Jiangli Tan; 2 ♀ + 4 ♂ (NWUX, RMNH), Shaanxi, near Ankang, Langoa, 32°17'1"N, 109°03'46"E, ca. 1100 m alt., 11.vi.2016, JL. Tan, QQ. Tan & C. van Achterberg; 2 ♀ (NWUX, RMNH), Shaanxi, Zhashui, Huanghualing, 33.76°N, 108.85°E, 23.vii.2015, ca. 1577 m alt., Jiangli Tan; 1 ♀ (NWUX), Shaanxi, Ningqiang, Hanzhong, Huoshizi to Bashan, 32.46°N, 106.30°E, 23.vii.2017, 1638 m alt., Jiangli Tan; 1 ♀ (NWUX), Shaanxi, Xunyangba, 33.55°N, 108.55°E, 1.vii.2017, 1481 m alt., Jiangli Tan; 2 ♀ (NWUX, RMNH), Shaanxi, Qiligou, Xunyangba, 507 m alt., 1.vii.2019, 32°91'N, 109°68'E, hand net, Tan JL; 3 ♀ (NWUX), Shaanxi, Huaishuzhuang Rev. St., Ziwuling NNR, Fuxian, Yanan, sweep net, 35.86°N, 108.74°E, 2–4.viii.2019, 1127–1271 m alt., Jiangli Tan; 5 ♀ (NWUX, RMNH), Shaanxi, Xialiangzhen, Zhashui, Shangluo, 33.67°N, 109.29°E, 23/28.viii.2016, 1059 m alt., Ruonan Zhang; 1 ♂ (NWUX), Shaanxi, Yuhua Palace, Yintai, Tongchuan, sweep net, 35.21°N, 108.54°E, 30.vii.2019, 1385 m alt., Jiangli Tan; 1 ♂ (NWUX), Shaanxi, Shuanglong, Xiangfang, Huangling, Yan’an, 35°35'28"N, 108°49'25"E, 1.viii.2019, 1102 m alt., Jiangli Tan; 3 ♀ + 2 ♂ (NWUX), Shaanxi, Miaojv, Liulin, Yaozhou, Tongchuan, sweep net, 35.60°N, 108.49°E, 27.vii.2019, alt. 934 m, Jiangli Tan; 1 ♀ + 1 ♂ (NWUX), Gansu, Lianjiabian, Taibai Heshui, sweep net, 36.06°N, 108.54°E, 8.viii.2019, alt. 1193 m, Ruonan Zhang.

**Figures 195–197.**
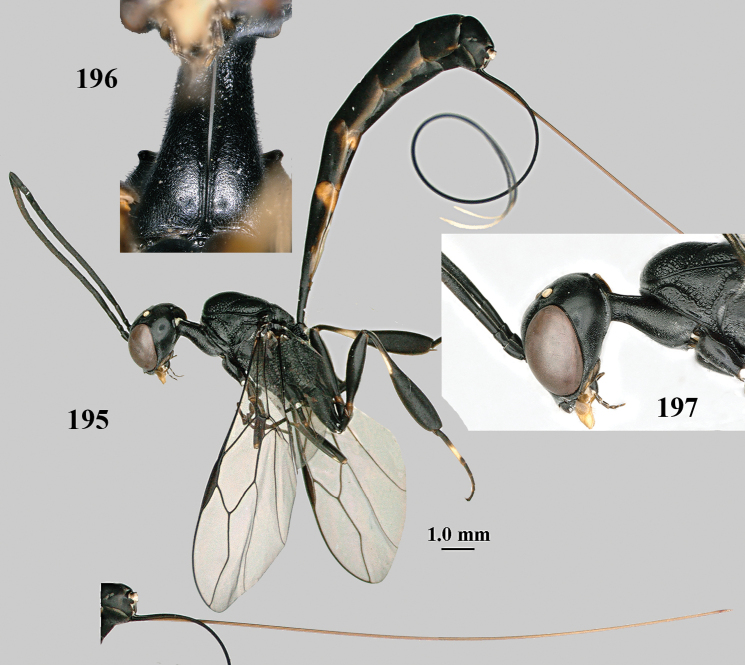
*Gasteruption oshimense* Watanabe, large female, Shaanxi **195** habitus lateral **196** pronotum ventral **197** head and pronotum lateral.

#### Distribution.

China (Gansu, Guizhou, Henan, Hunan, Jilin, Shaanxi), Japan. New for Gansu.

#### Notes.

One of the most common species in China; large specimens tend to have the head less narrowed, less shiny and more sculptured in dorsal view (Figs 204 and 207). The length of the white or ivory apical part of the ovipositor sheath is highly variable in this species; two females from Shangluo have it up to 3.3× as long as the hind basitarsus. The propleuron of *G. oshimense* is rather robust and 0.8–1.0× as long as mesoscutum in front of tegula.

**Figures 198–207.**
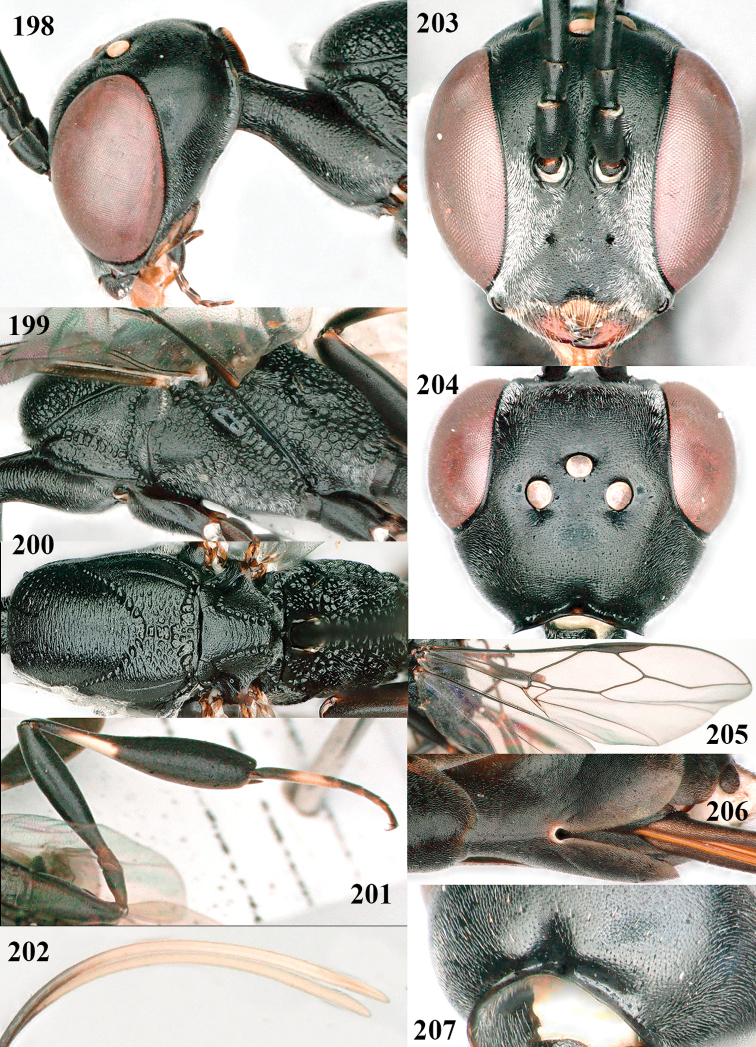
*Gasteruption oshimense* Watanabe, large female, Shaanxi **198** head lateral **199** mesosoma lateral **200** mesosoma dorsal **201** hind leg **202** apex of ovipositor sheath lateral **203** head anterior **204** head dorsal **205** wings **206** apex of metasoma ventral **207** medio-posterior depression of head latero-dorsal.

**Figures 208–212.**
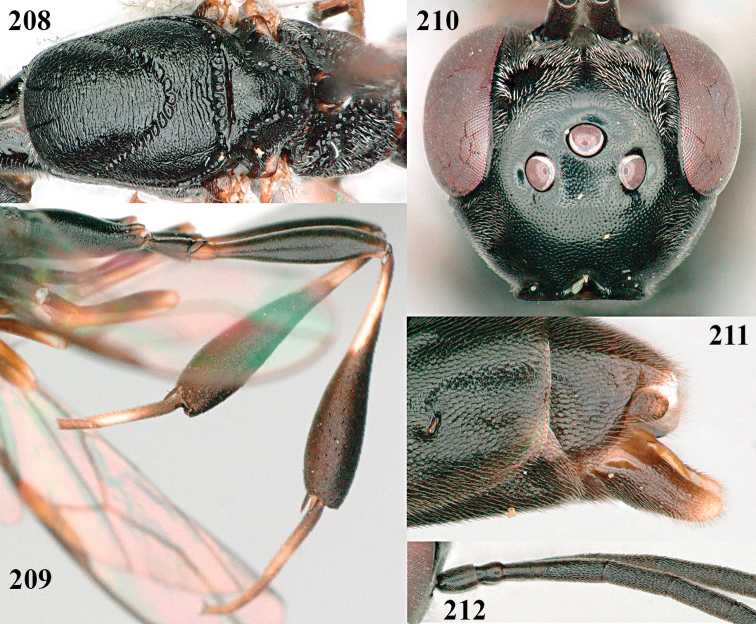
*Gasteruption oshimense* Watanabe, male, Shaanxi **208** mesosoma dorsal **209** hind leg **210** head dorsal **211** apex of metasoma lateral **212** basal antennal segments lateral.

### Gasteruption parvicollarium

Taxon classificationAnimaliaHymenopteraGasteruptiidae

Enderlein, 1913

A5E16A4D-8476-5D0F-B300-FB993D96A526

Figs 213–214 , 215–223 , 224–225 , 226–231

Gasteruption parvicollarium Enderlein, 1913: 323–324; Hedicke 1939: 27; Pasteels 1958: 183; Zhao et al. 2012: 68–72 (p.p.).

#### Additonal material.

2 ♀ (NWUX), NE China: Shaanxi, Xi’an, NWU Taibai campus, small garden, ca. 410 m alt., on flowers of *Cayratia japonica* (Thunberg), 24.v.2018, JL Tan; 2 ♀, id. but 13.vi.2020; 1 ♀, NWU Chang’an campus, on flowers of *Daucus carota* L. 30.vi.2020, JL Tan; 2 ♀ + 1 ♂ (RMNH), id., 27–30.vi.2017, C. v. Achterberg; 1 ♂ (NWUX), id., 7.vi.2018; 1 ♂ (NWUX), id., but 12.vi.2018, QQ Tan, RN Zhang; 1 ♀ + 1 ♂ (NWUX), NE China: Shaanxi, Lantian Ape Man Site, 34.10°N, 110.30°E, 8.vii.2017, 775 m alt., Qingqing Tan; 2 ♀ (NWUX), NW China: Shaanxi, Xi’an, Bailuyuan, Baqiao, 34.20°N, 109.12°E, 14.vii.2018, 687 m alt., Ruonan Zhang; 3 ♀ + 1 ♂ (NWUX, RMNH), S China: Fujian, Tianbaoyan, Yong-an, 25°53'42"N, 117°28'05"E, 22.v.–12.vi.2018/5–9.vi.2018/26.vi.2018/x–xii.2018, Mal. trap, 530 m alt., Lingfei Peng; 2 ♀ (NWUX), “S China: Fujian, Huboljiao, Nanping, 24°54'24"N, 117°12'52"E, 27.v.2018, Mal. trap, 300 m alt., Lingfei Peng, NWUX”.

**Figures 213–214.**
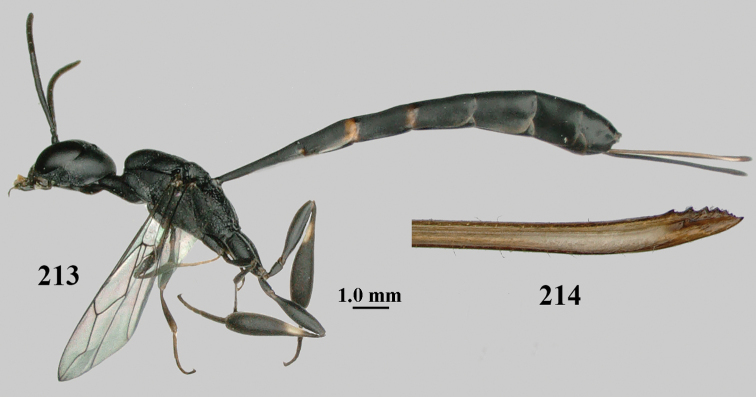
*Gasteruption parvicollarium* Enderlein, ♀, Shaanxi **213** habitus lateral **214** apex of ovipositor lateral.

#### Notes.

The proper recognition of a species depends a lot on the size and quality of the type series and *Gasteruption* species are no exception. It becomes very complicated if the holotype (and only available type specimen) is a deformed male from Taiwan as in the case of *G. parvicollarium* Enderlein. The illustrated associated female by Zhao et al. (2012) is very similar, but has a relatively short head compared to the holotype (which should be the other way around), the mandibles are yellowish (dark brown in the holotype) and the vertex in lateral view is less curved than in the holotype. Sometimes the solution is nearby; in the little garden of the old NWU Taibai campus (about 200 m away from the old city wall in the centre of a very large city), both sexes of a very similar *Gasteruption* species were collected on bush-killer, *Cayratia japonica* (Thunb.) Gagnep. (Vitaceae) in 2018. The flowers have easy reachable nectar and are visited by many Hymenoptera, including small *Hylaeus* bees, which may serve as hosts of *G. parvicollarium*. The female is more similar to the male holotype than the illustrated female by Zhao et al. (2012) and is obviously the real female of *G. parvicollarium*. Both sexes are illustrated in this paper; the related species with less bulging vertex is described as a new species (*G. granulatum* sp. nov.). The E. Palaearctic *G. parvicollarium* shares with the W. Palaearctic *G. variolosum* (Abeille de Perrin, 1879) the bulging vertex, the ovipositor sheath about 1.5× as long as the hind tibia and the elongate head. It differs by the finely coriaceous mesoscutum (reticulate-punctate in *G. variolosum*), the medium-sized dorsal teeth at the ovipositor apex (minute dorsal teeth), the narrower face (wider) and the coriaceous pronotal side (mainly rugulose).

**Figures 215–223.**
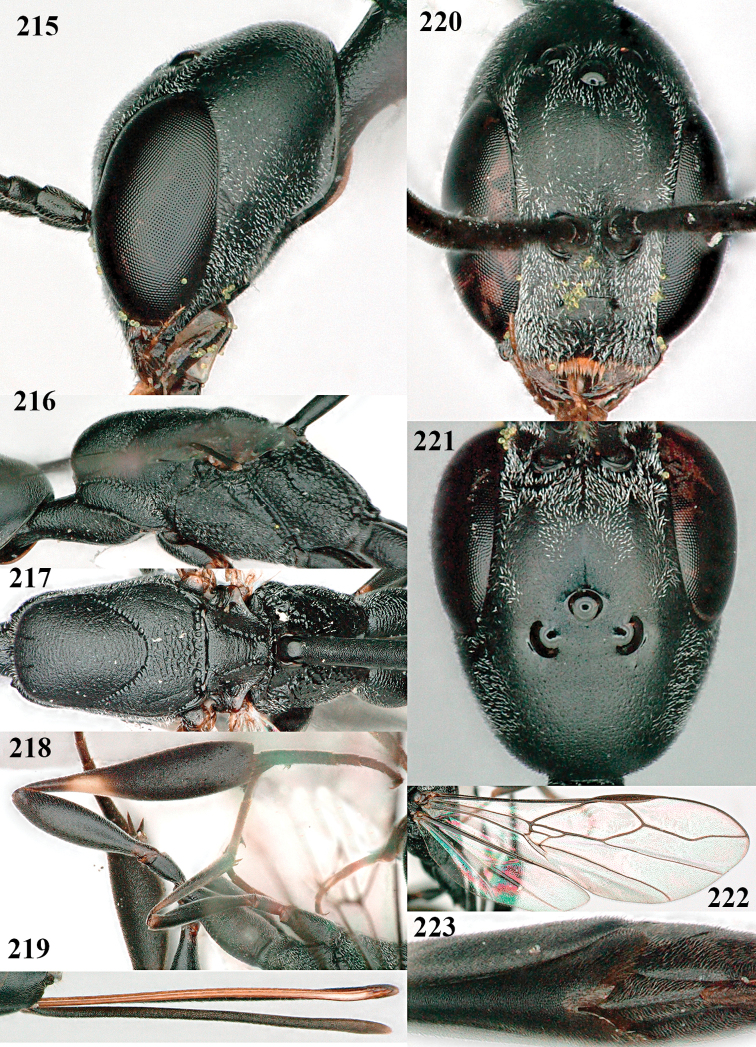
*Gasteruption parvicollarium* Enderlein, ♀, Shaanxi **215** head lateral **216** mesosoma lateral **217** mesosoma dorsal **218** hind leg **219** ovipositor and ovipositor sheath **220** head anterior **221** head dorsal **222** wings **223** apex of metasoma ventral.

**Figures 224–225.**
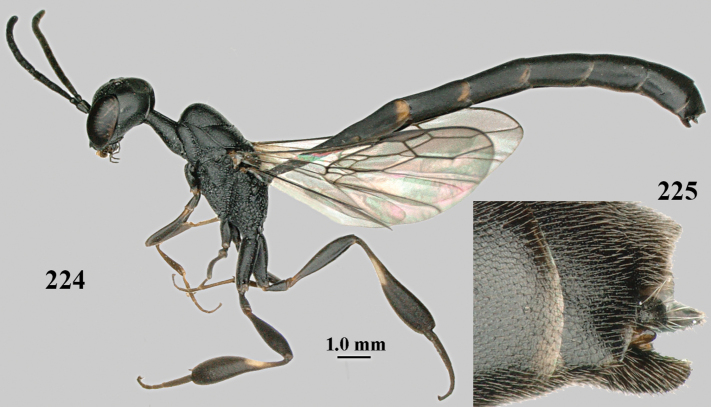
*Gasteruption parvicollarium* Enderlein, ♂, Shaanxi **224** habitus lateral **225** apex of metasoma lateral.

#### Distribution.

China (Fujian, Shaanxi, Taiwan). Other reports need reconfirmation. New for Fujian and Shaanxi.

**Figures 226–231.**
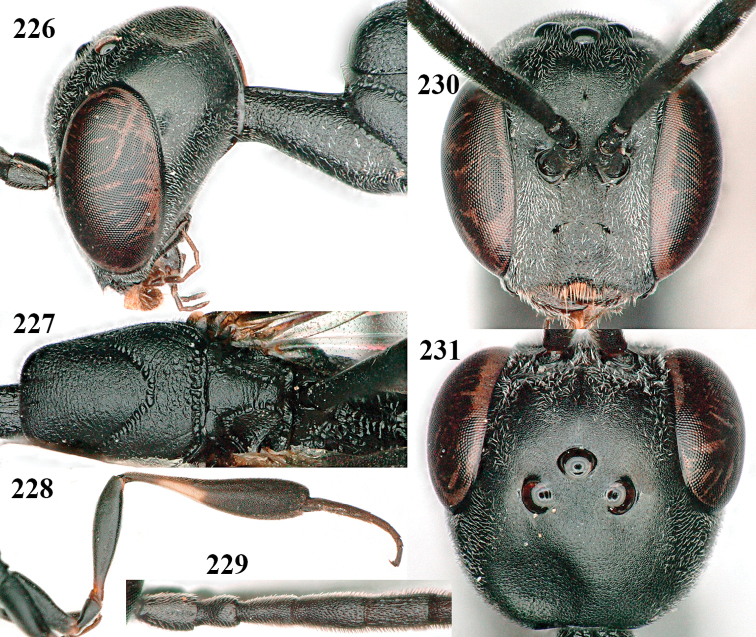
*Gasteruption parvicollarium* Enderlein, ♂, Shaanxi **226** head lateral **227** mesosoma dorsal **228** hind leg lateral **229** basal antennal segments lateral **230** head anterior **231** head dorsal.

### Gasteruption pedion

Taxon classificationAnimaliaHymenopteraGasteruptiidae

Tan & van Achterberg sp. nov.

7C21076F-8285-5D96-98B2-4D5298FEE1B3

http://zoobank.org/6D3061AF-E566-4103-9572-D679AF27194F

Figs 232–234 , 235–245 , 246–252

#### Type material.

***Holotype*,** ♀ (NWUX), “NW China: Shaanxi, Foping, Panda Valley, 1411 m alt., black Mal[aise] trap, 33.67°N, 107.97°E, 1.vii.–18.viii.2016, Jiangli Tan, NWUX”. ***Paratypes***: 1 ♀ + 1 ♂ (NWUX, RMNH), “NW China: Shaanxi, Liangfengya, Foping, 33.09°N, 107.90°E, 28.iv.–9.vi.2019, 1729 m alt., w[hite]/[y]ellow Mal[aise] trap, Qingqing Tan, NWUX”; 2 ♀ + 1 ♂ (NWUX, RMNH), id., but 9.vi-22.viii.2019; 1 ♀ (SCAU), “[S China:] Yunnan, Xianggelila, Gaoshan Botanical Garden, 27°53'47"N, 99°38'22"E, M[alaise]T[rap], 3–27.viii.2017, Jie Zeng”; 1 ♂ (SCAU), “[N China:] Jiangsu, Nanjing, Xianlin, Mt. Duo, 32°6'51"N, 118°54'43"E, 9–15.iv.2012, M[alaise]T[rap], Jie Zhao”.

**Figures 232–234.**
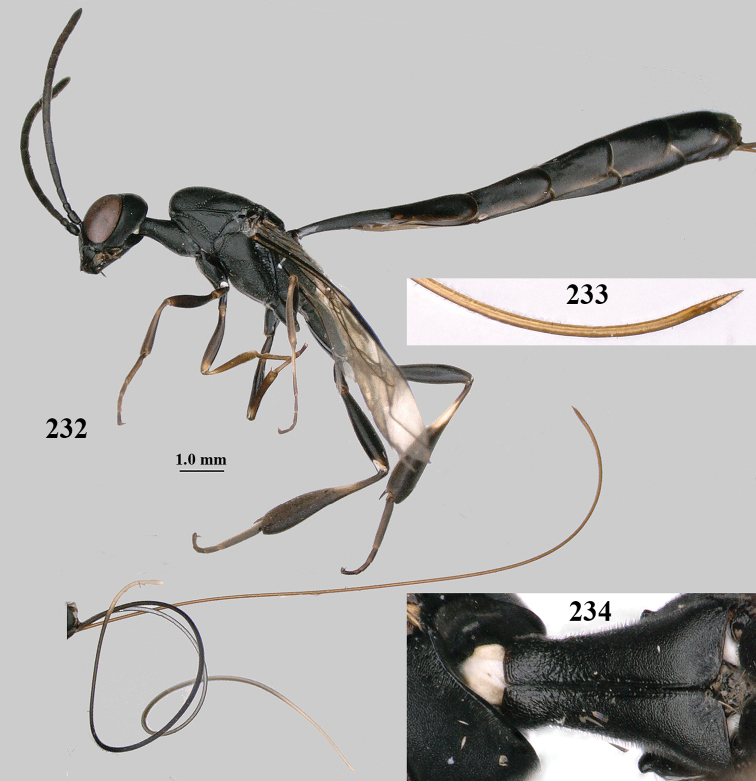
*Gasteruption pedion* Tan & van Achterberg, sp. nov., female, holotype **232** habitus lateral **233** apex of ovipositor lateral **234** propleuron ventral.

#### Diagnosis.

Head gradually narrowed in dorsal view and with satin sheen (Fig. 241); vertex in lateral view more or less above level of ocelli and evenly convex in front of occipital carina; mandibular condylus near lower level of eyes; occipital carina non-lamelliform medio-dorsally; fourth antennal segment of ♀ 1.5–1.6× as long as third segment; third antennal segment of ♂ 1.4–1.5× as long as second segment; clypeus with obsolescent depression; propleuron 0.8–0.9× as long as mesoscutum in front of tegula; pronotal side entirely finely coriaceous ventrally; mesoscutum very finely coriaceous between sparse punctulation; notauli only crenulate posteriorly and distinctly impressed and anteriorly wider and moderately crenulate; hind femur and tibia slender; ovipositor sheath 0.9–1.1× as long as body; apical white part of ovipositor sheath 2.1–2.6× as long as hind basitarsus; apical sternite of ♂ entirely dark brown and paramere densely whitish setose, with its apex ivory or pale yellowish (Fig. 247).

**Figures 235–245.**
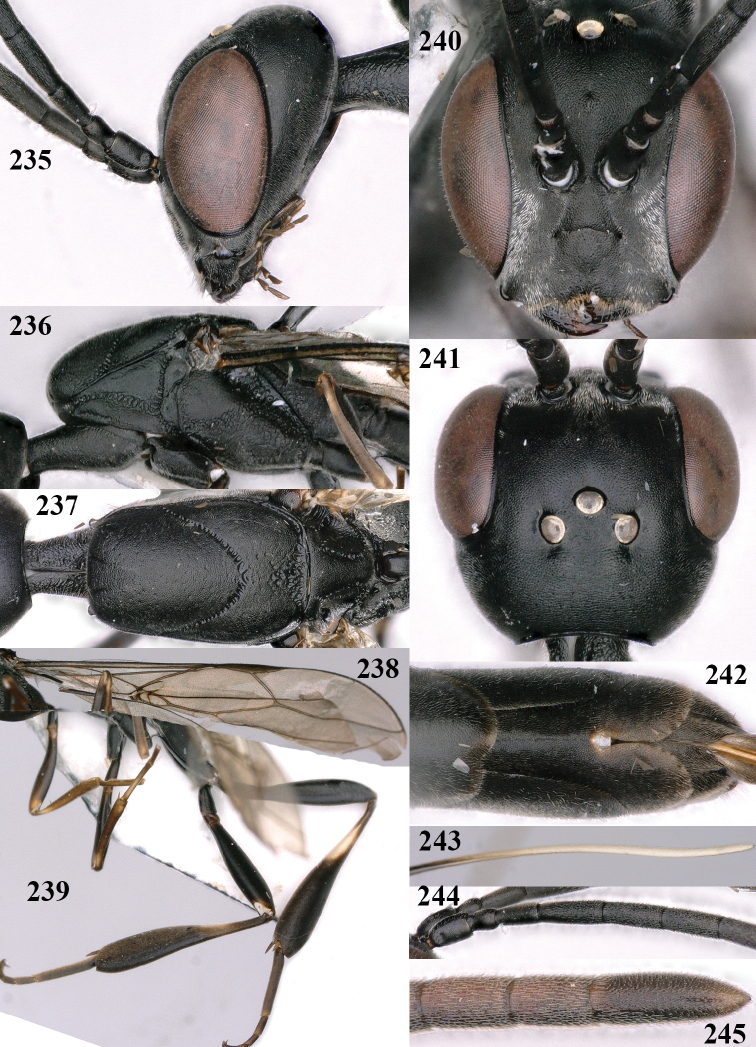
*Gasteruption pedion* Tan & van Achterberg, sp. nov., female, holotype **235** head lateral **236** mesosoma lateral **237** mesosoma dorsal **238** fore wing **239** hind leg **240** head anterior **241** head dorsal **242** apex of metasoma ventral **243** apex of ovipositor sheath **244** base of antenna **245** apex of antenna.

Easily confused with *G. sinepunctatum* Zhao, van Achterberg & Xu, 2012, but the new species has the mesoscutal lobes flattened and very finely coriaceous without transverse elements (mesoscutal lobes bumpy and sculpture with very fine transverse elements in *G. sinepunctatum*), the vertex rather matt and densely finely sculptured with very fine transverse rugulae (rather sparsely to densely punctulate and rather shiny), the mandible black (largely brownish-yellow), the metasoma of ♀ black ventrally (largely yellowish-brown) and the fore coxa black (dark brown).

#### Description.

Holotype, female, length of body 15.0 mm, of fore wing 7.8 mm.

***Head*.** Vertex and frons with rather matt, very finely and densely coriaceous, on vertex mixed with very fine transverse elements; vertex moderately convex in lateral view (Fig. 235) and without a depression medio-posteriorly; head gradually contracted behind eyes in dorsal view and temples curved (Fig. 241); temple 0.6× as long as eye in dorsal view; fourth antennal segment 1.5× as long as third segment and as long as second and third segments combined, fifth antennal segment 1.4× as long as third segment, third antennal segment twice as long as second segment (Fig. 244); occipital carina narrow and non-lamelliform medio-dorsally (Fig. 235); OOL slightly longer than POL and 1.7× as long as diameter of posterior ocellus; face wide, 3.8× as broad as high (Fig. 240); combined height of eye and malar space 1.6× minimum width of face; malar space slightly protruding below lower level of eyes (Fig. 240), its minimum width 0.2× basal width of mandible and area behind incision nearly triangular (Fig. 235); clypeus only medio-ventrally shallowly depressed and latero-ventral corners rather protruding (Fig. 240); eye with numerous short setae.

***Mesosoma*.** Length of mesosoma twice its height; propleuron rather robust and 0.9× as long as mesoscutum in front of tegula, in ventral view rather robust and less narrowed than in *G. sinepunctatum*; pronotal side entirely granulate-coriaceous, except for wide crenulated grooves and sparsely setose, with obtuse and rather large lobe-shaped tooth antero-ventrally (Figs 236 and 237); antesternal carina narrow and hardly lamelliform; mesosternal sulcus wide and deep, slightly widened posteriorly and coarsely crenulate; mesoscutum and scutellum rather matt, very densely and very finely granulate-coriaceous and with some fine superficial punctures (Fig. 237); propodeum reticulate-rugose and without median smooth band or carina. ***Wings*.** First discal cell wide, parallel-sided and with outer posterior corner rounded and with vein 3-CU1 near its apical third (Fig. 238). ***Legs*.** Hind coxa finely granulate-coriaceous, dorsally with superficial transverse rugulae; length of hind femur, tibia and basitarsus 4.4, 5.5 and 6.5× their width, respectively; hind tibia slightly inflated (Fig. 239); middle tarsus 1.2× as long as middle tibia; middle femur subparallel-sided and more slender than fore femur.

***Metasoma*.** Ovipositor sheath 13.6 mm, 0.9× as long as body, 1.3× as long as metasoma and 4.3× as long as hind tibia; ovipositor sheath with dense cover of very fine adpressed setae, its white apical part (becoming ivory more basally) 2.6× as long as hind basitarsus; apical half of hypopygium emarginate medio-posteriorly.

***Colour*.** Black (including mandible); subapically antenna somewhat brownish ventrally; tegula, legs (but coxae black, hind tibia with large ivory ventro-basal patch, ivory basal patch of fore and middle tibia and middle basitarsus (except apex)), veins and pterostigma dark brown; wing membrane slightly brownish; apex of ovipositor white (Fig. 243).

**Male.** Very similar to female (including fine sculpture of mesoscutum, but usually somewhat coarser (Fig. 249) and with very fine aciculae, especially of small males), but head shorter in dorsal view; third antennal segment 1.4–1.5× as long as second segment; fourth antennal segment 1.8–2.3× as long as third segment and 1.1–1.4× as long as second and third segments combined, fifth antennal segment 1.9–2.3× as long as third segment (Fig. 251); apical sternite entirely dark brown; paramere densely whitish setose and its apex ivory or pale yellowish (Fig. 247).

**Figures 246–252.**
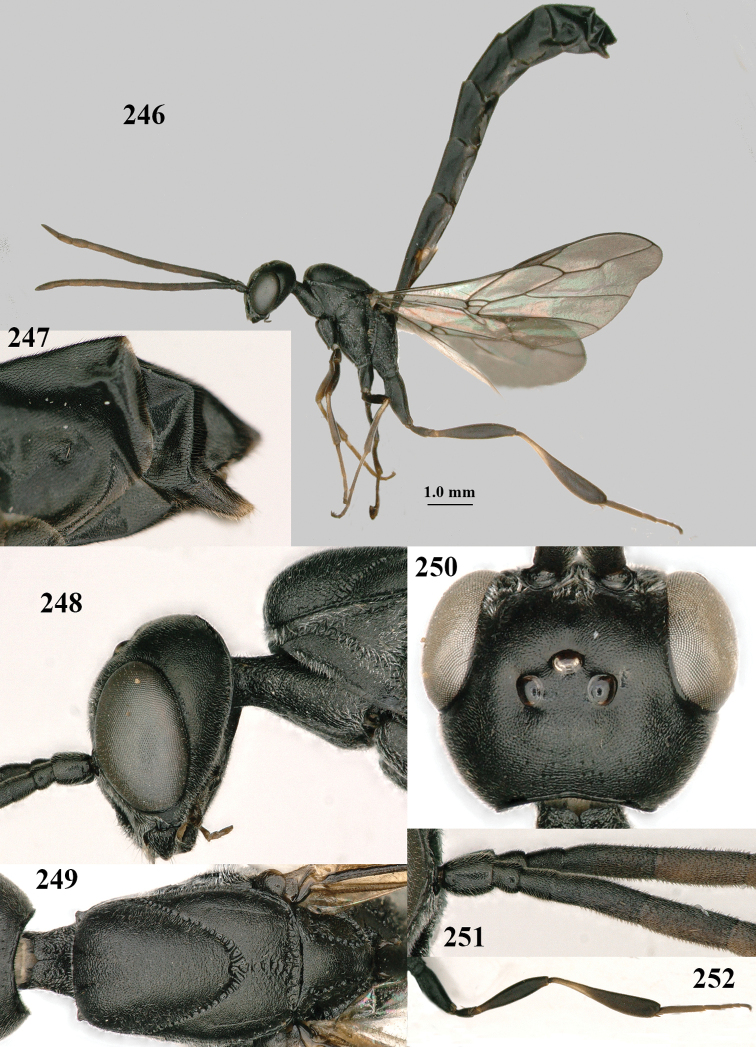
*Gasteruption pedion* Tan & van Achterberg, sp. nov., male, paratype **246** habitus lateral **247** apex of metasoma lateral **248** head lateral **249** mesosoma dorsal **250** head dorsal **251** base of antenna **252** hind leg.

#### Variations.

Body length of ♀ 12.3–15.7 mm, of ♂ 12.2–14.7 mm; fourth antennal segment of ♀ 1.5–1.6× as long as third segment; ovipositor sheath 0.9–1.1× as long as body; apical white part of ovipositor sheath 2.1–2.6× as long as hind basitarsus; propleuron 0.8–0.9× as long as mesoscutum in front of tegula.

#### Distribution.

China (Jiangsu, Shaanxi, Yunnan).

#### Etymology.

From “pedion” (Greek for “flat, plain”), because of the flat and evenly coriaceous mesoscutum.

### Gasteruption reductum

Taxon classificationAnimaliaHymenopteraGasteruptiidae

Tan & van Achterberg sp. nov.

614D7FD0-8ACA-56C3-B938-DB85C23AD334

http://zoobank.org/B044A0C6-1FC8-4BC1-9981-363BF4586C6D

Figs 253 , 254–263 , 264–271

#### Type material.

***Holotype*,** ♀ (NWUX), “NW China: Shaanxi, [Xi’an,] Baolongyu, Ziwuzhen, 34.02°N, 108.91°E, 10.iii.–27.v.2018, y[ellow] Mal[aise] trap, 948 m alt., QQ Tan, RN Zhang, NWUX”. ***Paratypes***: 3 ♀ + 1 ♂ (NWUX, RMNH), “Shaanxi, Xiangfang, Shuanglong, Huangling, Yan’an, 35.63°N, 108.87°E, 4.viii.2019, 1271 m alt., Jiangli Tan, NWUX”; 1 ♀ (NWUX), id., but 31.vii.2019, 1007 m alt.; 1 ♀ (RMNH), “NW China: Shaanxi, Huaishuzhuang Rev. St., Ziwuling NNR, Fuxian, Yanan, sweep net, 35.86°N, 108.74°E, 2.viii.2019, 1127 m alt., Jiangli Tan, NWUX”; 1 ♀ (SCAU), “[NE China:] Hebei, Chicheng Co., Songshan NNR, 1440 m alt., 40.53067°N, 115.74772°E, 11–18.vii.2012, M[alaise]T[rap], Changqing Xia”.

**Figure 253.**
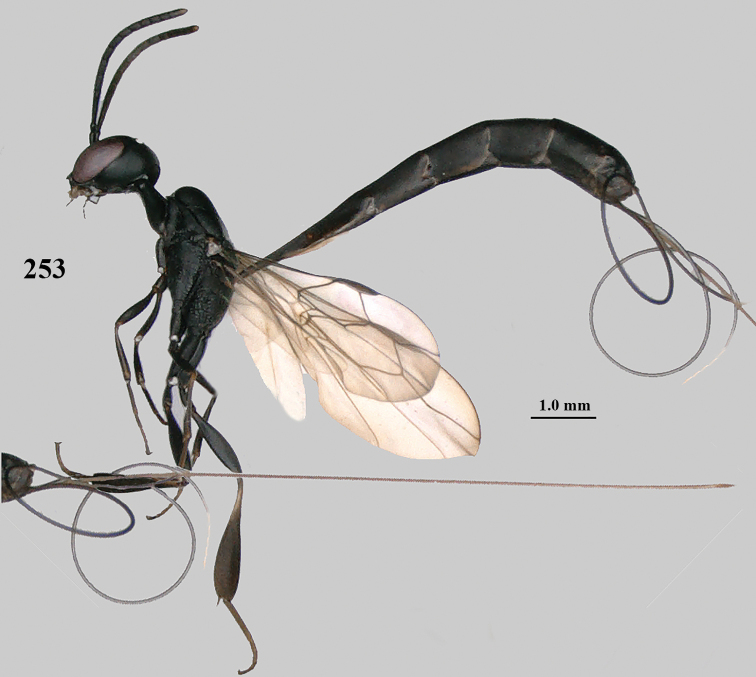
*Gasteruption reductum* Tan & van Achterberg, sp. nov., female, holotype, habitus lateral.

#### Diagnosis.

Head in dorsal view distinctly narrowed, moderately convex and medio-posteriorly flat, without a depression in front of occipital carina; mandibular condylus near lower level of eyes; temple dorsally dull and finely coriaceous; fourth antennal segment of ♀ 1.2–1.3× as long as third segment; third antennal segment of ♂ 1.4× as long as second segment; occipital carina non-lamelliform medio-dorsally propleuron; 0.8–0.9× as long as mesoscutum in front of tegula; notauli narrow, finely crenulate and posteriorly reduced and with transverse rugae; mesoscutum rather flat, with satin sheen and without rugae or punctures in lateral view; mesoscutum mainly very finely coriaceous mixed with very fine transverse rugulae, but medio-posteriorly with distinct transverse rugae; hind femur and tibia slender; ovipositor sheath 1.0–1.1× as long as body; apical white part of ovipositor sheath 1.2–2.5× as long as hind basitarsus; apical sternite of ♂ entirely dark brown and paramere densely whitish setose, with its apex dark brown.

**Figures 254–263.**
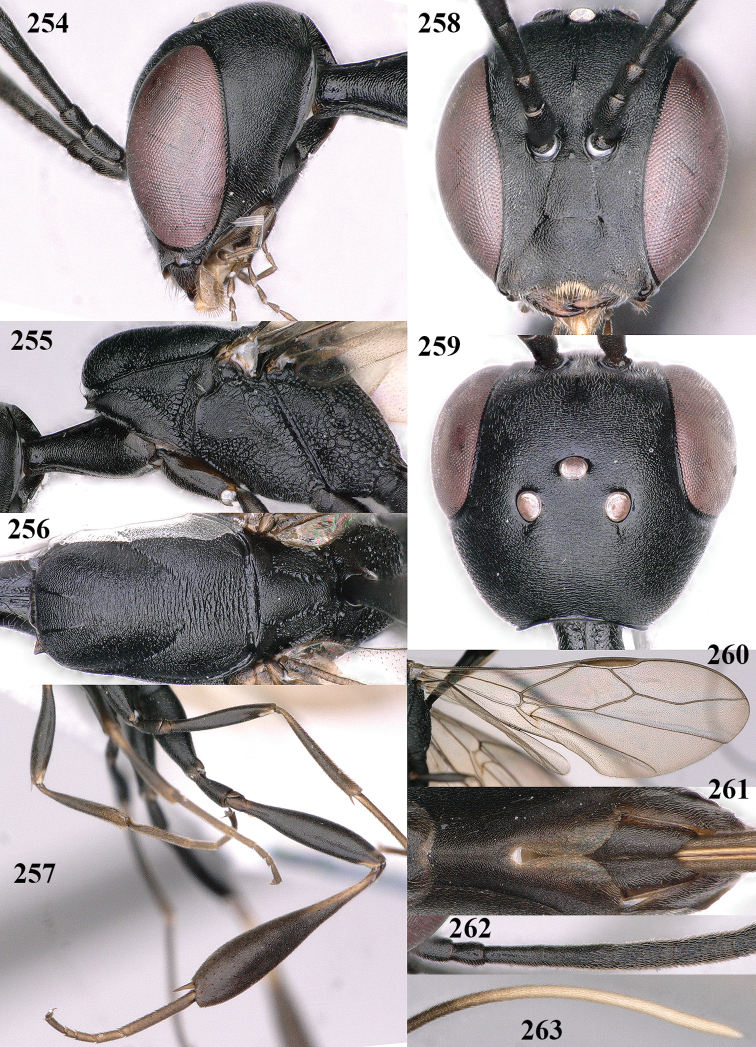
*Gasteruption reductum* Tan & van Achterberg, sp. nov., female, holotype **254** head lateral **255** mesosoma lateral **256** mesosoma dorsal **257** hind leg **258** head anterior **259** head dorsal **260** fore wing **261** apex of metasoma ventral **262** base of antenna **263** apex of ovipositor sheath.

*Gasteruption reductum* shares with *G. graciloides* van Achterberg, 2019, from Far East Russia, the peculiar sculpture of the mesoscutum and the slender body, but the new species has the notauli narrow, finely crenulate and posteriorly reduced and with transverse rugae (notauli medium-sized, moderately crenulate and posteriorly distinctly impressed and only crenulate in *G. graciloides*) and the vertex distinctly protruding above level of ocelli (hardly protruding above level ocelli in *G. graciloides*). The new species differs from *G. pedion* sp. nov. mainly by the reduced notauli (distinctly impressed in *G. pedion*), the propleuron distinctly narrowed anteriorly in ventral view (hardly narrowed), the very finely transversely rugulose mesoscutum (only very finely granulate-coriaceous) and the small ocelli (larger).

#### Description.

Holotype, female, length of body 9.1 mm, of fore wing 4.3 mm.

***Head*.** Frons very finely coriaceous and with satin sheen; vertex very finely coriaceous with very fine transverse rugulae, moderately convex and medio-posteriorly flat, without a depression; head rather gradually contracted behind eyes in dorsal view and temples slightly rounded (Fig. 259); temple 0.8× as long as eye in dorsal view; fourth antennal segment 1.3× as long as third segment and 0.8× as long as second and third segments combined, fifth antennal segment as long as third segment, third antennal segment 1.8× as long as second segment (Fig. 262); occipital carina narrow and non-lamelliform medio-dorsally (Fig. 259); OOL 1.4× as long as diameter of posterior ocellus and POL about 1.5× OOL; face 2.4× as broad as high (Fig. 258); combined height of eye and malar space 1.9× minimum width of face; malar space not protruding below lower level of eyes (Fig. 258), its minimum width 0.2× basal width of mandible and area behind incision nearly triangular and elongate (Fig. 254); clypeus only medio-ventrally shallowly depressed and ventro-lateral corners protruding (Fig. 258); eye virtually glabrous.

***Mesosoma*.** Length of mesosoma 1.9× its height; propleuron moderately robust, shiny and 0.9× as long as mesoscutum in front of tegula; pronotal side granulate-coriaceous, except for wide crenulate-rugose grooves and sparsely setose, with distinct acute tooth antero-ventrally (Figs 255 and 256); antesternal carina narrow and non-lamelliform; mesosternal sulcus very wide and coarsely crenulate; notauli narrow, finely crenulate and posteriorly reduced and with transverse rugae; mesoscutum rather flat, with satin sheen and without rugae or punctures in lateral view; dorsally middle lobe of mesoscutum very finely coriaceous and mixed with very fine transverse rugulae, becoming stronger posteriorly; lateral lobes of mesoscutum mainly very finely coriaceous, but medio-posteriorly with distinct transverse rugae (Figs 255 and 256); scutellum rather matt and very finely and densely coriaceous (Fig. 256; propodeum irregular reticulate-rugose, rather shiny and without median carina or wide smooth stripe. ***Wings*.** First discal cell narrow, slightly narrowed apically and with outer posterior corner obsolescent and with vein 3-CU1 near its apical fifth (Fig. 260). ***Legs*.** Hind coxa finely coriaceous, but dorsally irregularly transversely rugulose; length of hind femur, tibia and basitarsus 4.6, 4.9 and 7.4× their width, respectively; hind tibia moderately inflated (Fig. 257); middle tarsus 1.3× as long as middle tibia; middle femur subparallel-sided and distinctly more slender than fore femur.

***Metasoma*.** Ovipositor sheath 9.7 mm, 1.1× as long as body, 1.5× as long as metasoma and 5.5× as long as hind tibia; ovipositor sheath with dense cover of fine adpressed setae, its white apical part 1.7× longer than hind basitarsus; apical half of hypopygium emarginate medially.

***Colour*.** Black; apex of apical antennal segment brown; basal half of mandible dark brown and apical half dark reddish-brown; tegulum, humeral plate, veins and pterostigma largely brown; fore and middle tibiae (but basally and apically somewhat paler) and tarsi, trochantelli and hind tarsus dark brown; hind tibia baso-ventrally with elongate ivory patch; hind tibial spurs brown; wing membrane slightly brownish; ivory or whitish apex of ovipositor sheath basally brownish and remainder of sheath dark brown (Fig. 263).

**Male.** Similar to female (including very fine transverse sculpture of mesoscutum: Fig. 267), but head shorter in dorsal view and notauli posteriorly impressed and finely crenulate (Fig. 267), without transverse rugae; third antennal segment 1.4× as long as second segment; fourth antennal segment nearly twice as long as third segment and 1.1× as long as second and third segments combined and fifth antennal segment twice as long as third segment (Fig. 271); apical sternite entirely dark brown; paramere densely whitish setose and its apex dark brown (Fig. 269).

**Figures 264–271.**
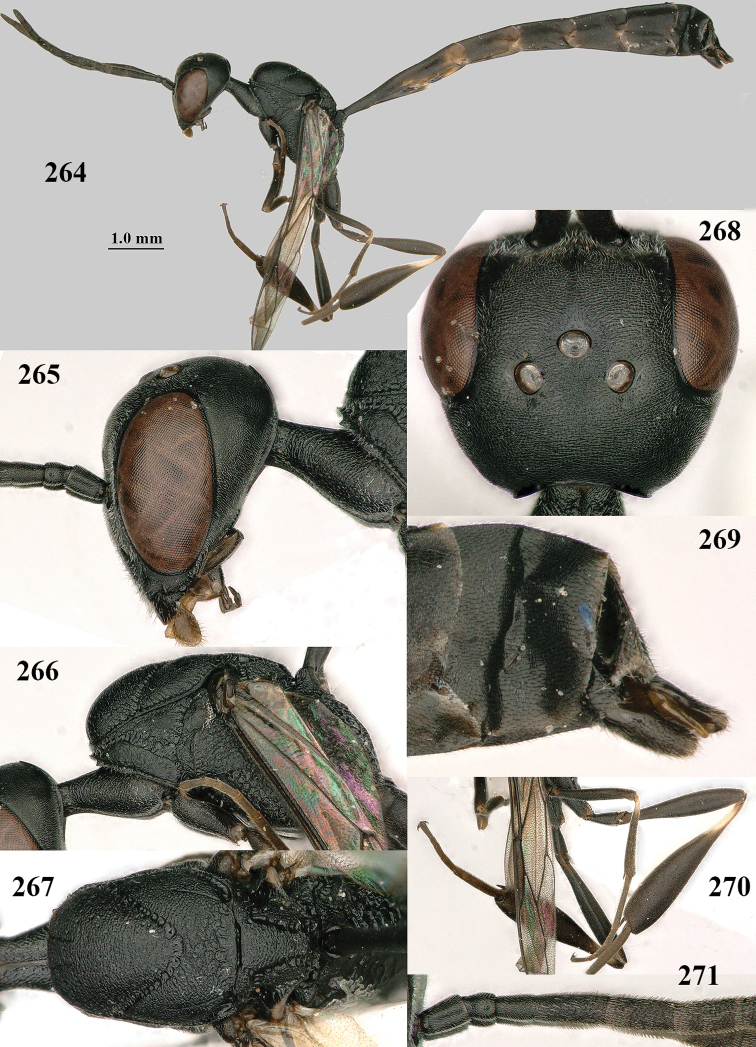
*Gasteruption reductum* Tan & van Achterberg, sp. nov., male, paratype **264** habitus lateral **265** head lateral **266** mesosoma lateral **267** mesosoma dorsal **268** head dorsal **269** apex of metasoma lateral **270** hind leg **271** base of antenna.

#### Variations.

Body length of ♀ 9.1–11.5 mm, of ♂ 9.5 mm; fourth antennal segment of ♀ 1.2–1.3× as long as third segment; ovipositor sheath 1.0–1.1× as long as body; apical white part of ovipositor sheath 1.2–2.5× as long as hind basitarsus; propleuron 0.8–0.9× as long as mesoscutum in front of tegula.

#### Distribution.

China (Shaanxi, Hebei).

#### Etymology.

Name derived from “reductus” (Latin for “withdrawn”) because of the narrow and posteriorly reduced notauli.

### Gasteruption sinarum

Taxon classificationAnimaliaHymenopteraGasteruptiidae

Kieffer, 1911

52C34F86-C371-52AB-8A2F-C64A16C7C67B

Figs 272–275 , 276–284 , 285–291

Gasteruption sinarum Kieffer, 1911: 205–206, 1912: 229, 264; Hedicke 1939: 21; Zhao et al. 2012: 80–85; van Achterberg et al. 2019: 6.
Gasteruption sinense Kieffer, 1924: 77–78; Hedicke 1939: 21 (synonymised with G. sinarum by Zhao et al. (2012)).

#### Additional material.

1 ♀ (NWUX), “NW China: Shaanxi, Bailuyuan, Baqiao, Xi’an, 34.20°N, 109.12°E, 14.vii.2018, 687 m alt., Ruonan Zhang, NWUX”; 1 ♂ (NWUX), “China: Inner Mongolia, Keshiketeng, Chifeng, Dalinor to Baiyin Ovoo, 43.41°N, 116.68°E, 17.vii.2016, ca. 1360 m alt., Jiangli Tan, NWUX”.

**Figures 272–275.**
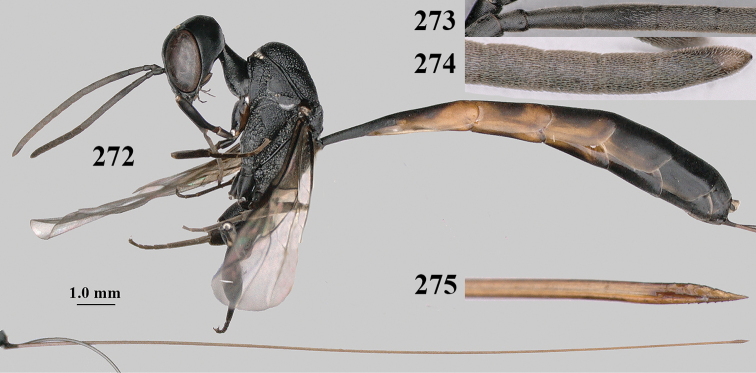
*Gasteruption sinarum* Kieffer, female, Shaanxi **272** habitus lateral **273** base of antenna **274** apex of antenna **275** apex of ovipositor lateral.

#### Distribution.

China (Anhui, Beijing, Guangdong, Guangxi, Guizhou Henan, Hubei, Hunan, Inner Mongolia, Jiangsu, Liaoning, Ningxia, Shaanxi, Shandong, Shanghai, Tianjin, Zhejiang). New for Shaanxi.

**Figures 276–284.**
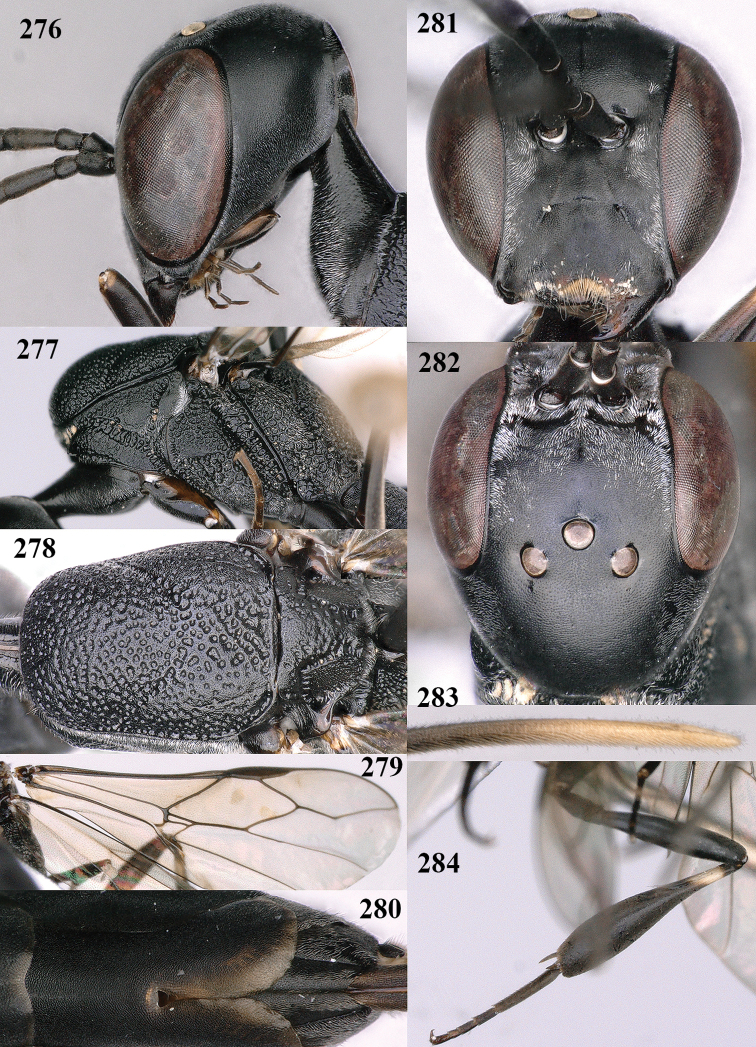
*Gasteruption sinarum* Kieffer, female, Shaanxi **276** head lateral **277** mesosoma lateral **278** mesosoma dorsal **279** wings **280** apex of metasoma ventral **281** head anterior **282** head dorsal **283** apex of ovipositor sheath **284** hind leg.

**Figures 285–291.**
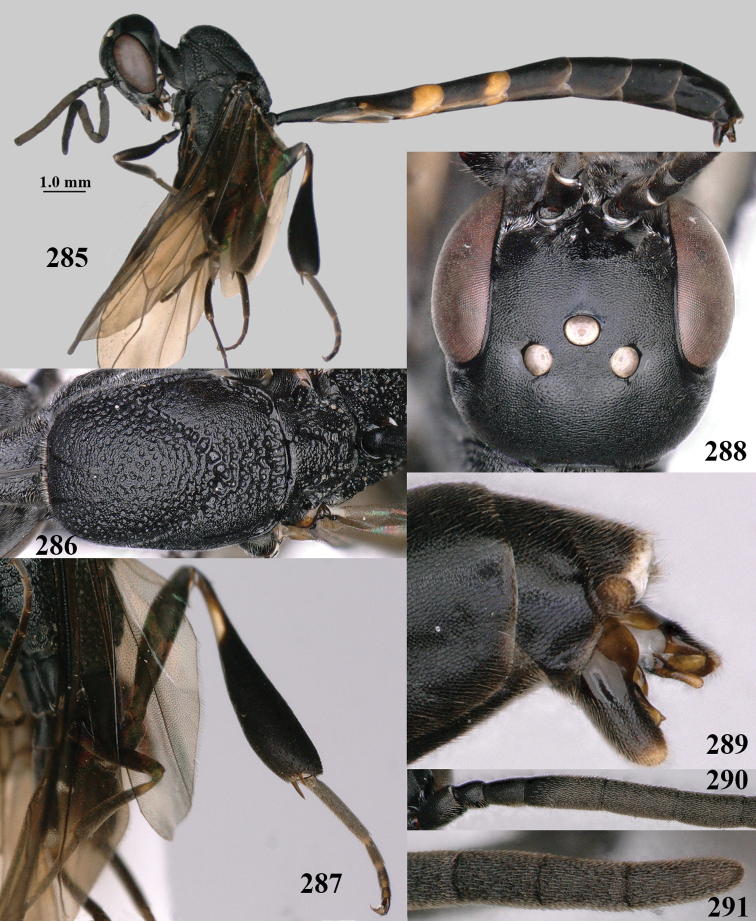
*Gasteruption sinarum* Kieffer, male, Shaanxi **285** habitus lateral **286** mesosoma dorsal **287** hind leg **288** head dorsal **289** apex of metasoma lateral **290** base of antenna **291** apex of antenna.

### Gasteruption sinepunctatum

Taxon classificationAnimaliaHymenopteraGasteruptiidae

Zhao, van Achterberg & Xu, 2012

5167C793-25DD-51AD-A3F6-F4921AE3AD68

Figs 292–298

Gasteruption sinepunctatum Zhao, van Achterberg & Xu, 2012: 85; Tan et al. 2016: 108–109.

#### Additional material.

1 ♀ (NWUX), NW China: Shaanxi, Foping, Panda Valley, 18.viii.2016, Jiangli Tan; 1 ♀ + 1 ♂ (NWUX, RMNH), id., 1411 m alt., black Malaise trap, 1.vii.–18.viii.2016; 1 ♂ (NWUX), Xunyangba, Ningshaan, 20.v.–23.vi.2016, green Malaise trap, 1481 m alt.; 1 ♀ (NWUX), id., 17.viii.–3.x.2016; 1 ♂ (NWUX), id., but yellow & green Malaise trap, 1.vii.–17.viii.2016; 1 ♀ (NWUX), id., 1.vii.2018, JL Tan; 2 ♂ (NWUX, RMNH), Shaanxi, Ningqiang, Hanzhong, Tiankeng, Chanjiyan, 32.46°N, 106.30°E, 25.vi–22.vii.2017, b[lack] Malaise trap, 1638 m alt., Jiangli Tan; 1 ♀ (NWUX), Shaanxi, Xunyangba, Ningshan, 1.vii.2018, ca. 1480 m alt., 33°54'N, 108°55'E, Jiangli Tan.

**Figures 292–298.**
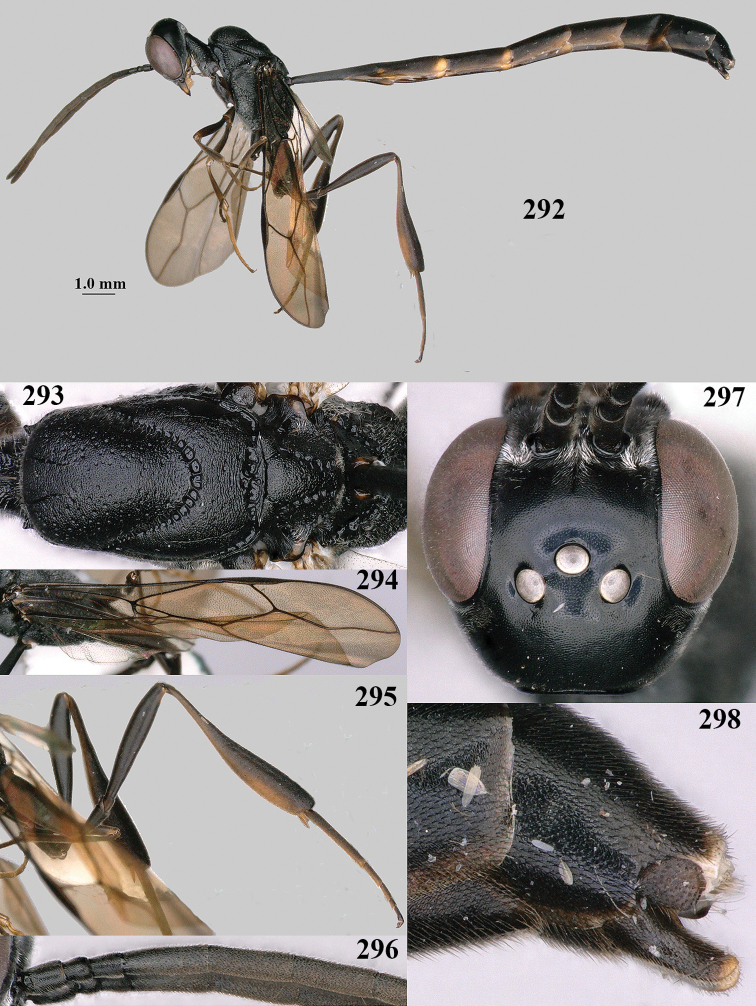
*Gasteruption sinepunctatum* Zhao, van Achterberg & Xu, male, Shaanxi **292** habitus lateral **293** mesosoma dorsal **294** fore wing **295** hind leg lateral **296** basal antennal segments lateral **297** head dorsal **298** apex of metasoma lateral.

#### Notes.

*Gasteruption sinepunctatum* Zhao, van Achterberg & Xu, 2012, is a large (about 15 mm body length or more) species described from C. China (Zhejiang) with paratypes from Taiwan, Jilin and Tibet. A large (13 mm body length) male from Taiwan has been associated with this species (Zhao et al. 2012). Recent collections in Daba and Qinling Mts shows that, in Shaanxi, there is a species with large males recognisable by the relatively long fourth antennal segment (2.7–3.1× as long as third segment (Fig. 296); 3.5× in paratype from Taiwan) and the distinctly protruding middle lobe of the mesoscutum (Figs 292 and 293). Both characters (together with the superficial and very fine sculpture of the mesoscutum) connects it with *Gasteruption sinepunctatum*, a species of which the female was reported from Shaanxi by Tan et al. (2016); the male is illustrated here to allow a better recognition.

#### Distribution.

China (Jilin, Shaanxi, Taiwan, Tibet, Zhejiang).

### Gasteruption subtile

Taxon classificationAnimaliaHymenopteraGasteruptiidae

(Thomson, 1883)

3970A607-1110-5A24-A18F-F69C5CBB3A17

Figs 299–307

Foenus subtilis Thomson, 1883: 847.
Gasteruption subtile ; Schletterer 1889: 396, 425; Dalla Torre 1902: 1072; Szépligeti 1903: 370; Kieffer 1912: 263; Maidl 1923: 35; Schmiedeknecht 1930: 377; Roman 1932: 8; Hedicke 1939: 21; Ferrière 1946: 237, 239, 244; Hellén 1950: 4; Šedivý 1958: 35, 37, 42; Györfi and Bajári 1962: 43, 50; Hedqvist 1973: 186 (lectotype designation); Madl 1989a: 161, 1989b: 44; Scaramozzino 1995: 3; Pagliano and Scaramozzino 2000: 13, 19, 33; Saure 2001: 29; van Achterberg 2013: fig. 185.
Gasteruption kriechbaumeri Schletterer, 1889: 384, 389, 395, 396, 426; Dalla Torre 1902: 1068; Szépligeti 1903: 370; Kieffer 1912: 267; Schmiedeknecht 1930: 378, 382; Hedicke 1939: 15; Ferrière 1946: 237, 244; Györfi and Bajári 1962: 43, 50; Schmidt 1969: 294; Madl 1989a: 161, 1989b: 44; Wall 1994: 149. Synonymised with G. subtile (Thomson) by Madl (1989a).
Gasteruption sabulosum Schletterer, 1889: 390, 396, 423; Dalla Torre 1902: 1072; Szépligeti 1903: 370; Kieffer 1912: 264; Maidl 1923: 35; Schmiedeknecht 1930: 377; Hedicke 1939: 20; Ferrière 1946: 244; Madl 1989: 161; Wall 1994: 149. Synonymised with G. kriechbaumeri Schletterer by Ferrière (1946) and with G. subtile (Thomson) by Madl (1989a).
Gasteruption poecilothecus Kieffer, 1911: 205.
Gasteruption poecilothecum ; Zhao et al. 2012: 73–75; Tan et al. 2016: 69, 84; van Achterberg et al. 2019: 6; van Achterberg 2019a: 7 (as synonym of G. subtile (Thomson)), 2019b: 22 (id.).
Gasteruption rossicum Semenov Tian-Shanskij & Kostylev, 1928: 89; Hedicke 1939: 20; Kozlov 1974: 76; Madl 1989a: 161; Wall 1994: 149. Synonymised with G. subtile (Thomson) by Kozlov (1974).

#### Type material.

***Lectotype*** of *G. subtile* ♀ (ZIL) from Sweden, “Norl” [= Norrland], “*subtilis*”, “Lectotypus *Foenus subtilis* Thoms., ♀, K.-J. Hedqvist, det. 1972”. ***Holotype*** of *G. poecilothecum*, ♀ (BMNH), “Type”, B.M. Type 3.a.164”, “*Gasteruption poecilothecus* Kieff.”, “[Far East Russia or North China], Amoor [= Amur River= Heilongjiang] / 71 25”, “Determined by Dr. Kieffer”.

#### Additional material.

1 ♀ (NWUX), “NW China: Shaanxi, Miaojv, Liulin, Yaozhou, Tongchuan, sweep net [trapped in spider web], 35.60°N, 108.49°E, 27.vii.2019, 934 m alt., Jiangli Tan, NWUX”; 1 ♀ (RMNH), “NW China: Shaanxi, Huaishuzhuang Rev. St., Ziwuling NNR, Fuxian, Yanan, sweep net, 35.86°N, 108.74°E, 4.viii.2019, 1271 m alt., Jiangli Tan, NWUX”; 1 ♂ (NWUX) from Inner Mongolia.

#### Distribution.

China (Hebei, Heilongjiang, Inner Mongolia, Jilin, Xinjiang); Europe (alpine-boreal); Mongolia; Far East Russia. New for Inner Mongolia and Shaanxi.

#### Notes.

The specimens identified as *G. poecilothecum* (including the holotype) fall within the variation range of *G. subtile*, resulting in its synonymy with the latter species.

**Figures 299–307.**
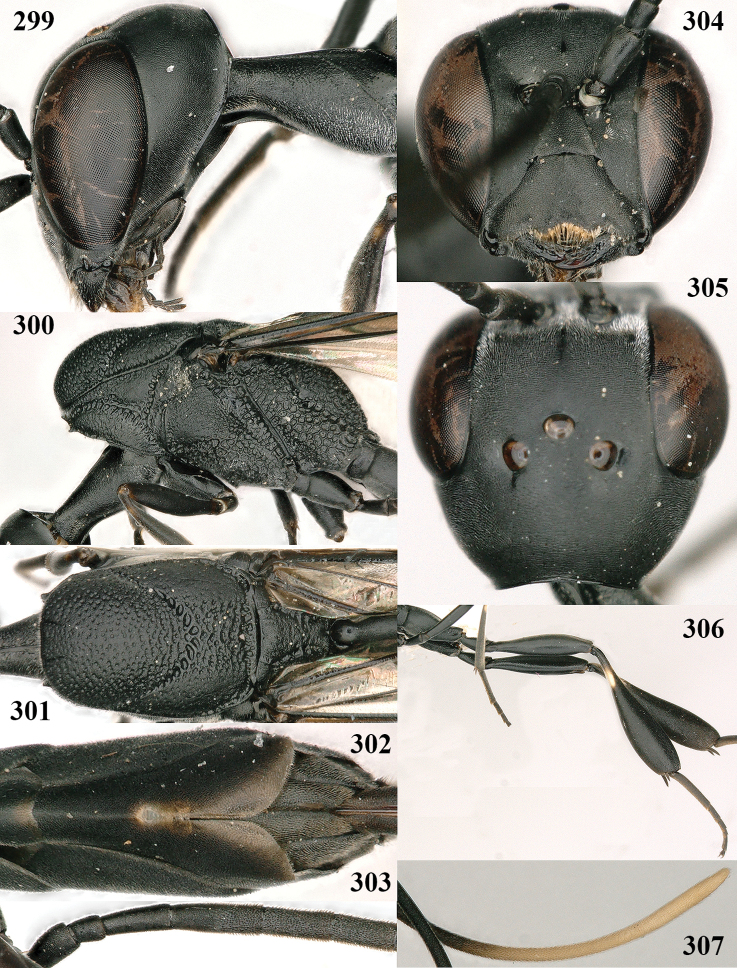
*Gasteruption subtile* Thomson, female, Shaanxi **299** head lateral **300** mesosoma lateral **301** mesosoma dorsal **302** apex of metasoma ventral **303** base of antenna **304** head anterior **305** head dorsal **306** hind leg **307** apex of ovipositor sheath.

**Figures 308–309.**
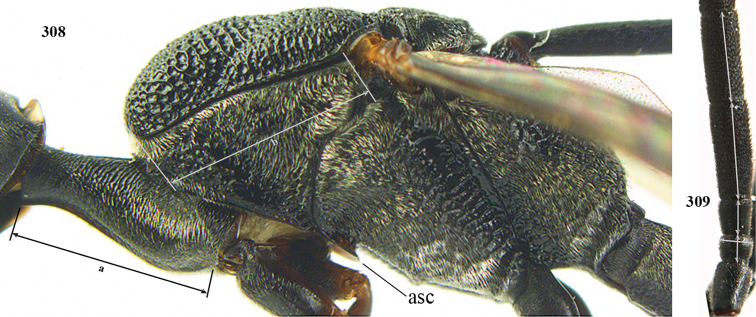
Measurements **308** of the relative length of the propleuron (a) and length of the mesoscutum in front of the tegulum (b) and **309** the length and maximum width of the basal antennal segments; asc = antesternal carina.

## Results and discussion

In this paper, we reviewed the Palaearctic Chinese species of *Gasteruption* and provide a new and extensively-illustrated identification key. The association of the sexes in *Gasteruption* is problematic in several cases and, for recent revisions, the association is based on morphological similarity of the sexes and similar collection data, keeping in mind that males are, in general, more coarsely sculptured than females and have a shorter head in dorsal view. However, smaller males are less sculptured than larger ones and, often, several species occur together at the same locality. In future, presence of DNA data and of reared specimens may solve the problem, but, for the moment, we have to rely on extensive collections per site and precise comparisons of similar specimens. In a few cases, no males are available with distinctive features and probably non-sexual characters of the female are tentatively used for the inclusion in the key as far as possible.

We reported 14 additional species (of which three are new to science) from Shaanxi, bringing the total for Shaanxi to 18 species, which is 46% of the total 39 species known from China. Although coming closer to the 60% hypothesis proposed by Tan et al. (2016), several new species from other parts of China have been seen in collections and about 10 species are still to be expected in Shaanxi, if the hypothesis is correct.

## Supplementary Material

XML Treatment for Gasteruption

XML Treatment for Gasteruption abeillei

XML Treatment for Gasteruption amoyense

XML Treatment for Gasteruption angulatum

XML Treatment for Gasteruption assectator

XML Treatment for Gasteruption bicoloratum

XML Treatment for Gasteruption bimaculatum

XML Treatment for Gasteruption brevicuspis

XML Treatment for Gasteruption coloratum

XML Treatment for Gasteruption corniculigerum

XML Treatment for Gasteruption granulatum

XML Treatment for Gasteruption japonicum

XML Treatment for Gasteruption kexinae

XML Treatment for Gasteruption latitibia

XML Treatment for Gasteruption minutum

XML Treatment for Gasteruption nigritarse

XML Treatment for Gasteruption oshimense

XML Treatment for Gasteruption parvicollarium

XML Treatment for Gasteruption pedion

XML Treatment for Gasteruption reductum

XML Treatment for Gasteruption sinarum

XML Treatment for Gasteruption sinepunctatum

XML Treatment for Gasteruption subtile
